# Dual biomarkers long non-coding RNA GAS5 and microRNA-34a co-expression signature in common solid tumors

**DOI:** 10.1371/journal.pone.0198231

**Published:** 2018-10-05

**Authors:** Eman A. Toraih, Saleh Ali Alghamdi, Aya El-Wazir, Marwa M. Hosny, Mohammad H. Hussein, Moataz S. Khashana, Manal S. Fawzy

**Affiliations:** 1 Genetics Unit, Department of Histology and Cell Biology, Faculty of Medicine, Suez Canal University, Ismailia, Egypt; 2 Center of Excellence of Molecular and Cellular Medicine, Suez Canal University, Ismailia, Egypt; 3 Medical Genetics, Clinical Laboratory Department, College of Applied Medical Sciences, Taif University, Taif, Saudi Arabia; 4 Department of Medical Biochemistry and Molecular Biology, Faculty of Medicine, Suez Canal University, Ismailia, Egypt; 5 Ministry of Health and Population, Cairo, Egypt; 6 Faculty of Medicine, Suez Canal University, Ismailia, Egypt; 7 Department of Biochemistry, Faculty of Medicine, Northern Border University, Arar, Saudi Arabia; University of South Alabama Mitchell Cancer Institute, UNITED STATES

## Abstract

Accumulating evidence indicates that non-coding RNAs including microRNAs (miRs) and long non-coding RNAs (lncRNAs) are aberrantly expressed in cancer, providing promising biomarkers for diagnosis, prognosis and/or therapeutic targets. We aimed in the current work to quantify the expression profile of miR-34a and one of its bioinformatically selected partner lncRNA growth arrest-specific 5 (GAS5) in a sample of Egyptian cancer patients, including three prevalent types of cancer in our region; renal cell carcinoma (RCC), glioblastoma (GB), and hepatocellular carcinoma (HCC) as well as to correlate these expression profiles with the available clinicopathological data in an attempt to clarify their roles in cancer. Quantitative real-time polymerase chain reaction analysis was applied. Different bioinformatics databases were searched to confirm the potential miRNAs-lncRNA interactions of the selected ncRNAs in cancer pathogenesis. The tumor suppressor lncRNA GAS5 was significantly under-expressed in the three types of cancer [0.08 (0.006–0.38) in RCC, *p* <0.001; 0.10 (0.003–0.89) in GB, *p* < 0.001; and 0.12 (0.015–0.74) in HCC, *p* < 0.001]. However, levels of miR-34a greatly varied according to the tumor type; it displayed an increased expression in RCC [4.05 (1.003–22.69), *p* <0.001] and a decreased expression in GB [0.35 (0.04–0.95), *p* <0.001]. Consistent to the computationally predicted miRNA-lncRNA interaction, negative correlations were observed between levels of GAS5 and miR-34a in RCC samples (r = -0.949, *p* < 0.001), GB (r = -0.518, *p* < 0.001) and HCC (r = -0.455, *p* = 0.013). Kaplan-Meier curve analysis revealed that RCC patients with down-regulated miR-34a levels had significantly poor overall survival than their corresponding (*p* < 0.05). Hierarchical clustering analysis showed RCC patients could be clustered by GAS5 and miR-34a co-expression profile. Our results suggest potential applicability of GAS5 and miR-34a with other conventional markers for various types of cancer. Further functional validation studies are warranted to confirm miR-34a/GAS5 interplay in cancer.

## Introduction

Cancer is now the second leading cause of mortality worldwide, causing 8.8 million deaths globally in 2015, which is equivalent to one in every six deaths [1]. Cancer occurs as a net result of activation of oncogenes and inhibition of tumor suppressor genes (TSGs) [2]. Decades back, it was believed that oncogenes and TSGs had to code for proteins, and only mutations in protein-coding genes would result in such pathological conditions as cancer. With the advancement of genetic technologies including next generation sequencing, microarrays and bioinformatic machinery, many truths came to light, including what was originally thought to be junk DNA is now found to code for thousands of equally significant regulatory RNAs [3]. Since their discovery, the non-coding RNAs (ncRNAs) have been recognized as epigenetic regulators of protein-coding genes. Recently, a whole new level of regulation has been uncovered; when it was found that ncRNAs have the ability to regulate each other as well [4] further adding to the complexity of the regulatory processes. This set of findings has revolutionized our understanding of several human diseases, making this ‘the era of non-coding RNAs’.

Many classes of ncRNAs have been identified and linked to cancer, the most common of which are micro RNAs (miRNAs), long-noncoding RNAs (lncRNAs), PIWI-interacting RNAs (piRNAs) and small nucleolar RNAs (snoRNAs). The roles of these four types of ncRNAs in cancer are reviewed in several studies [5–8]. In brief, overexpression of some ncRNAs as miRNAs or lncRNAs can suppress the expression of TSG targets, while loss of function or reduced expression of others may allow overexpression of the oncogenes they regulate. Furthermore, since each ncRNA may regulate hundreds of different genes, its over or under expression may have widespread oncogenic effects because many genes will be dysregulated [9].

Here we were interested in a relatively newly discovered lncRNA; Growth Arrest Specific 5 (GAS5) which is a poorly conserved gene mapped to chromosome 1q25.1 [10]. It consists of 12 exons and 11 introns from which 29 transcripts are produced from alternative splicing, many of which contain retained introns (Ensembl Genome Browser 'GAS5'). The first two transcripts discovered (produced from alternative splice sites on exon 7) are the GAS5a and GAS5b with the latter being predominantly expressed in most cells [11]. Another synonym for the gene is’ Small Nucleolar RNA Host Gene’, this is for having the ability to produce multiple (10 in human) non-coding small nucleolar RNAs (snRNAs) from its more conserved introns [11]. These snRNAs are involved in the regulation of ribosomal RNA (rRNA) synthesis through 2-O-methylation of pre-ribosomal RNA [12].

As its name implies, GAS5 is over expressed in growth-arrested cells [13]. This is further demonstrated by the presence of high levels of GAS5 in brain cells, which are considered the slowest dividing cells in the body as opposed to its lowest levels in other rapidly dividing cells, the most important of which are cancer cells [14].

Expectedly, GAS5 under expression was found to be associated with multiple types of cancer [15–25] and it was found to bind some miRNAs, including miR-21, miR-222 and miR-103 sponging their inhibitory effect on their target TSGs [16, 21, 26, 27].

Our bioinformatic analyses have revealed a potential new miRNA target for the GAS5 gene; miR-34a. This miRNA is encoded from chromosome1p36.22. Its promotor is recognized for having multiple CpG islands and a p53 binding site, making p53 a direct transcriptional regulator for this miRNA [28, 29]. While the history of miR-34a with cancer is very well established, studies conducted on different types of cancers have contradictory results regarding its actual role in tumor progression. For instance, many studies have proved it’s functioning as a TSG in various types of cancer, including neuroblastoma [30], leukemia [31], pancreatic [32] and hepatocellular [33, 34] carcinoma, glioblastoma (GB) [35, 36], breast [37] lung [38] and colon [39] cancer. On the contrary, other studies have found that it functions as an oncogene through promoting tumorigenesis as in RCC [40, 41], papillary thyroid carcinoma (PTC) [42], colon [39] and uterine cancer [43]. It has been suggested that this discrepancy may be attributed to the tissue type and the miR-34a/p53 pathway involved [41].

To the best of our knowledge, no clinical studies were conducted to explore both GAS5 and miR-34a profiles in cancer patients. Hence, we were interested to investigate the expression profiles of these ncRNAs in three prevalent types of cancer in our region; renal cell carcinoma (RCC), hepatocellular carcinoma (HCC) and glioblastoma (GB) as well as to correlate these expression profiles with the available clinicopathological data in an attempt to clarify their roles in cancer.

## Materials and methods

### Genomic characterization of GAS5 and miR-34a

Chromosomal localization, genomic sequence and structure analysis, subcellular localization, variant analysis, and folding pattern were retrieved from different online tools; including Ensembl (http://www.ensembl.org/), GeneCards for human gene database (http://www.genecards.org/), National Center for Biotechnology Information (NCBI) (ncbi.nlm.nih.gov/), COMPARTMENTS subcellular localization (https://compartments.jensenlab.org/Search), Database of Transcription Start Sites (DBTSS) version 10.0 (http://dbtss.hgc.jp/), KineFold (http://kinefold.curie.fr/), and MFold webserver (http://unafold.rna.albany.edu/?q=mfold/RNA-Folding-Form).

### Exploring miR-34 and GAS5 interactions

Identifying complementary regions between microRNA-34a and lncRNA GAS5 were demonstrated by several tools; RNA22 microRNA target detection (https://cm.jefferson.edu/rna22/) and DIANA-LncBase v2 (http://diana.imis.athena-innovation.gr/DianaTools/index.php).

### Functional enrichment analysis

Pathway enrichment analysis and gene ontology of microRNA-34a was performed by Diana-miRPath v3.0 (http://diana.imis.athena-innovation.gr/DianaTools/index.php) using its experimentally validated gene targets. Functional enrichment analysis of GAS5 was obtained from Ensembl and GeneCards databases to identify its biological function in cancer.

### Study population and sampling collection

In total, 230 samples were analyzed, including 60 formalin-fixed, paraffin-embedded (FFPE) RCC samples and their paired adjacent non-cancer tissues, 50 FFPE GB specimens and 10 non-cancer brain tissues as well as 30 HCC blood samples and 20 controls.

#### (a) RCC cohort

The archived FFPE renal samples were taken from sixty patients who underwent radical nephrectomy for a primary RCC and dating back for three years. All retrieved cases were archived in the Pathology laboratory of Mansoura Oncology Center, Mansoura, Egypt. No history of receiving neoadjuvant chemotherapy or radiotherapy prior to sampling. Clinicopathological data, including the survival were collected from patient medical records. Paired sixty cancer-free adjacent tissues were examined and sectioned to serve as controls for molecular analysis.

#### (b) GB cohort

Fifty glioblastoma patients and 10 non-cancer brain tissues collected from the archive of the Pathology Department, Mansoura University Hospitals, Egypt, dating back for three years were included in the current work. Detailed patients' data were retrieved from their follow up records. They had GB grade 4, undergone surgical removal and had not received any treatment before sampling. They followed-up for more than 3 years.

#### (c) HCC cohort

Following our local hospital and medical ethical committee rules in liver tissue sampling prohibition from HCC patients, only blood samples were available. Thirty HCV-induced HCC and 20 matched controls from healthy blood bank donors were recruited in the study. Patients were obtained from the outpatient clinic of Tropical Medicine and Gastroenterology Department, Faculty of Medicine, Assuit, Egypt. They had typical imaging findings of liver cancer and elevated alpha fetoprotein (AFP). Patients underwent clinical and radiological assessment, confirmation of HCV by PCR, Barcelona-Clinic Liver Cancer (BCLC) staging, and Child-Turcotte-Pugh (CTP) scoring [44]. Survival data for HCC patients were not available in patients' medical records. Hence, these data were not included in the statistical analysis for HCC patients.

### Ethical approval

The study was conducted according to the ethical guidelines of the Declaration of Helsinki and approved by the Medical Research Ethics Committee of Suez Canal University, Faculty of Medicine.

### RNA extraction

Total RNA; including the small RNA, was isolated from either FFPE tissue sections (5 to 8-μm-thick) using the Qiagen miRNeasy FFPE Kit (Qiagen, cat no. 217504) or serum using Qiagen miRNeasy serum/plasma Kit (Qiagen, cat no 217184) following the protocols supplied by the manufacturer. Concentration of RNA was determined using the NanoDrop ND-1000 spectrophotometer (NanoDrop Tech., Inc. Wilmington, DE, USA). Samples with a 260/280 nm absorbance ratio less than 1.8 were excluded.

### Reverse transcription reaction

Subsequently, RNA for lncRNA GAS5 was converted to complementary DNA (cDNA) in a T-Professional Basic, Biometra PCR System (Biometra, Goettingen, Germany) using high Capacity cDNA Reverse Transcription Kit (Applied Biosystems, P/N 4368814) with RT random primers as previously described [45].

Reverse transcription of miR-34a was specifically converted to cDNA using TaqMan MicroRNA Reverse Transcription kit (P/N 4366596; Applied Biosystems, Foster City, CA, USA) with 5x miRNA specific stem–loop primers as previously described in our prior publication [46]. Appropriate controls were included in each experiment.

### LncRNA GAS5 and microRNA-34a expression analyses

The Real-Time PCR reactions were performed in accordance with the Minimum Information for Publication of Quantitative Real-Time PCR Experiments (MIQE) guidelines [47]. The expression level of *GAS5* was assessed via SYBR Green qPCR analysis and normalized with *GAPDH* in liver, renal, and brain cancer samples. The following primers were designed using Primer 3 software and checked by *in silico* PCR amplification of the University of California, Santa Cruz (UCSC) genome browser; for *GAS5*: Forward: 5′- CTTGCCTGGACCAGCTTAAT-3′; Reverse: 5′-CAAGCCGACTCTCCATACCT-3′, and for *GAPDH*: Forward: 5'- CGGATTTGGTCGTATTGGG-3'; Reverse: 5'- CTGGAAGATGGTGATGGGATT-3'. In brief, 10 μl of qPCR Green Master (Jena Bioscience, Cat no. PCR-313L), 0.6 μl (10 μM) forward and reverse primers, 8 μl PCR grade water, and 1 μl cDNA template were included in the reaction for *GAS5* and *GAPDH* SYBR Green assay analyses [45, 46]. For microRNA quantification, a final volume of 20 μl was adjusted in duplicate, including 1.33 μl specific RT products, 2× TaqManUniversal PCR Master Mix with UNG (Applied Biosystems, P/N 4440043), and 20× of 1 μl specific TaqManRNA assay for hsa-miR-34a-5p (Applied Biosystems, assay ID 000426) compared to the endogenous control RNU6B (assay ID Hs001093) [48]. Three other endogenous control assays were used in brain cancer [49, 50] and TATA box binding protein (*TBP*; TaqMan assay ID Hs00427620_m1) replaced *GAPDH* for normalization based on our prior work which showed its consistency across samples [36]. Appropriate negative and positive controls were used. A negative control without template was included in each run to exclude contamination of PCR product with genomic DNA. Positive control samples were reapplied in each experiment to test the quality and consistently of the PCR reaction. The PCR for 96-well plates was carried out using StepOne Real-Time PCR System (Applied Biosystem) and incubated as follows: 95°C for 10 min followed by 45 cycles of 92°C for 15 seconds and 60°C for 1 minute. Ten percent randomly selected study samples were re-evaluated in separate runs for the study gene expressions to test the reproducibility of the qPCR which showed very close quantitative cycle value results and low standard deviations.

### Statistical analysis

R package (version 3.3.2) and Statistical Package for the Social Sciences (SPSS) for Windows software (version 23.0) were used for data analyses. Categorical variables were compared using the Chi-square (χ^2^) or Fisher's exact tests where appropriate. Wilcoxon matched-pair signed-rank and Mann-Whitney U tests were used for tissues of renal/brain cancer and serum of liver cancer patients, respectively, to compare continuous variables. The correlation between miR-34a level and *GAS5* expressions was calculated by Spearman’s rank correlation analysis. A two-tailed *p-*value of < 0.05 was considered statistically significant. The receiver operating characteristic (ROC) curves were performed to get the best cutoff values of miRNA-34a for discriminating long and short survivors in cancer patients. The fold change of ncRNAs expressions in each patient relative to the control was calculated via Livak method based on the quantitative cycle (Cq) values with the following equation: relative quantity = 2^−ΔΔ*Cq*^; where ΔΔ*C*_*q*_ = (C_q_ ncRNA–C_q_ internal control) _cancer_ − (C_q_ ncRNA–C_q_ internal control) _control_ [51]. Univariate analysis for association between ncRNA expression profile and clinico-pathological features in cancer patients was run. The software package named PC-ORD version 6 [52] was employed to run different multivariate analyses for clustering analysis of patients according to clinico-pathological and molecular data.

## Results

### Genomic location of GAS5

**Fig 1:**
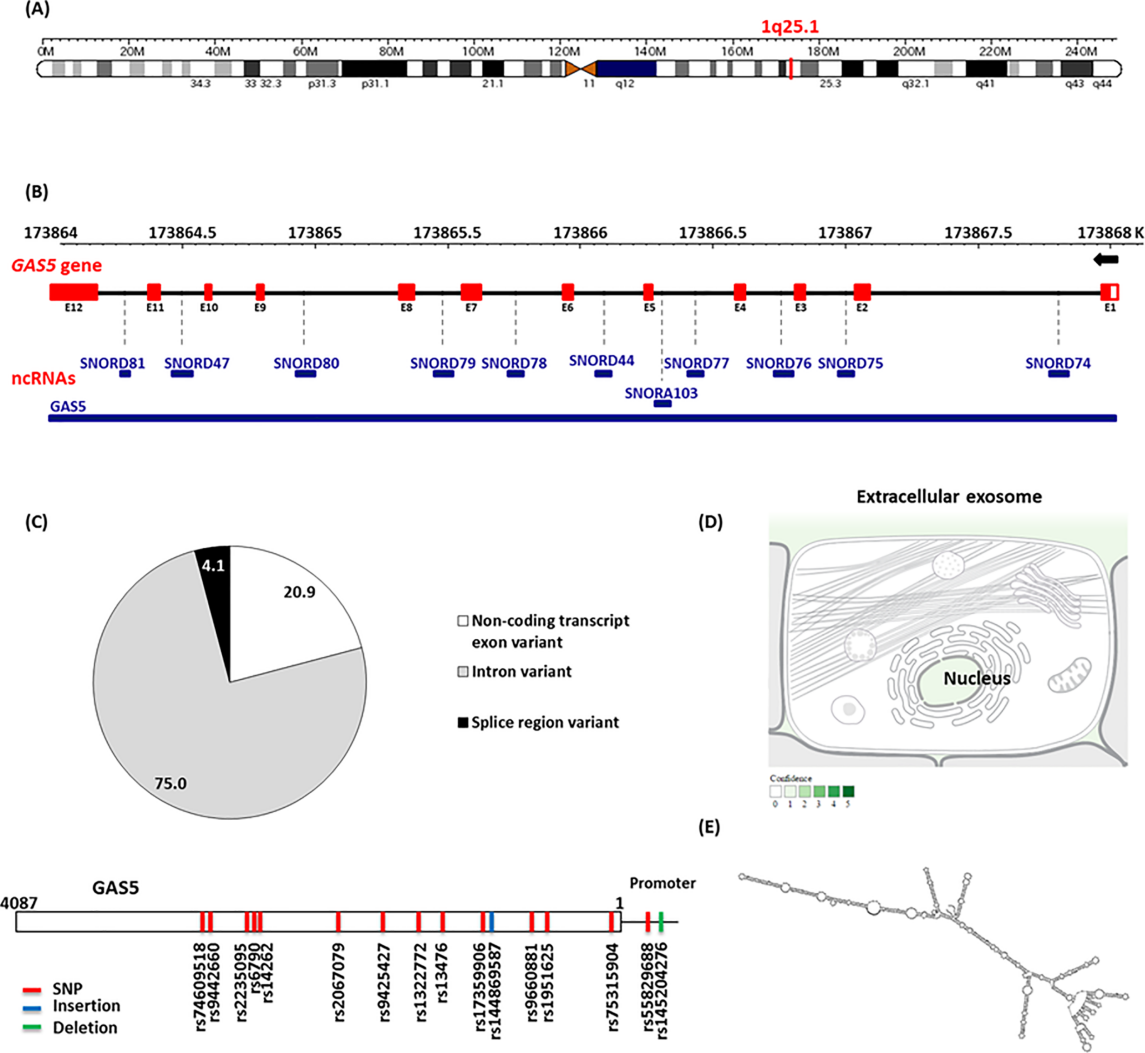
Structural analysis of *GAS5* gene. (A) Chromosomal localization of *GAS5* gene. It is present on chromosome 1q25.1, at genomic coordination 1:173863901–173867987 on the negative strand (according to human genome assembly GRCh38). (B) Sequence analysis of *GAS5* gene. It consists of 12 exons, spanning 4.087 kb that code for 29 different alternative splice transcripts. It hosts multiple snoRNA genes within its introns (except intron 9). These genes encode for ten C/D box snoRNAs, which contain the C (UGAUGA) and D (CUGA) box motifs, and an H/ACA box snoRNA, SNORDA103, within intron 4. (C) Genetic variant analysis. *GAS5* gene (4087 bases long) contains around 300 thousand variants (75% intronic, 21% exonic, and 4% splice region polymorphisms). Among all these polymorphisms, 14 SNPs (red), one deletion (green) and one insertion (blue) are common variants with minor allele frequency (MAF) > 0.05. (D) Subcellular localization of lncRNA GAS5. Text mining highlighted its predominant existence intranuclear and within extracellular exosomes which are extruded into the circulation. (E) Folding pattern of lncRNA GAS5. [Data source: Ensembl.org, genecards.org, NCBI, COMPARTMENT database, and MFold].

Lnc-RNA GAS5 is also known as small nucleolar RNA host gene 2 (SNHG2) and non-protein coding RNA 30 (NCRNA00030). It is encoded by GAS5 gene on chromosome 1q25.1 (Genomic coordination at 1:173863901–173867987 at the negative strand according to human genome assembly GRCh38) (**Fig 1A**). It consists of 12 exons, spanning 4.087 kb and encoding for 29 different alternative splice transcripts ranging in length from 242 to 1698 bp (**S1 Table**).

### Sequence analysis of *GAS5*

*GAS5* gene contains a seven-nucleotide oligo-pyrimidine tract on its 5′-end in exon 1, hence is classified as a member of the 5' terminal oligo-pyrimidine (TOP) genes. This sequence can act as a cis-regulatory motif which either inhibits the binding of translational regulatory proteins downstream to the transcriptional start sites of mRNAs or suppresses the translational machinery itself. In addition to its translational controls, the TOP elements are known to modulate gene expression through regulating transcription [53]. Being one of the TOP genes, it is ubiquitously expressed and is predicted to regulate the translation of more than 20% of total mRNAs [DataBase of Transcription Start Sites]

Sequence analysis of *GAS5* gene revealed that it is a small nucleolar RNA host gene, containing multiple *snoRNA* genes within its introns. These genes encode for ten C/D box snoRNAs, which contain the C (UGAUGA) and D (CUGA) box motifs. They are predicted to play a role in the 2'-O-methylation of rRNA by guiding guanine methylation, which enhances RNA folding and interaction with ribosomal proteins. *GAS5* also hosts SNORDA103 within intron 4, an H/ACA box snoRNA, which is associated with pseudouridylation (**Fig 1B**).

### Variant analysis

*GAS5* gene is shown to be highly polymorphic, enclosing around 300 thousand variants (75% intronic, 21% exonic, and 4% splice region polymorphisms). Among all these polymorphisms, 14 SNPs, one deletion and one insertion are common variants with minor allele frequency (MAF) > 0.05 (**Fig 1C**) and (**S2 Table**).

### GAS5-miRNA interaction

Analysis with the RNA22 program (http://cbcsrv.watson.ibm.com/rna22.html) identified complementary regions of GAS5 with 690 microRNAs. Among these putative microRNAs, only 252 interactions with 234 microRNAs showed significant binding (p<0.05) (**S3 Table**). Via DIANA-LncBase v2 database for experimentally validated miRNA-lncRNA interactions, GAS5 was identified as a miR-34a target by immunoprecipitation experiments (score = 0.558). For further confirmation, we used RNA22 software to determine the interaction binding sites between GAS5 transcripts and miR-34a. Our results showed base-pairing in twenty-three alternative splice variants. Among them, nine transcripts had two miR-34a binding sites (**S4 Table**).

#### miR-34a functional analysis

**Fig 2:**
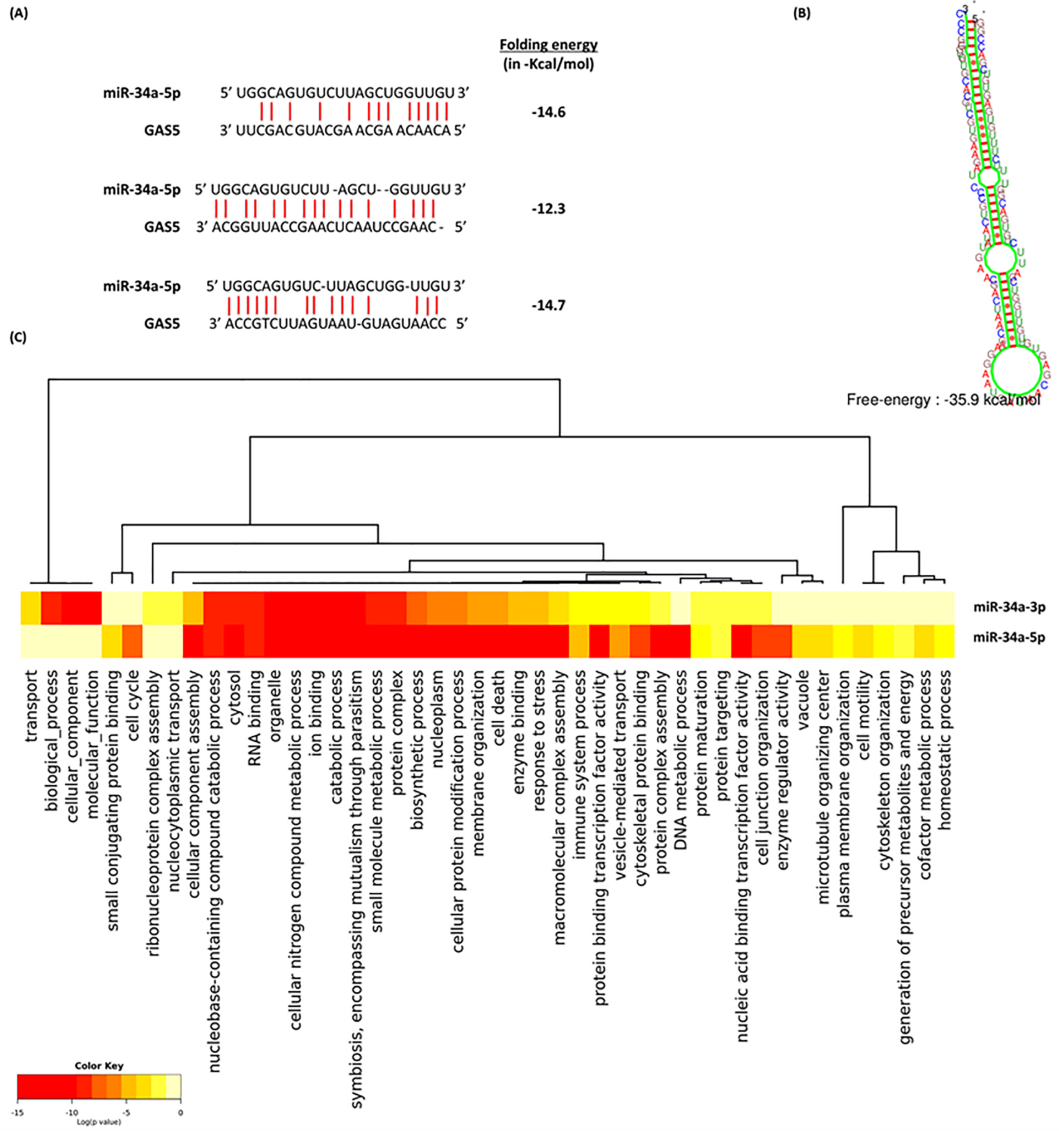
Functional and structural analysis of miR-34a. (A) GAS5: miR-34a-5p interaction. Complementarity regions are shown in three binding sites in *GAS5* gene, one proximal at 5' region (1432–1455) and two distal at 3' end (3545–3567 and 3698–3719) [Data source: RNA22, DIANA-LncBase v2]. (B) Predicted secondary structure of miR-34a. Folding pattern and energy are demonstrated [Data source: KineFold]. (C) Functional enrichment analysis of miR-34a experimentally validated gene targets. Significant clustered heat map represents the gene ontology using GO slim option, FDR conservative states, *p* value threshold < 0.05, and categories union [Data source: DIANA-miRPath v3]. http://snf-515788.vm.okeanos.grnet.gr/#mirnas=hsa-miR-34a-3p;hsa-miR-34a-5p&methods=Tarbase;Tarbase&selection=2.

Hundreds of miR-34a-5p and 3p targets were retrieved from various online databases; including miRTarBase (http://mirtarbase.mbc.nctu.edu.tw/), miRDB (http://mirdb.org/) and microRNA.org. Functional enrichment analysis revealed its enrollment in cancer-related KEGG pathways, as Pathways in cancer (hsa05200, 115 targets, p = 0.001723304), proteoglycans in cancer (hsa05205, 71 targets, p = 1.687731e-06), adherens junction (hsa04520, 34 targets, *p* = 3.396929e-06), cell cycle (hsa04110, 54 targets, *p* = 3.355808e-05), and p53 signaling pathway (hsa04115, 31 targets, *p* = 0.006060223) as well as cancer-specific pathways, namely glioma (hsa05214, 29 targets, *p* = 0.0003111577) and renal cell carcinoma (hsa05211, 28 targets, p = 0.02521455) (**S5 Table**). GO analysis (Diana tools) demonstrated miR-34a to be involved in cell death, cell cycle, and response to stress thus highlighting its role in cancer cell growth. In addition, miR-34a was significantly associated with membrane organization, cell junction organization, and cell motility, hence may play a key role in cancer cell invasion and metastasis (**Fig 2**).

### Characteristics of the study population

Baseline characteristics of RCC, GB, and HCC cohorts are demonstrated in **Tables 1**–**3**.

**Table 1:** Clinicopathological characteristics of renal cell carcinoma patients.

Variables	Total	Low OS	High OS	*p* value
**Total number**	60 (100)	19 (31.7)	41 (68.3)	
**Age**				
≤55 years	6 (10.0)	3 (15.8)	3 (7.3)	0.370
>55 years	54 (90.0)	16 (84.2)	38 (92.7)	
**Sex**				
Female	21 (35.0)	9 (47.4)	1 (29.3)	0.245
Male	39 (65.0)	10 (52.6)	29 (70.7)	
**HPD**				
Clear cell	30 (50.0)	8 (42.1)	22 (53.7)	0.354
Papillary	15 (25.0)	4 (21.1)	11 (26.8)	
Chromophobic	15 (25.0)	7 (36.8)	8 (19.5)	
**Tumor location**				
Right side	22 (36.7)	7 (36.8)	15 (36.6)	0.985
Left side	38 (63.3)	12 (63.2)	26 (63.4)	
**Grade**				
Grade 1	9 (15.0)	1 (5.3)	8 (19.5)	**0.023**
Grade 2	28 (46.7)	6 (31.6)	22 (53.7)	
Grade 3	23 (38.3)	12 (63.2)	11 (26.8)	
**Tumor size**				
T1	21 (35.0)	6 (31.6)	15 (36.6)	0.589
T2	25 (41.7)	7 (16.8)	18 (43.9)	
T3	14 (23.3)	6 (31.6)	8 (19.5)	
**LN**				
Negative	39 (65.0)	11 (57.9)	28 (68.3)	0.562
Positive	21 (35.0)	8 (42.1)	13 (31.7)	
**Recurrence**				
Negative	44 (73.3)	7 (36.8)	37 (90.2)	**<0.001**
Positive	16 (26.7)	12 (63.2)	4 (9.8)	
**DFS**				
≤ 1year	29 (48.3)	19 (100)	10 (24.4)	**<0.001**
> 1year	31 (51.7)	0 (0.0)	31 (75.6)	
**GAS5 fold**				
≤ 1-fold	55 (91.7)	19 (100)	36 (87.8)	0.283
> 1-fold	3 (5.0)	0 (0.0)	3 (7.3)	
>10-folds	2 (3.3)	0 (0.0)	2 (4.9)	
**miR-34a fold**				
≤ 1-fold	15 (25.0)	6 (31.6)	9 (22.0)	0.192
> 1-fold	19 (31.7)	8 (42.1)	11 (26.8)	
>10-folds	26 (43.3)	5 (26.3)	21 (51.2)	

*HPD* histopathological diagnosis, *DFS* disease-free survival, *OS* overall survival. Low OS: ≤12 months, High OS: >12 months. Fisher's Exact and Pearson Chi-square tests were used. Bold values indicate statistical significance at *p* < 0.05

**Table 2:** Clinicopathological characteristics of glioblastoma patients.

Variables	Total	Low OS	High OS	*p* value
**Total number**	50 (100)	10 (20.0)	40 (80.0)	
**Age**				
≤55 years	21 (42.0)	7 (70.0)	14 (35.0)	0.073
>55 years	29 (58.0)	3 (3.0)	26 (65.0)	
**Sex**				
Female	13 (26.0)	1 (10.0)	12 (30.0)	0.258
Male	37 (74.0)	9 (90.0)	28 (70.0)	
**Tumor location**				
Frontal	24 (48.0)	8 (80.0)	16 (40.0)	0.071
Tempero-paroetal	18 (36.0)	1 (10.0)	17 (42.5)	
Fronto-temporal	8 (16.0)	1 (10.0)	7 (17.5)	
**Recurrence**				
Negative	44 (88.0)	8 (80.0)	36 (90.0)	0.586
Positive	6 (12.0)	2 (20.0)	4 (10.0)	
**DFS**				
≤ 1year	25 (50.0)	10 (100)	15 (37.5)	**0.001**
> 1year	25 (50.0)	0 (0.0)	25 (62.5)	
**GAS5 fold**				
≤ 1-fold	37 (74.0)	7 (70.0)	30 (75.0)	0.707
> 1-fold	13 (26.0)	3 (30.0)	10 (25.0)	
>10-folds				
**miR-34a fold**				
≤ 1-fold	38 (76.0)	8 980.0)	30 (75.0)	0.741
> 1-fold	12 (24.0)	2 (20.0)	10 (25.0)	
>10-folds	0 (0.0)			

Data are presented as number (frequency). *HPD* histopathological diagnosis, *DFS* disease-free survival, *OS* overall survival. Low OS: ≤12 months, High OS: >12 months. Fisher's Exact and Pearson Chi-square tests were used. Bold values indicate statistical significance at *p* < 0.05

**Table 3:** Clinicopathological characteristics of hepatocellular carcinoma patients.

Variables	Patients (n = 30)
**Total number**	
**Age**	
≤55 years	13 (43.3)
>55 years	17 (56.7)
**Sex**	
Female	11 (367)
Male	19 (63.3)
**Tumor size**	
<5 cm	9 (30.0)
≥5 cm	21 (70.0)
**Number of lesions**	
Solitary	20 (66.7)
Multiple	10 (33.3)
**LCF score**	9.7±1.2
**CTP class**	
Child B	21 (70.0)
Child C	9 (30.0)
**Hepatomegaly**	
Mild	18 (60.0)
Massive	12 (40.0)
**Treatment**	
Ablation	15 (30.0)
Radiofrequency	11 (36.7)
Supportive	4 (13.3)
**GAS5 fold**	
≤ 1-fold	27 (90.0)
> 1-fold	3 (10.0)
**miR-34a fold**	
≤ 1-fold	19 (63.3)
> 1-fold	10 (33.3)

Data are presented as number (frequency). *CTP* Child-Turcotte-Pugh classification for liver cell failure, *LCF* liver cell failure score by CTP classification. Fisher's Exact and Pearson Chi-square tests were used.

### Expression profiling

**Fig 3:**
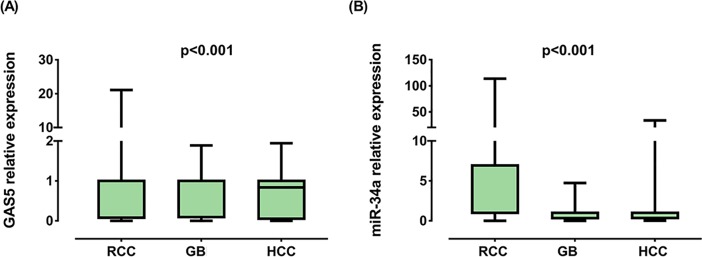
Relative expression of GAS5 and miR-34a-5p in cancer. RCC renal cell carcinoma, GB glioblastoma, HCC hepatocellular carcinoma. Data are represented as medians. The box defines upper and lower quartiles (25% and 75%, respectively) and the error bars indicate upper and lower adjacent limits. Expression levels in cancer and control samples were normalized to GAPDH in RCC and HCC, TBP in GB and RNU6B for microRNA and calculated using the delta-delta CT method [= 2^(-ΔΔCT)^] in comparison to controls. Fold change of controls were set at 1.0. Wilcoxon matched-pair signed-rank and Mann-Whitney U tests were used for tissues of renal/brain cancer and serum of liver cancer patients, respectively. Two-sided *p* < 0.05 was considered statistically significant.

**Fig 4:**
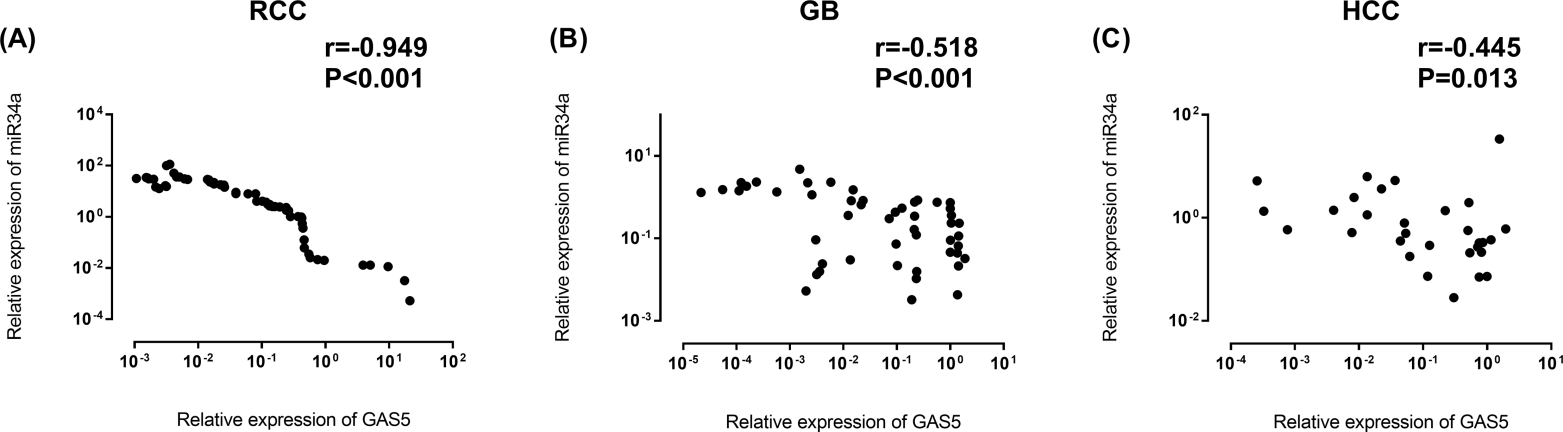
Correlation between GAS5 and miR-34a-5p expression levels. RCC renal cell carcinoma, GB glioblastoma, HCC hepatocellular carcinoma. Data were log transformed. Spearman’s rank correlation test was used. Statistical significance was considered at *p* < 0.05.

GAS5 levels were under-expressed in RCC [0.08 (0.006–0.38), *p* <0.001], GB [0.10 (0.003–0.89), *p* < 0.001] and HCC [0.12 (0.015–0.74), *p* < 0.001]. On the other hand, miR-34a displayed an increased expression in RCC [4.05 (1.003–22.69), *p* < 0.001] and a decreased expression in GB [0.35 (0.04–0.95), *p* < 0.001] as depicted in **Fig 3**. Aligned with the computationally predicted interaction between microRNA and lncRNA in our bioinformatics analysis, a very strong correlation between the expression profile of GAS5 and miR-34a was detected in RCC samples [r = -0.949, *p* < 0.001] and a moderate negative correlation was observed between levels of GAS5 and miR-34a in GB [r = -0.518, *p* < 0.001] and HCC [r = -0.455, *p* = 0.013] (**Fig 4**).

### Association of GAS5 and miR-34a with clinicopathological features

Univariate analyses are shown in **Table 4A**, **4B** and **4C**. ROC curves showed no prognostic value for GAS5 nor miR-34a to predict survival of cancer patients in RCC and GB, or to predict CTP class for liver cell failure in HCC patients (*p* > 0.05) (**S1 Fig**).

**Table 4:** Univariate analysis for association between gene profile and clinico-pathological features in the study cohorts.

	**(a) RCC**				
	**GAS5**			**miR-34a**	
	***p***_**ass**_		**r (*p***_**corr**_**)**	***p***_**ass**_	**r (*p***_**corr**_**)**
**Age**	0.239		-0.069 (0.598)	0.352	-0.066 (0.619)
**Sex**	0.631		0.063 (0.635)	0.798	-0.033(0.801)
**Location**	0.908		-0.015 (0.910)	0.884	0.019 (0.886)
**HPD**	0.257		0.191 (0.143)	0.504	-0.130(0.321)
**Grade**	0.402		0.171 (0.192)	0.378	-0.167 (0.204)
**Tumor size**	0.199		0.157 (0.230)	0.361	-0.152 (0.246)
**LN**	0.193		0.169 (0.195)	0.258	-0.147(0.261)
**Recurrence**	0.192		0.170 (0.195)	0.371	-0.116 (0.376)
**DFS**	0.120		**0.305 (0.018)**	0.363	0.215 (0.099)
**OS**	0.266		**0.283 (0.029)**	0.441	0.191 (0.144)
	**(b) GB**				
**Age**	0.883		0.063 (0.663)	0.930	-0.013 (0.931)
**Sex**	0.921		-0.014 (0.922)	0.514	-0.093 (0.520)
**Location**	0.530		-0.150 (0.300)	0.711	0.091 (0.531)
**Recurrence**	0.258		0.166 (0.248)	0.070	-0.260 (0.068)
**DFS**	0.869		0.012 (0.936)	0.677	0.074 (0.609)
**OS**	0.451		0.48 (0.743)	0.356	0.013 (0.927)
	**(c) HCC**				
**Age**	0.869	-0.037 (0.848)		0.059	-0.289 (0.121)
**Sex**	0.553	0.112 (0.556)		0.420	0.156 (0.411)
**Hepatomegaly**	0.692	-0.079 (0.680)		0.851	0.039 (0.837)
**Tumor size**	0.226	0.278 (0.137)		0.209	-0.315 (0.090)
**Number of lesions**	0.193	-0.352 (0.056)		0.916	0.112 (0.225)
**CTP class**	0.283	-0.206 (0.275)		0.533	0.122 (0.521)
**Treatment**	0.715	0.144 (0.447)		0.598	0.006 (0.973)

*p* ass *p* values for association, *r* (*p* corr) correlation spearman's coefficient and *p* value of correlation, *HPD* histopathological diagnosis, *T* tumor size, *LN* lymph node, *DFS* disease-free survival, *OS* overall survival, *CTP* Child-Turcotte-Pugh classification for liver cell failure. Spearman's rank, Mann-Whitney U and Kruskal-Wallis tests were used. Statistically significant values (*p* < 0.05) are shown in bold.

### Survival analysis in RCC and GB

In RCC, multivariable analysis using logistic regression test (Enter method) showed age and pathological grade to be independent predictors for recurrence: (OR = 1.251, 95% CI = 1.075–1.455, *p* = 0.004) for the age and (OR = 19.9, 95% CI = 1.034–383, *p* = 0.047) for the grade. Kaplan-Meier curve analysis and log-rank test revealed that RCC patients with female gender, post-nephrectomy recurrence, advanced pathological grade, and down-regulated miR-34a levels had significantly poor overall survival than their corresponding (*p* < 0.05) (**Table 5**). In addition, multivariable analysis by Cox regression model demonstrated sex and the recurrence to be independent predictors of overall survival (hazard ratio (HR) = 2.49; 95% confidence interval (95% CI) 1.14–5.41, *p* = 0.021) and (HR = 4.16; 95% CI of 1.88–9.16, *p* < 0.001), respectively.

**Table 5:** Multivariable analysis in renal cell carcinoma patients.

Variables	Survival time	Overall comparisons			Cox regression	
	OS (mo)	Log Rank	Breslow	Tarone-Ware	HR (95% CI)	*p*
**Age**						
≤55 years	13.1 ± 2.22					
>55 years	16.1 ± 0.8	0.186	0.244	0.221	0.97 (0.92–1.01)	0.217
**Sex**						
Female	13.9 ± 1.28					
Male	16.7 ± 0.94	**0.027**	0.077	0.095	**2.49 (1.14–5.41)**	**0.021**
**HPD**						
Type 1	16.1 ± 1.12					
Type 2	17.5 ± 1.61				0.70 (0.34–1.46)	0.351
Type 3	13.3 ± 1.25	0.103	0.198	0.159	0.69 (0.30–1.60)	0.397
**Location**						
Right side	16.3 ± 1.23					
Left side	15.4 ± 1.00	0.703	0.489	0.539	1.08 (0.57–2.06)	0.807
**Grade**						
Grade 1	16.3 ± 1.53					
Grade 2	18.0 ± 1.17					
Grade 3	12.8 ± 1.05	**0.005**	**0.003**	**0.003**	1.60 (0.66–3.85)	0.293
**Tumor size**						
T1	16.4 ± 1.40					
T2	15.9 ± 1.13				0.91 (0.30–2.75)	0.871
T3	14.5 ± 1.65	0.709	0.577	0.635	0.89 (0.34–2.29)	0.813
**LN**						
Negative	16.4 ±0.97					
Positive	14.6 ± 1.27	0.306	0.235	0.255	1.05 (0.42–2.59)	0.911
**Recurrence**						
Negative	17.5 ± 0.83					
Positive	10.8 ± 1.07	**<0.001**	**<0.001**	**<0.001**	**4.16 (1.88–9.16)**	**<0.001**
**GAS5 fold**						
Under-expressed	15.7 ± 0.84					
Over-expressed	16.2 ± 1.24	0.735	0.753	0.985	0.29 (1.04–0.96)	0.291
**miR-34a fold**						
Under-expressed	13.2 ± 0.99					
Over-expressed	16.6 ± 0.95	**0.010**	**0.049**	**0.024**	1.001 (0.98–1.01)	0.926

Survival times is shown as mean and standard error, *OS* overall survival, *HR (95% CI)* Hazard ratio (95% confidence interval), *HPD* histopathological diagnosis, *T* tumor size, *LN* lymph node. Statistically significant values (*p* < 0.05) are shown in bold.

In GB, Kaplan-Meier curves showed a significant association of shorter survival times with male gender (Breslow test: *p* = 0.002 and Tarane-Ware test: *p* = 0.030). In addition, marginal significance was observed for poor overall survival in patients with frontal lesions (Log rank test: *p* = 0.050) (**Table 6**). Multivariable analysis by Cox regression model illustrated tumor recurrence to be an independent predictor of low overall survival (HZ = 11.1; 95% CI 2.88–42.5, *p* < 0.001).

**Table 6:** Multivariable analysis in glioblastoma patients.

Variables	Survival time	Overall comparisons			Cox regression	
	OS (mo)	Log Rank	Breslow	Tarone-Ware	HR (95% CI)	*p*
**Age**						
≤55 years	17.8 ± 1.4	0.742	0.683	0.595	1.03 (0.97–1.09)	0.298
>55 years	17.0 ± 0.96					
**Sex**						
Female	20.5 ± 1.5	0.096	**0.002**	**0.030**	1.36 (0.45–4.11)	0.583
Male	16.2 ± 0.8					
**Tumor location**						
Frontal	15.7 ± 1.01	**0.050**	0.079	0.062		0.749
Tempero-paroetal	17.6 ± 1.3				1.3 (053–3.39)	0.534
Fronto-temporal	21.5 ± 2.1				1.5 (0.44–5.75)	0.472
**Recurrence**						
Negative	17.6 ± 0.8	0.284	0.397	0.342	**11.1 (2.88–42.5)**	**<0.001**
Positive	15.3 ± 1.8					
**GAS5 fold**						
Under-expressed	17.5 ± 0.9	0.765	0.581	0.642	0.96 (0.49–1.85)	0.904
Over-expressed	16.9 ± 1.6					
**miR-34a fold**						
Under-expressed	17.7 ± 0.9	0.460	0.453	0.415	1.14 (0.79–1.65)	0.471
Over-expressed	16.1 ± 1.5					

Survival time is shown as mean and standard error, *OS* overall survival, *HR (95% CI)* Hazard ratio (95% confidence interval). Statistically significant values (*p* < 0.05) are shown in bold.

### Hierarchical clustering analysis

**Fig 5:**
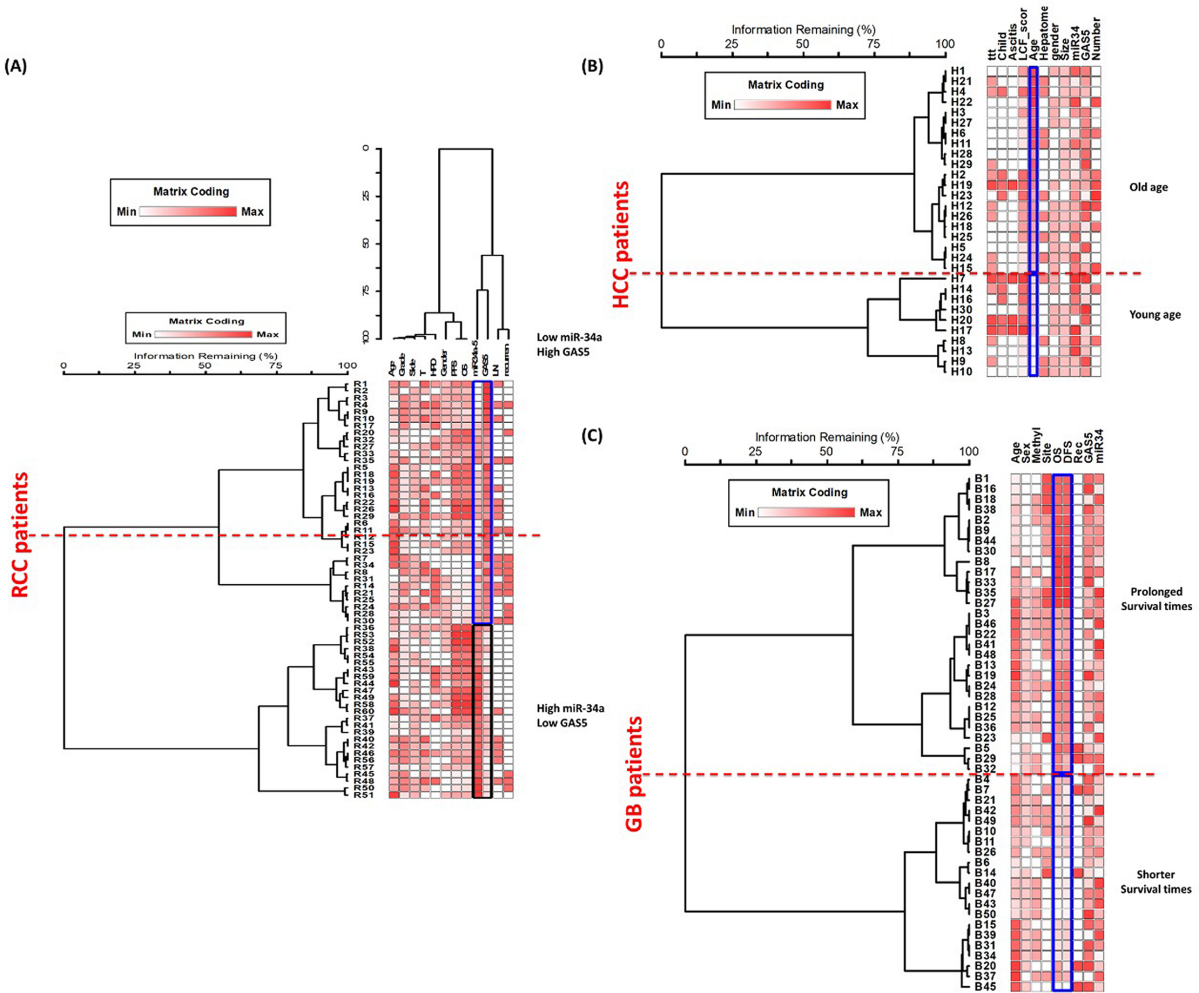
Multivariate analysis of patients according to combined transcriptomic signature of genes and clinicopathological features. **(A) RCC, (B) GB, (C) HCC.** RCC patients were divided based on GAS5 and miR-34a levels, GB patients were divided by survival times and HCC patients by age.

Dendrograms for Two-way agglomerative hierarchical cluster analysis were employed for data exploration (**Fig 5**). The following cluster setup parameters were adjusted: Distance method: Sorensen (Bray-Curtis), Group Linkage Method: Flexible Beta at 0.75, Clustering of factor relative by factor maximum. A distance matrix is shown. RCC plot analyzed 60 strands and 12 factors, GB plot analyzed 50 strands and 9 factors, whereas HCC dendrogram showed results of 30 strands and 11 factors. Clustering analysis revealed separation of RCC patients by GAS5 and miR-34a levels, GB patients by survival times, and HCC patients by their age. However, it is worth noting that Cox regression analysis is more accurate and specific for survival analysis, while the purpose of multivariate analysis is to graphically expose the relationship between cluster analyses and individual data points, making it easy to see similarities and differences between patients of the same cancer types.

## Discussion

In this study, we measured the expression of two ncRNAs; the lncRNA GAS5 and the miRNA miR-34a in three of the most prevalent and high-incidence tumors in Egypt; hepatic, renal and brain cancer. We chose more than one tumor type to assess if the same ncRNA could work differently according to the tissue type. We also investigated the possible association between GAS5 and miR-34a in mediating carcinogenesis after detecting an interaction between the two through our preliminary in *silico* analysis.

Our results show that *GAS5* was under-expressed in the three types of cancer; RCC, HCC and GB. On the other hand, levels of miR-34a greatly varied according to the tumor type. RCC patients had a lower GAS5 level and a higher miR-34a level in tumor tissue compared to adjacent non-cancer tissue. In accordance, Qiao et al. reported a reduced GAS5 level in RCC cell lines compared to normal cell lines. Furthermore, in *vitro* cloning and functional expression analysis revealed that *GAS5* overexpression caused cell cycle arrest, increased apoptosis, as well as inhibited tumor metastatic potential [24], which could explain the correlation between reduced patient survival and GAS5 level in our study. As for miR-34a, several studies reported over-expression of this RNA in RCC patients [40, 41, 54–56]. Liu et al. predicted that its oncogenic function could be through targeting the two tumor suppressors secreted frizzled related protein 1 (SFRP1) and calmodulin binding transcription activator 1 (CAMTA1), and further validated the first target [40]. Another study noticed that miR-34a enhances cell survival, both in *vitro* and in *vivo* during cisplatin nephrotoxicity through p53 [57]. Collectively, the previous studies suggest that miR-34a can act as an oncogene in renal tissue. On the contrary, Yadav et al. detected a significant under-expression of miR-34a in both sera and renal tissues of RCC patients [58]. Similar results were demonstrated by the studies of Zhang et al. and Weng et al. [59, 60], where the former study demonstrated that decreased expression of miR-34a in RCC patients inversely up-regulated the gene for the transcription factor YY1. The latter study suggested that under-expression of miR-34a in cancer tissues of RCC patients affected the regulation of the *NOTCH1* gene, as well as caused dysregulation of other multiple miR-34a targets in 786-O and Caki-1 RCC cell lines. Yu et al. found that miR-34a suppressed tumor growth and metastasis in *vivo* and in *vitro* through targeting CD44 [61]. Another study found that miR-34a inhibits cellular invasion in renal cancer cell lines A498 and 769P through targeting the 3’ untranslated region (UTR) of the *c-myc* oncogene [62]. Also, the lncRNA NEAT1 (nuclear paraspeckle assembly transcript 1) was found to sponge miR-34a releasing its inhibition on the *c*-*met* oncogene, resulting in increased cellular proliferation and invasion in RCC cell lines 786-O and ACHN [63]. Furthermore, Bai et al. found that miR-34a causes senescence of rat renal cells through targeting two anti-oxidative mitochondrial genes [64]. Cellular senescence, which is an irreversible state of growth arrest, protects the cells from accumulating mutations that could lead to malignant transformation [65]. Further supporting the tumor suppressor potential of miR-34a, Zhou et al. provided evidence that miR-34a, secreted by fibroblasts, enhances apoptosis of renal tubular cells through regulating the anti-apoptotic gene *BCL*-*2* [66].

The inconsistent results between our study which shows up-regulation of *miR-34a* in RCC and other studies showing its under-regulation in the same type of cancer could have many possible explanations. First, we measured the expression of miR-34a in cancer tissues obtained directly from RCC patients as opposed to other studies conducted on cancer cell lines. While those cell lines are highly essential for functional molecular analysis, they may be different from primary tumors, possibly through building up new mutations in their attempt to adjust to their artificial environment [67, 68]. Such mutations may easily alter cellular responses and regulatory mechanisms, possibly affecting the expression of *miR*-*34a*. Second, miR-34a could be a non-specific molecule that can both activate or inhibit tumerogenesis depending on the surrounding environment. These include internal stimuli (other regulatory molecules or polymorphisms, oxidative molecules, other associated disease states, tumor stage/grade or else) and external stimuli (cellular response to environmental exposures, including chemotherapy, other drugs, chemicals, foods, etc). Third, miR-34a has multiple targets (discussed in our previous work) [41], as well as being itself a target for many lncRNAs. Each study focuses on one or few targets and infers a tumor suppressor or an oncogenic function based on its effect on the studied target/pathway. However, miR-34a is one small molecule in a larger network of molecules that either promotes or inhibits tumorigenesis based on the net result of *all* its regulated targets, which could easily differ according to countless variables. Given by the negative correlation of this microRNA with GAS5 in our RCC patients, as well as the predicted interaction between the two, we believe that a new pathway; the *GAS5*/miR-34a pathway might be involved in the previously indicated molecular network leading to RCC.

The interaction between lncRNAs and miRNAs can be multifaceted. Yoon et al. explained four mechanisms of interaction between the two types [69]. First, lncRNAs can act as miRNA sponges as previously mentioned with examples in the introduction. Second, the opposite can occur, where miRNAs can inhibit lncRNAs by binding to them and causing their degradation. This applies for miR145-5p, miR-181a-5p and miR-99b-3p which inhibit lncRNA ROR [70], and miR-9 which inhibits lncRNA MALAT1 [71]. Third, miRNAs and lncRNAs can both compete for the same binding site on mRNAs, an example of which is miR-485-5p and lncRNA BACE1AS competing for BACE1 mRNA [72]. Fourth and finally, another form of relationship exists between the two types; where the lncRNA (>200 nucleotide) is capable of generating smaller (<22 nucleotide) miRNAs such as the lncRNA H19 generating miR-675 [73]. GAS5 provides a perfect example for such interactions. For instance, GAS5 acts as a molecular sponge for many different microRNAs, including miR-21, miR-222, miR-196a, miR-205, miR-221 and miR-103. [16, 26, 74–77], all of which are related to cancer. Then again, both miR-21 and miR-222 can negatively regulate *GAS5* [27, 78]. Also, three of the snoRNAs produced by GAS5 (U44, U74 and U78) can give rise to miRNAs [79], making GAS5 one of the lncRNAs generating miRNAs.

While functional validation has yet been required to prove the direct interaction between GAS5 and miR-34a, it is highly plausible. This is due to the already verified interaction between miR-34a and GAS1 (Growth arrest specific 1), another member of the *GAS* genes [42]. *GAS1* is a protein coding gene that, similar to *GAS5*, exerts its tumor suppressor actions through arresting the cell cycle and stimulating apoptosis [80]. Ma et al. measured the expression of miR-34a and the GAS1 protein in papillary thyroid carcinoma; GAS1 was under-expressed, while miR-34a was over-expressed. Further analysis revealed that miR-34a binds to the 3’UTR of GAS1 causing its silencing, which in turn activates the *RET* oncogene [42]. Through the BLAST tool, we detected sequence homology between the *GAS1* and the *GAS5* genes, further raising the possibility of interaction of miRNA-34a with GAS5. Given that *GAS5* is down-regulated and *miR-34a* is up-regulated in our study, tumorigenesis may be in such a case due to sponging of miR-34a by GAS5, where under-expression of *GAS5* in the rapidly dividing cancer cells causes release of the inhibition of miR-34a on its tumor suppressor targets, allowing for further tumor progression. Oppositely, the inverse correlation between the two could also suggest that miR-34a inhibits GAS5, making its under-expression a cause of cancer rather than a result, as in the case of the miR-34a-GAS1 interaction.

In patients with GB, both GAS5 and miR-34a levels were significantly down-regulated. Zhang et al. correlated the expression of five lncRNAs including GAS5 with GB and found that higher levels of GAS5 were associated with prolonged survival [81]. Zhao et al. found that *GAS5* was under-expressed in glioma cell lines U87 and U251 and that its tumor suppressor role was through targeting miR-222 [16]. The same author, in a more recent study, added another miRNA to the targets of GAS5; miR-196a-5p, where *GAS5* under-expression in human glioma stem cells enhanced tumor progression through inhibiting this miRNA [82]. Regarding the more controversial *miR*-*34a*, studies on brain cancer, including GB and glioma agree on its role as a tumor suppressor in this particular tissue; reviewed in [83], which fits in with its expression status in the present study.

MiR-34a expression levels in our study were not significant in the case of patients with HCC. *GAS5* levels, however, were under-expressed in HCC patients. In addition, lower GAS5 levels were marginally associated with more numerous tumor foci. The same conclusions were reached by studies conducted by Tu et al., Chang et al. and Hu et al. who found that *GAS5* was under-expressed in HCC patients and predicted poor survival in those patients [15, 26, 84]. The latter study by Hu et al. found that under-expression of *GAS5* in HCC cell lines releases its sponging effect on oncomiR-21, which normally targets the two tumor suppressor genes *PDCD4* and *PTEN* [26]. On the contrary, according to Tao et al., *GAS5* was a proto-oncogene in HCC where they found an indel polymorphism in the promotor of *GAS5* that increased the risk of HCC in Chinese. In *vitro* analysis showed that the deletion allele of the polymorphism altered methylation of the *GAS5* promoter and was associated with higher levels of GAS5 in HCC cell lines Sk-hep-1, Bel-7404 and Huh7. Further analysis provided evidence that the resulting over-expression of *GAS5* had an anti-apoptotic effect in those cell lines [85]. This divergence observed by Tao et al. could be due to racial differences. In other words, the allele causing *GAS5* over-expression might be more common in their studied population. To further confirm this probability, we searched the 1000 genome project phase 3 databases [86] for this indel polymorphism (position 1:173868254–173868258 (AGGCA/-)). We found that the frequency of the deletion allele was very high in Asians and Chinese in particular (28–40%) as compared to other races (3–12%), showing that in other populations the effect of the polymorphism on HCC and *GAS5* expression could be negligible.

## Conclusions

In this study, we show that *GAS5* is under-expressed in three types of tumors, in addition to being associated with tumor prognosis in some types. Consequently, we believe that GAS5 could potentially be used as a prognostic marker for cancer. Furthermore, we suppose that miR-34a might be a potential target of GAS5, or *vice versa*. However, further studies are required to validate this interaction.

## Supporting information

S1 Table GAS5 alternative splicing transcripts. (PDF)

S2 Table GAS5 common variants. (PDF)

S3 Table LncRNA GAS5-microRNA interaction. (PDF)

S4 Table Complementarity between GAS5 transcripts and miR-34a-5p. (PDF)

S5 Table hsa-miR-34a pathways. (PDF)

S1 Fig Receiver Operating Characteristics (ROC) analysis for the prognostic value of the markers. RCC renal cell carcinoma, GB glioblastoma, HCC hepatocellular carcinoma, AUC area under curve, CTP Child-Turcotte-Pugh classification for liver cell failure.
(TIF)

## References

[1] WHO | Cancer [WWW Document], World Health Organization. Available from URL http://www.who.int/mediacentre/factsheets/fs297/en/ (accessed 9.2.18).

[2] Grandér D. How do mutated oncogenes and tumor suppressor genes cause cancer? Med Oncol. 1998;15:20–6. 10.1007/BF02787340 9643526

[3] ENCODE Project Consortium. An integrated encyclopedia of DNA elements in the human genome. Nature. 2012;489:57–74. 10.1038/nature11247 22955616 PMC3439153

[4] Paraskevopoulou MD, Hatzigeorgiou AG. Analyzing MiRNA–LncRNA Interactions, in: Long Non-Coding RNAs, Methods in Molecular Biology. Humana Press, New York, NY, 2016; pp. 271–286. 10.1007/978-1-4939-3378-5_21 26721498

[5] Bartonicek N, Maag JLV, Dinger ME. Long noncoding RNAs in cancer: mechanisms of action and technological advancements. Mol Cancer. 2016; 15:43 10.1186/s12943-016-0530-6 27233618 PMC4884374

[6] Leva GD, Garofalo M, Croce CM. MicroRNAs in Cancer. Annu Rev Pathol Mech Dis. 2014;9:287–314. 10.1146/annurev-pathol-012513-104715 PMC4009396 24079833

[7] Mannoor K, Liao J, Jiang F. Small nucleolar RNAs in cancer. Biochim Biophys Acta. 2012;1826(1):121–128. 10.1016/j.bbcan.2012.03.005 22498252 PMC3842010

[8] Ng KW, Anderson C, Marshall EA, Minatel BC, Enfield KSS, Saprunoff HL, et al Piwi-interacting RNAs in cancer: emerging functions and clinical utility. Mol Cancer. 2016;15:5 10.1186/s12943-016-0491-9 26768585 PMC4714483

[9] Lo P-K, Wolfson B, Zhou X, Duru N, Gernapudi R, Zhou Q. Noncoding RNAs in breast cancer. Brief Funct Genomics. 2016;15:200–221. 10.1093/bfgp/elv055 26685283 PMC4880006

[10] Raho G, Barone V, Rossi D, Philipson L, Sorrentino V. The gas 5 gene shows four alternative splicing patterns without coding for a protein. Gene. 2000;256:13–17. 10.1016/S0378-1119(00)00363-2 11054530

[11] Smith CM, Steitz JA. Classification of gas5 as a Multi-Small-Nucleolar-RNA (snoRNA) Host Gene and a Member of the 5′-Terminal Oligopyrimidine Gene Family Reveals Common Features of snoRNA Host Genes. Mol Cell Biol. 1998;18:6897–6909. 9819378 10.1128/mcb.18.12.6897 PMC109273

[12] Maden BE, Hughes JM. Eukaryotic ribosomal RNA: the recent excitement in the nucleotide modification problem. Chromosoma. 1997;105:391–400. 9211966 10.1007/BF02510475

[13] Schneider C, King RM, Philipson L. Genes specifically expressed at growth arrest of mammalian cells. Cell. 1988;54:787–793. 3409319 10.1016/s0092-8674(88)91065-3

[14] Coccia EM, Cicala C, Charlesworth A, Ciccarelli C, Rossi G B, Philipson L, et al Regulation and expression of a growth arrest-specific gene (gas5) during growth, differentiation, and development. Mol Cell Biol. 1992;12:3514–3521. 1630459 10.1128/mcb.12.8.3514-3521.1992 PMC364604

[15] Chang L, Li C, Lan T, Long Wu, Yufeng Yuan, Quanyan Liuet, et al Decreased expression of long non-coding RNA GAS5 indicates a poor prognosis and promotes cell proliferation and invasion in hepatocellular carcinoma by regulating vimentin. Mol Med Rep. 2016;13:1541–1550. 10.3892/mmr.2015.4716 26707238 PMC4732840

[16] Zhao X, Wang P, Liu J, Zheng J, Liu Y, Chen J, et al Gas5 Exerts Tumor-suppressive Functions in Human Glioma Cells by Targeting miR-222. Mol Ther. 2015;23:1899–1911. 10.1038/mt.2015.170 26370254 PMC4700110

[17] Liang W, Lv T, Shi X, Liu H, Zhu Q, Zeng J, et al Circulating long noncoding RNA GAS5 is a novel biomarker for the diagnosis of nonsmall cell lung cancer. Medicine (Baltimore). 2016;95:e4608 10.1097/MD.0000000000004608 27631209 PMC5402552

[18] Cao Q, Wang N, Qi J, Gu Z, Shen H. Long non‑coding RNA‑GAS5 acts as a tumor suppressor in bladder transitional cell carcinoma via regulation of chemokine (C‑C motif) ligand 1 expression. Mol Med Rep. 2016;13:27–34. 10.3892/mmr.2015.4503 26548923 PMC4686088

[19] Yacqub-Usman K, Pickard MR, Williams GT. Reciprocal regulation of GAS5 lncRNA levels and mTOR inhibitor action in prostate cancer cells. The Prostate 2015;75:693–705. 10.1002/pros.22952 25650269

[20] Pickard MR, Williams GT. Regulation of apoptosis by long non-coding RNA GAS5 in breast cancer cells: implications for chemotherapy. Breast Cancer Res Treat. 2014;145:359–3570. 10.1007/s10549-014-2974-y 24789445

[21] Guo C, Song W-Q, Sun P, Jin L, Dai H-Y. LncRNA-GAS5 induces PTEN expression through inhibiting miR-103 in endometrial cancer cells. J. Biomed. Sci. 2015;22:100 10.1186/s12929-015-0213-4 26511107 PMC4625733

[22] Lu X, Fang Y, Wang Z, Xie J, Zhan Q, Deng X, et al Downregulation of gas5 increases pancreatic cancer cell proliferation by regulating CDK6. Cell Tissue Res. 2013;354:891–896. 10.1007/s00441-013-1711-x 24026436

[23] Yin D, He X, Zhang E, Kong R, De W, Zhang Z. Long noncoding RNA GAS5 affects cell proliferation and predicts a poor prognosis in patients with colorectal cancer. Med Oncol Northwood Lond Engl. 2014;31:253 10.1007/s12032-014-0253-8 25326054

[24] Qiao H-P, Gao W-S, Huo J-X, Yang Z-S. Long Non-coding RNA GAS5 Functions as a Tumor Suppressor in Renal Cell Carcinoma. Asian Pac J Cancer Prev. 2013;14:1077–1082. 10.7314/APJCP.2013.14.2.1077 23621190

[25] Nakamura Y, Takahashi N, Kakegawa E, Yoshida K, Ito Y, Kayano H, et al The GAS5 (growth arrest-specific transcript 5) gene fuses to BCL6 as a result of t(1;3) (q25;q27) in a patient with B-cell lymphoma. Cancer Genet. Cytogenet. 2008;182:144–149. 10.1016/j.cancergencyto.2008.01.013 18406879

[26] Hu L, Ye H, Huang G, Luo F, Liu Y, Liu Y, et al Long noncoding RNA GAS5 suppresses the migration and invasion of hepatocellular carcinoma cells via miR-21. Tumour Biol. 2016;37:2691–2702. 10.1007/s13277-015-4111-x 26404135

[27] Zhang Z, Zhu Z, Watabe K, Zhang X, Bai C, Xu M, et al Negative regulation of lncRNA GAS5 by miR-21. Cell Death Differ. 2013;20:1558–1568. 10.1038/cdd.2013.110 23933812 PMC3792431

[28] He L, He X, Lim LP, de Stanchina E, Xuan Z, Liang Y, et al A microRNA component of the p53 tumour suppressor network. Nature. 2007;447:1130–1134. 10.1038/nature05939 17554337 PMC4590999

[29] Lodygin D, Tarasov V, Epanchintsev A, Berking C, Knyazeva T, Körner H, et al Inactivation of miR-34a by aberrant CpG methylation in multiple types of cancer. Cell Cycle. 2008;7:2591–2600. 10.4161/cc.7.16.6533 18719384

[30] Welch C, Chen Y, Stallings RL. MicroRNA-34a functions as a potential tumor suppressor by inducing apoptosis in neuroblastoma cells. Oncogene. 2007;26:5017–5022. 10.1038/sj.onc.1210293 17297439

[31] Chim CS, Wong KY, Qi Y, Loong F, Lam WL, Wong LG, et al Epigenetic inactivation of the miR-34a in hematological malignancies. Carcinogenesis. 2010;31:745–750. 10.1093/carcin/bgq033 20118199

[32] Ji Q, Hao X, Zhang M, Tang W, Yang M, Li L, et al MicroRNA miR-34 inhibits human pancreatic cancer tumor-initiating cells. PloS One. 2009;4:e6816 10.1371/journal.pone.0006816 19714243 PMC2729376

[33] Li N, Fu H, Tie Y, Hu Z, Kong W, Wu Y, et al miR-34a inhibits migration and invasion by down-regulation of c-Met expression in human hepatocellular carcinoma cells. Cancer Lett. 2009;275:44–53. 10.1016/j.canlet.2008.09.035 19006648

[34] Shehata RH, Abdelmoneim SS, Osman OA, Hasanain AF, Osama A, Abdelmoneim SS, et al Deregulation of miR-34a and Its Chaperon Hsp70 in Hepatitis C virus-Induced Liver Cirrhosis and Hepatocellular Carcinoma Patients. Asian Pacific Journal of Cancer Prevention. APJCP. 2017;18(9):2395–2401. doi: 10.22034/APJCP.2017.18.9.2395 28950684 10.22034/APJCP.2017.18.9.2395 PMC5720642

[35] Li Y, Guessous F, Zhang Y, Dipierro C, Kefas B, Johnson E, et al MicroRNA-34a inhibits glioblastoma growth by targeting multiple oncogenes. Cancer Res. 2009;69:7569–76. 10.1158/0008-5472.CAN-09-0529 19773441 PMC2756313

[36] Toraih EA, Aly NM, Abdallah HY, Al-Qahtani SA, Shaalan AA, Hussein MH, et al MicroRNA-target cross-talks: Key players in glioblastoma multiforme. Tumour Biol. 2017;39(11):1010428317726842. 10.1177/1010428317726842 29110584

[37] Kato M1, Paranjape T, Müller RU, Nallur S, Gillespie E, Keane K, et al The mir-34 microRNA is required for the DNA damage response in vivo in C. elegans and in vitro in human breast cancer cells. Oncogene. 2009;28:2419–2424. 10.1038/onc.2009.106 19421141 PMC2941141

[38] Wiggins JF, Ruffino L, Kelnar K, Omotola M, Patrawala L, Brown D, et al Development of a lung cancer therapeutic based on the tumor suppressor microRNA-34. Cancer Res. 2010;70:5923–5930. 10.1158/0008-5472.CAN-10-0655 20570894 PMC2913706

[39] Tazawa H, Tsuchiya N, Izumiya M, Nakagama H. Tumor-suppressive miR-34a induces senescence-like growth arrest through modulation of the E2F pathway in human colon cancer cells. Proc Natl Acad Sci USA. 2007;104:15472–15477. 10.1073/pnas.0707351104 17875987 PMC2000550

[40] Liu H, Brannon AR, Reddy AR, Alexe G, Seiler MW, Arreola A, et al Identifying mRNA targets of microRNA dysregulated in cancer: with application to clear cell Renal Cell Carcinoma. BMC Syst Biol. 2010;4:51 10.1186/1752-0509-4-51 20420713 PMC2876063

[41] Toraih EA, Ibrahiem AT, Fawzy MS, Hussein MH, Al-Qahtani SAM, Shaalan AAM. MicroRNA-34a: A Key Regulator in the Hallmarks of Renal Cell Carcinoma. Oxid Med Cell Longev. 2017;2017:3269379 10.1155/2017/3269379 29104726 PMC5632457

[42] Ma Y, Qin H, Cui Y. MiR-34a targets GAS1 to promote cell proliferation and inhibit apoptosis in papillary thyroid carcinoma via PI3K/Akt/Bad pathway. Biochem Biophys Res Commun. 2013;441:958–963. 10.1016/j.bbrc.2013.11.010 24220341

[43] Marsh EE, Lin Z, Yin P, Milad M, Chakravarti D, Bulun SE. Differential expression of microRNA species in human uterine leiomyoma versus normal myometrium. Fertil Steril. 2008;89:1771–1776. 10.1016/j.fertnstert.2007.05.074 17765232 PMC2773156

[44] Llovet JM, Fuster J, Bruix J. Barcelona-Clínic Liver Cancer Group. The Barcelona approach: diagnosis, staging, and treatment of hepatocellular carcinoma. Liver Transpl. 2004;10:S115–120. 10.1002/lt.20034 14762851

[45] Toraih EA, Fawzy MS, El-Falouji AI, Hamed EO, Nemr NA, Hussein MH, et al Stemness-Related Transcriptional Factors and Homing Gene Expression Profiles in Hepatic Differentiation and Cancer. Mol Med. 2016;22:653–663. 10.2119/molmed.2016.00096 27623812 PMC5082293

[46] Toraih EA, Fawzy MS, Mohammed EA, Hussein MH, El-Labban MM. MicroRNA-196a2 Biomarker and Targetome Network Analysis in Solid Tumors. Mol Diagn Ther. 2016;20:559–577. 10.1007/s40291-016-0223-2 27342110

[47] Bustin SA, Benes V, Garson JA, Hellemans J, Huggett J, Kubista M, et al The MIQE guidelines: minimum information for publication of quantitative real-time PCR experiments. Clin Chem. 2009;55:611–622. 10.1373/clinchem.2008.112797 19246619

[48] Toraih EA, Mohammed EA, Farrag S, Ramsis N, Hosny S. Pilot Study of Serum MicroRNA-21 as a Diagnostic and Prognostic Biomarker in Egyptian Breast Cancer Patients. Mol Diagn Ther. 2015;19:179–190. 10.1007/s40291-015-0143-6 26063582

[49] Valente V, Teixeira SA, Neder L, Okamoto OK, Oba-Shinjo SM, Marie SK, et al Selection of suitable housekeeping genes for expression analysis in glioblastoma using quantitative RT-PCR. Ann Neurosci. 2014;21(2):62–63. 10.5214/ans.0972.7531.210207 25206063 PMC4117159

[50] Aithal MGS, Rajeswari N. Validation of Housekeeping Genes for Gene Expression Analysis in Glioblastoma Using Quantitative Real-Time Polymerase Chain Reaction. Brain Tumor Res Treat. 2015;3:24–29. doi: 10.14791/btrt.2015.3.1.24 25977903 10.14791/btrt.2015.3.1.24 PMC4426273

[51] Livak KJ, Schmittgen TD. Analysis of relative gene expression data using real-time quantitative PCR and the 2 (-Delta Delta C (T)) Method. Methods. 2001,25:402–408. 10.1006/meth.2001.1262 11846609

[52] McCune B, Mefford MJ. PC-ORD. Multivariate Analysis of Ecological Data. Version 6. MjM Software, Gleneden Beach, Oregon, USA, 2011.

[53] Yamashita R, Suzuki Y, Takeuchi N, Wakaguri H, Ueda T, Sugano S, et al Comprehensive detection of human terminal oligo-pyrimidine (TOP) genes and analysis of their characteristics. Nucleic Acids Res. 2008;36:3707–3715. 10.1093/nar/gkn248 18480124 PMC2441802

[54] Cheng T, Wang L, Li Y, Huang C, Zeng L, Yang J. Differential microRNA expression in renal cell carcinoma. Oncol Lett. 2013;6:769–776. 10.3892/ol.2013.1460 24137408 PMC3788858

[55] Munari E, Marchionni L, Chitre A, Hayashi M, Martignoni G, Brunelli M, et al Clear cell papillary renal cell carcinoma: micro-RNA expression profiling and comparison with clear cell renal cell carcinoma and papillary renal cell carcinoma. Hum Pathol. 2014;45:1130–1138. 10.1016/j.humpath.2014.01.013 24703100 PMC4332813

[56] Fritz HK, Gustafsson A, Ljungberg B, Ceder Y, Axelson H, Dahlbäck B. The Axl-Regulating Tumor Suppressor miR-34a Is Increased in ccRCC but Does Not Correlate with Axl mRNA or Axl Protein Levels. PloS One. 2015;10:e0135991 10.1371/journal.pone.0135991 26287733 PMC4546115

[57] Bhatt K, Zhou L, Mi QS, Huang S, She JX, Dong Z. MicroRNA-34a is induced via p53 during cisplatin nephrotoxicity and contributes to cell survival. Mol Med. 2010;16:409–416. 10.2119/molmed.2010.00002 20386864 PMC2935954

[58] Yadav S, Khandelwal M, Seth A, Saini AK, Dogra PN, Sharma A. Serum microRNA Expression Profiling: Potential Diagnostic Implications of a Panel of Serum microRNAs for Clear Cell Renal Cell Cancer. Urology. 2017;104:64–69. 10.1016/j.urology.2017.03.013 28336290

[59] Zhang EB, Kong R, Yin DD, You LH, Sun M, Han L, et al Long noncoding RNA ANRIL indicates a poor prognosis of gastric cancer and promotes tumor growth by epigenetically silencing of miR-99a/miR-449a. Oncotarget. 2014;5:2276–2292. doi: 10.18632/oncotarget.1902 24810364 10.18632/oncotarget.1902 PMC4039162

[60] Weng W, Wang M, Xie S, Long Y, Li F, Sun F, et al YY1-C/EBPα-miR34a regulatory circuitry is involved in renal cell carcinoma progression. Oncol Rep. 2014;31:1921–1927. 10.3892/or.2014.3005 24481728

[61] Yu G, Li H, Wang J, Gumireddy K, Li A, Yao W, et al miRNA-34a Suppresses Cell Proliferation and Metastasis by Targeting CD44 in Human Renal Carcinoma Cells. J Urol. 2014;192:1229–1237. 10.1016/j.juro.2014.05.094 24866595

[62] Yamamura S, Saini S, Majid S, Hirata H, Ueno K, Chang I, et al MicroRNA-34a suppresses malignant transformation by targeting c-Myc transcriptional complexes in human renal cell carcinoma. Carcinogenesis. 2012;33:294–300. 10.1093/carcin/bgr286 22159222 PMC3271269

[63] Liu F, Chen N, Gong Y, Xiao R, Wang W, Pan Z. The long non-coding RNA NEAT1 enhances epithelial-to-mesenchymal transition and chemoresistance via the miR-34a/c-Met axis in renal cell carcinoma. Oncotarget. 2017;8:62927–62938. doi: 10.18632/oncotarget.17757 28968960 10.18632/oncotarget.17757 PMC5609892

[64] Bai XY, Ma Y, Ding R, Fu B, Shi S, Chen XM. miR-335 and miR-34a Promote Renal Senescence by Suppressing Mitochondrial Antioxidative Enzymes. J Am Soc Nephrol. 2011;22:1252–1261. 10.1681/ASN.2010040367 21719785 PMC3137573

[65] Collado M, Blasco MA, Serrano M. Cellular Senescence in Cancer and Aging. Cell. 2007;130:223–233. 10.1016/j.cell.2007.07.003 17662938

[66] Zhou Y, Xiong M, Niu J, Sun Q, Su W, Zen K, et al Secreted fibroblast-derived miR-34a induces tubular cell apoptosis in fibrotic kidney. J Cell Sci. 2014;127:4494–4506. 10.1242/jcs.155523 25107369

[67] Borrell B. How accurate are cancer cell lines? Nature. 2010;463:858 10.1038/463858a 20164888

[68] Clark MJ, Homer N, O'Connor BD, Chen Z, Eskin A, Lee H, et al U87MG Decoded: The Genomic Sequence of a Cytogenetically Aberrant Human Cancer Cell Line. PLoS Genet. 2010;6:e1000832 10.1371/journal.pgen.1000832 20126413 PMC2813426

[69] Yoon JH, Abdelmohsen K, Srikantan S, Yang X, Martindale JL, De S, et al LincRNA-p21 suppresses target mRNA translation. Mol Cell. 2012;47:648–655. 10.1016/j.molcel.2012.06.027 22841487 PMC3509343

[70] Wang Y, Xu Z, Jiang J, Xu C, Kang J, Xiao L, et al Endogenous miRNA Sponge lincRNA-RoR Regulates Oct4, Nanog, and Sox2 in Human Embryonic Stem Cell Self-Renewal. Dev Cell. 2013;25:69–80. 10.1016/j.devcel.2013.03.002 23541921

[71] Leucci E, Patella F, Waage J, Holmstrøm K, Lindow M, Porse B, et al microRNA-9 targets the long non-coding RNA MALAT1 for degradation in the nucleus. Sci Rep. 2013;3:2535 10.1038/srep02535 23985560 PMC3756333

[72] Faghihi MA, Zhang M, Huang J, Modarresi F, Van der Brug MP, Nalls MA, et al Evidence for natural antisense transcript-mediated inhibition of microRNA function. Genome Biol. 2010;11:R56 10.1186/gb-2010-11-5-r56 20507594 PMC2898074

[73] Keniry A, Oxley D, Monnier P, Kyba M, Dandolo L, Smits G, et al The H19 lincRNA is a developmental reservoir of miR-675 that suppresses growth and Igf1r. Nat Cell Biol. 2012;14:659–665. 10.1038/ncb2521 22684254 PMC3389517

[74] Guo C, Song WQ, Sun P, Jin L, Dai HY. LncRNA-GAS5 induces PTEN expression through inhibiting miR-103 in endometrial cancer cells. J Biomed Sci. 2015;22:100 10.1186/s12929-015-0213-4 26511107 PMC4625733

[75] Xue D, Zhou C, Lu H, Xu R, Xu X, He X. LncRNA GAS5 inhibits proliferation and progression of prostate cancer by targeting miR-103 through AKT/mTOR signaling pathway. Tumor Biol. 2016;37:16187–16197. 10.1007/s13277-016-5429-8 27743383

[76] Yang W, Hong L, Xu X, Wang Q, Huang J, Jiang L. LncRNA GAS5 suppresses the tumorigenesis of cervical cancer by downregulating miR-196a and miR-205. Tumour Biol. 2017;39:1010428317711315 10.1177/1010428317711315 28671039

[77] Ye K, Wang S, Zhang H, Han H, Ma B, Nan W. Long Noncoding RNA GAS5 Suppresses Cell Growth and Epithelial-Mesenchymal Transition in Osteosarcoma by Regulating the miR-221/ARHI Pathway. J Cell Biochem. 2017;118:4772–4781. 10.1002/jcb.26145 28519068

[78] Yu F, Zheng J, Mao Y, Dong P, Lu Z, Li G, et al Long Non-coding RNA Growth Arrest-specific Transcript 5 (GAS5) Inhibits Liver Fibrogenesis through a Mechanism of Competing Endogenous RNA. J Biol Chem. 2015; 290:28286–28298. 10.1074/jbc.M115.683813 26446789 PMC4653684

[79] Brameier M, Herwig A, Reinhardt R, Walter L, Gruber J. Human box C/D snoRNAs with miRNA like functions: expanding the range of regulatory RNAs. Nucleic Acids Res. 2011;39:675–686. 10.1093/nar/gkq776 20846955 PMC3025573

[80] Domínguez-Monzón G, Benítez JA, Vergara P, Lorenzana R, Segovia J. Gas1 inhibits cell proliferation and induces apoptosis of human primary gliomas in the absence of Shh. Int J Dev Neurosci. 2009;27:305–313. 10.1016/j.ijdevneu.2009.03.009 19460624

[81] Zhang XQ, Sun S, Lam KF, Kiang KM, Pu JK, Ho AS, et al A long non-coding RNA signature in glioblastoma multiforme predicts survival. Neurobiol Dis. 2013;58:123–131. 10.1016/j.nbd.2013.05.011 23726844

[82] Zhao X, Liu Y, Zheng J, Liu X, Chen J, Liu L, et al GAS5 suppresses malignancy of human glioma stem cells via a miR-196a-5p/FOXO1 feedback loop. Biochim Biophys Acta. 2017;1864:1605–1617. 10.1016/j.bbamcr.2017.06.020 28666797

[83] Møller HG, Rasmussen AP, Andersen HH, Johnsen KB, Henriksen M, Duroux M. A Systematic Review of MicroRNA in Glioblastoma Multiforme: Micro-modulators in the Mesenchymal Mode of Migration and Invasion. Mol Neurobiol. 2013;47:131–144. 10.1007/s12035-012-8349-7 23054677 PMC3538124

[84] Tu ZQ, Li RJ, Mei JZ, Li XH. Down-regulation of long non-coding RNA GAS5 is associated with the prognosis of hepatocellular carcinoma. Int. J Clin Exp Pathol. 2014;7:4303–4309. 25120813 PMC4129048

[85] Tao R, Hu S, Wang S, Zhou X, Zhang Q, Wang C, et al Association between indel polymorphism in the promoter region of lncRNA GAS5 and the risk of hepatocellular carcinoma. Carcinogenesis. 2015;36:1136–1143. 10.1093/carcin/bgv099 26163879

[86] The 1000 Genomes Project Consortium. A global reference for human genetic variation. Nature. 2015;526:68–74. 10.1038/nature15393 26432245 PMC4750478

