# Supplementary material for: Dual biomarkers long non-coding RNA GAS5 and microRNA-34a co-expression signature in common solid tumors
Source: PLoS One. 2018 Oct 5;13(10):e0198231. doi: 10.1371/journal.pone.0198231 (PMC6173395; doi:10.1371/journal.pone.0198231)
Supplement: S1 Table — (PDF) [file pone.0198231.s001.pdf]

S1 Table. GAS5 alternative splicing transcripts.

Name	Transcript ID	Length (bp)	Protein	Biotype	RefSeq
**GAS5-210**	ENST00000431268.5	1698	No protein	Retained intron	
**GAS5-215**	ENST00000442067.5	1114	No protein	Retained intron	
**GAS5-204**	ENST00000421068.5	1060	No protein	Retained intron	
**GAS5-201**	ENST00000412059.5	979	No protein	Retained intron	
**GAS5-216**	ENST00000443799.5	897	No protein	Retained intron	
**GAS5-214**	ENST00000436656.5	822	No protein	Retained intron	
**GAS5-203**	ENST00000416952.5	799	No protein	Retained intron	
**GAS5-213**	ENST00000436285.5	772	No protein	Retained intron	
**GAS5-206**	ENST00000422183.5	745	No protein	Retained intron	
**GAS5-228**	ENST00000456812.6	723	No protein	Retained intron	
**GAS5-220**	ENST00000449589.5	712	No protein	Retained intron	
**GAS5-207**	ENST00000422207.5	643	No protein	Retained intron	
**GAS5-226**	ENST00000455838.5	632	No protein	Retained intron	
**GAS5-225**	ENST00000454813.1	621	No protein	Retained intron	
**GAS5-227**	ENST00000456293.5	583	No protein	Retained intron	
**GAS5-212**	ENST00000434796.5	575	No protein	Retained intron	
**GAS5-205**	ENST00000422008.5	497	No protein	Retained intron	
**GAS5-229**	ENST00000458220.1	469	No protein	Retained intron	
**GAS5-217**	ENST00000444470.5	424	No protein	Retained intron	
**GAS5-222**	ENST00000451607.5	1007	No protein	lincRNA	
**GAS5-211**	ENST00000432536.5	959	No protein	lincRNA	
**GAS5-209**	ENST00000430245.5	723	No protein	lincRNA	
**GAS5-224**	ENST00000454068.5	688	No protein	lincRNA	
**GAS5-221**	ENST00000450589.5	632	No protein	lincRNA	NR_002578
**GAS5-218**	ENST00000448718.5	565	No protein	lincRNA	
**GAS5-219**	ENST00000449289.5	542	No protein	lincRNA	
**GAS5-223**	ENST00000452197.5	483	No protein	lincRNA	
**GAS5-202**	ENST00000414075.5	413	No protein	lincRNA	
**GAS5-208**	ENST00000425771.5	242	No protein	lincRNA	

http://www.rnacentral.org/rna/URS00009C227F/9606
