# Supplementary material for: Dual biomarkers long non-coding RNA GAS5 and microRNA-34a co-expression signature in common solid tumors
Source: PLoS One. 2018 Oct 5;13(10):e0198231. doi: 10.1371/journal.pone.0198231 (PMC6173395; doi:10.1371/journal.pone.0198231)
Supplement: S2 Table — (PDF) [file pone.0198231.s002.pdf]

S2 Table. GAS5 common variants.

Variant ID	Chr: bp	Alleles	Global MAF	Minor allel	Global MAF
rs74609518	1:173865156	C/T	0.065	T	SNP
rs9442660	1:173865218	A/G	0.112	G	SNP
rs2235095	1:173865467	G/A	0.12	A	SNP
rs6790	1:173865494	G/A	0.12	A	SNP
rs14262	1:173865545	G/A	0.065	A	SNP
rs2067079	1:173866073	C/T	0.193	T	SNP
rs9425427	1:173866363	T/C	0.094	C	SNP
rs1322772	1:173866620	C/A	0.112	A	SNP
rs13476	1:173866781	T/A	0.094	A	SNP
rs17359906	1:173867056	G/A	0.062	A	SNP
rs144869587	1:173867109 - 173867110	-/A	0.192	A	insertion
rs9660881	1:173867378	T/C	0.207	C	SNP
rs1951625	1:173867464	G/A	0.336	A	SNP
rs75315904	1:173867913	G/A	0.12	A	SNP
rs55829688	1:173868168	T/C	0.207	C	SNP
rs145204276	1:173868254 -173868258	AGGCA/-	0.12	-	deletion

Conseq. Type	Transcript
intron variant	ENST00000412059.5
intron variant	ENST00000412059.5
intron variant	ENST00000456812.6
non coding transcript exon variant	ENST00000412059.5
splice region variant	ENST00000456812.6
intron variant	ENST00000412059.5
non coding transcript exon variant	ENST00000458220.1
intron variant	ENST00000412059.5
non coding transcript exon variant	ENST00000458220.1
intron variant	ENST00000412059.5
intron variant	ENST00000412059.5
intron variant	ENST00000458220.1
intron variant	ENST00000412059.5
intron variant	ENST00000458220.1
intron variant	ENST00000430245.5
intron variant	ENST00000430245.5
