# Supplementary material for: Dual biomarkers long non-coding RNA GAS5 and microRNA-34a co-expression signature in common solid tumors
Source: PLoS One. 2018 Oct 5;13(10):e0198231. doi: 10.1371/journal.pone.0198231 (PMC6173395; doi:10.1371/journal.pone.0198231)
Supplement: S3 Table — (PDF) [file pone.0198231.s003.pdf]

S3 Table. LncRNA GAS5-microRNA interaction.

most pred	miRNA	Folding en	GAS5 target site sequence
166	hsa_miR_3150a_3	12.1	TAAACTTTGAAGGAAATTTTTAA
11	hsa_miR_103a_2_5p	12.4	TTGGGTAAGGACATGAAGACAGTT
49	hsa_miR_128_1_5	12.4	GTAAGGACATGAAGACAGTTCCT
67	hsa_miR_138_2_3	12.4	AAAGCTGGAAGTTGAAATGGT
67	hsa_miR_128_1_5	12.4	TTTGAAGCTTACTACAGCCTCA
433	hsa_miR_548au_3	13	ATGATGTAATGATTCTGCCA
40	hsa_miR_125b_1_	13.4	AGGTATGGAGAGTCGGCTTGA
205	hsa_miR_3622a_5	14.6	CAAGCATGCAGCTTACTGCTTG
556	hsa_miR_6780a_3	14.2	CTGGAAGTTGAAATGGTGGAG
301	hsa_miR_3689a_3	14.3	ACCAATGATGTAATGATTCTGCCAA
86	hsa_miR_1827	-12.9	AAAGGGTCTTGCCTCA
305	hsa_miR_450a_2_	14.6	AGATTAAAACAGTGTCTCCAAT
679	hsa_miR_92a_2_5	14.8	TGGGCTCAAGTGATCCTCAGCC
266	hsa_miR_4316	-16.9	AAAGGGTCTTGCCTCACC
558	hsa_miR_6780b_5	15.2	ACTTCTGGGCTCAAGTGATCCTCA
121	hsa_miR_218_2_3	15.4	AGTCGGCTTGACTACACTGTG
46	hsa_miR_1273g_5	17.6	GCTCAAGTGATCCTCAGCCTCC
207	hsa_miR_3622b_5	18.3	TCAAGTGATCCTCAGCCT
557	hsa_miR_6780a_5	19.9	GCTCAAGTGATCCTCAGCCTCCCAG
225	hsa_miR_3689a_3	20	GCTCAAGTGATCCTCAGCCTCCCAG
501	hsa_miR_6511a_5	20.5	AGAGTCGGCTTGACTACACTGTGTG
226	hsa_miR_3689b_3	20.8	GCTCAAGTGATCCTCAGCCTCCCAG
47	hsa_miR_1273h_5	21.3	AGTGATCCTCAGCCTCCCAG
279	hsa_miR_4443	-14.1	AAAACAGTGTCTCCAA
377	hsa_miR_4472	-13.8	AAAACAGTGTCTCCAATT
496	hsa_miR_6505_5p	-12.6	AAAACAGTGTCTCCAATTTTAA
664	hsa_miR_8077	-16	AAAGAGCAAGCCTAACTCAAGCC
497	hsa_miR_6506_3p	-12	AAAGCTGGAAGTTGAAATGG
416	hsa_miR_5187_5p	-13.3	AAAGGGTCTTGCCTCACCCA
124	hsa_miR_224_3p	-12.2	AAATTTTAAATGGGTATTATTTT
354	hsa_miR_4715_5p	-14.3	AAGCAAGCATGCAGCTTACTG
100	hsa_miR_194_3p	-16.1	AAGCAAGCATGCAGCTTACTGC
94	hsa_miR_1909_3p	-16.3	AAGCAAGCATGCAGCTTACTGCT
434	hsa_miR_548ax	-12.8	AAGCAAGCATGCAGCTTACTGCT
36	hsa_miR_1244	-16.3	AAGCAAGCATGCAGCTTACTGCTT
244	hsa_miR_3944_3p	-15.3	AAAGGTATGGAGAGTCGGCTTGAC
105	hsa_miR_197_5p	-13.3	AAGCAAGCATGCAGCTTACTGCTTG
633	hsa_miR_7112_5p	-14.3	AAGCAAGCATGCAGCTTACTGCTTGA
97	hsa_miR_1910_3p	-13.8	AAGCATGCAGCTTACTGCTTG
276	hsa_miR_4436a	-12.1	AACCAATGATGTAATGATTCTGC
311	hsa_miR_4525	-14.2	AACCAATGATGTAATGATTCTGCC
93	hsa_miR_1908_5p	-13.9	AACCAATGATGTAATGATTCTGCCA
41	hsa_miR_126_3p	-12.4	AACGTTTTTATCCATGGATGA

608	hsa_miR_6862_5p	-12.4	AACGTTTTTATCCATGGATGACTTG
95	hsa_miR_1909_5p	-14.7	AACTCAAGCCATTGGCACACA
589	hsa_miR_6825_5p	-12	AAGAAGATTAAAACAGTGTCTCCA
202	hsa_miR_3122	-12.94	AAGATTAAAACAGTGTCTCCAAT
454	hsa_miR_585_3p	-17.3	AAGCATGCAGCTTACTGCTTG
263	hsa_miR_1244	-14.6	ACAACAAGCAAGCATGCAGCTT
395	hsa_miR_497_5p	-12.4	ACAAGCAAGCATGCAGCTT
513	hsa_miR_6726_5p	-15.5	ACAAGCAAGCATGCAGCTTACTG
406	hsa_miR_5047	-16.5	ACCACAACAAGCAAGCATGCAG
518	hsa_miR_6736_5p	-16.2	ACCACAACAAGCAAGCATGCAG
50	hsa_miR_1286	-16.7	ACTGCTTGAAAGGGTCTTGCC
611	hsa_miR_6869_5p	-15.1	ACTGCTTGAAAGGGTCTTGCCTCAC
176	hsa_miR_3174	-12.6	AGCAAGCATGCAGCTTACTG
131	hsa_miR_2467_3p	-13.2	AGCAAGCATGCAGCTTACTGCT
670	hsa_miR_885_3p	-15.6	AGCAAGCATGCAGCTTACTGCTT
577	hsa_miR_6811_3p	-14.6	AAGCCATTGGCACACAGGCA
535	hsa_miR_5007_5p	-14.5	AAGCCATTGGCACACAGGCATTA
915	hsa_miR_8069	-13.9	AAGCCATTGGCACACAGGCATTA
662	hsa_miR_8071	-16.5	AAGCCTAACTCAAGCCATTG
420	hsa_miR_5190	-14.8	AAGCCTAACTCAAGCCATTGG
160	hsa_miR_3127_5p	-14.1	AAGCCTAACTCAAGCCATTGGC
648	hsa_miR_616_3p	-13.1	AAGCCTAACTCAAGCCATTGGCA
415	hsa_miR_4537	-15.4	AAGCCTAACTCAAGCCATTGGCACA
108	hsa_miR_199a_5p	-12.4	AAGCTGGAAGTTGAAATGGTGGA
565	hsa_miR_6788_3p	-14.2	AAGCTGGAAGTTGAAATGGTGGA
208	hsa_miR_3130_3p	-14.5	AAGCTGGAAGTTGAAATGGTGGAGT
140	hsa_miR_2861	-16.5	AAGCTTACTACAGCCTC
260	hsa_miR_4283	-14.8	AAGCTTACTACAGCCTCA
299	hsa_miR_4500	-14.7	AAGCTTACTACAGCCTCA
241	hsa_miR_3936	-12.2	AGCAAGCATGCAGCTTACTGCTTG
376	hsa_miR_4756_5p	-13.4	AGCAAGCATGCAGCTTACTGCTTG
512	hsa_miR_6721_5p	-12.8	AGCAAGCATGCAGCTTACTGCTTG
405	hsa_miR_504_5p	-15.1	AGCTTACTGCTTGAAAGGGTCT
651	hsa_miR_769_5p	-17.3	AGCTTACTGCTTGAAAGGGTCTTG
255	hsa_miR_4260	-16.1	AGGGTCTTGCCTCACCCAAG
218	hsa_miR_3151_5p	-18.9	AAGTGATCCTCAGCCTCCC
449	hsa_miR_5698	-14.4	AAGTGATCCTCAGCCTCCCA
156	hsa_miR_3122	-13.7	AAGTGATCCTCAGCCTCCCAGT
543	hsa_miR_6761_5p	-12.3	AAGTGATCCTCAGCCTCCCAGT
836	hsa_miR_6861_5p	-21.1	AAGTGATCCTCAGCCTCCCAGT
159	hsa_miR_2467_5p	-14.5	AAGTTGAAATGGTGGAGTCCA
249	hsa_miR_423_3p	-15.8	AGGGTCTTGCCTCACCCAAGCT
363	hsa_miR_4730	-13.9	AATGATGTAATGATTCTGCCAA
315	hsa_miR_4538	-21	AGGGTCTTGCCTCACCCAAGCTA
596	hsa_miR_6839_5p	-15.3	AGGGTCTTGCCTCACCCAAGCTAGA
88	hsa_miR_185_5p	-12.5	ATGGGTATTATTTTTCATCTTCA
534	hsa_miR_6751_5p	-15	ATGGGTATTATTTTTCATCTTCAA

472	hsa_miR_6125	-12.7	CAAGCATGCAGCTTACTGC
542	hsa_miR_6760_5p	-13.1	CAAGCATGCAGCTTACTGCTTG
500	hsa_miR_6509_5p	-12.1	CAAGCATGCAGCTTACTGCTTGAA
190	hsa_miR_339_5p	-14.3	CATGCAGCTTACTGCTTGAAAGGGT
172	hsa_miR_3157_5p	-14.2	ACACAGGCATTAGACAGAAAGCTGGA
13	hsa_miR_103b	-13.7	ACAGGCATTAGACAGAAAGCTGGA
502	hsa_miR_6512_3p	-16.5	ACAGGCATTAGACAGAAAGCTGGAA
511	hsa_miR_6720_5p	-16.7	ACAGGCATTAGACAGAAAGCTGGAA
504	hsa_miR_658	-19.5	ACCAATGATGTAATGATTCTGCC
296	hsa_miR_449a	-15.1	ACCAATGATGTAATGATTCTGCCA
517	hsa_miR_6731_5p	-12.1	ACCAATGATGTAATGATTCTGCCA
78	hsa_miR_152_3p	-12.8	CCAAGCTAGAGTGCAGTGG
456	hsa_miR_593_3p	-15.3	CCTCACCCAAGCTAGAGTGCA
605	hsa_miR_6860	-21.1	CCTCACCCAAGCTAGAGTGCAGT
333	hsa_miR_4660	-15.74	CTCACCCAAGCTAGAGTGCA
641	hsa_miR_7158_3p	-13.2	CTCACCCAAGCTAGAGTGCAG
475	hsa_miR_616_3p	-15.4	ACGTTTTTATCCATGGATGACT
455	hsa_miR_592	-13	ACTCAAGCCATTGGCACAC
268	hsa_miR_4322	-14.6	ACTCAAGCCATTGGCACACAG
38	hsa_miR_1251_3p	-12.7	CTCACCCAAGCTAGAGTGCAGTG
576	hsa_miR_6808_3p	-13.8	CTGCTTGAAAGGGTCTTGC
288	hsa_miR_4479	-16.8	CTGCTTGAAAGGGTCTTGCC
593	hsa_miR_6836_5p	-25.1	CTGCTTGAAAGGGTCTTGCC
31	hsa_miR_1207_5p	-13	CTGCTTGAAAGGGTCTTGCCT
239	hsa_miR_3927_3p	-12.4	CTGCTTGAAAGGGTCTTGCCTC
615	hsa_miR_6881_5p	-14.52	CTGCTTGAAAGGGTCTTGCCTCA
494	hsa_miR_6504_3p	-12.1	AGAAAGCTGGAAGTTGAAATG
355	hsa_miR_4716_5p	-14	AGAAAGCTGGAAGTTGAAATGGTGGA
22	hsa_miR_1184	-12.7	AGAATTCATGATTGAAGAAATGCAGG
284	hsa_miR_3650	-12.3	AGAGTCGGCTTGACTACACTG
446	hsa_miR_5683	-12.8	AGAGTCGGCTTGACTACACTGTG
222	hsa_miR_3681_3p	-15.9	AGAGTCGGCTTGACTACACTGTGT
484	hsa_miR_639	-12.9	AGAGTCGGCTTGACTACACTGTGTGGA
237	hsa_miR_3918	-17	AGATGCAGTGTGGCTCTGG
141	hsa_miR_2909	-12.8	AGATGCAGTGTGGCTCTGGA
429	hsa_miR_525_5p	-16.7	AGATGCAGTGTGGCTCTGGAT
278	hsa_miR_4440	-12.5	AGATGCAGTGTGGCTCTGGATA
655	hsa_miR_7852_3p	-14.9	AGATGCAGTGTGGCTCTGGATA
32	hsa_miR_1225_5p	-19.5	CTGCTTGAAAGGGTCTTGCCTCAC
604	hsa_miR_6858_5p	-16.1	CTGCTTGAAAGGGTCTTGCCTCAC
629	hsa_miR_711	-16.1	CTTACTGCTTGAAAGGGTCTT
44	hsa_miR_1265	-13.9	CTTACTGCTTGAAAGGGTCTTG
625	hsa_miR_6895_5p	-16	CTTACTGCTTGAAAGGGTCTTG
471	hsa_miR_611	-14.8	CTTACTGCTTGAAAGGGTCTTGC
271	hsa_miR_4417	-16.1	CTTGAAAGGGTCTTGCCTCACC
524	hsa_miR_6742_5p	-24.3	CTTGAAAGGGTCTTGCCTCACC
328	hsa_miR_4649_5p	-22.6	CTTGAAAGGGTCTTGCCTCACCCA

548	hsa_miR_6766_5p	-25	CTTGAAAGGGTCTTGCCTCACCCA
39	hsa_miR_1251_5p	-19.4	CTTGCCTCACCCAAGCTAGAGT
212	hsa_miR_3655	-12.3	AGCCATTGGCACACAGGC
672	hsa_miR_889_5p	-14.7	AGCCATTGGCACACAGGCATT
399	hsa_miR_5006_5p	-18.9	AGCCTAACTCAAGCCATTGGCAC
602	hsa_miR_6850_5p	-15.1	AGCCTAACTCAAGCCATTGGCAC
236	hsa_miR_3911	-15.6	AGCCTAACTCAAGCCATTGGCACA
281	hsa_miR_4449	-15.8	AGCCTAACTCAAGCCATTGGCACA
508	hsa_miR_664b_5p	-15.9	AGCCTAACTCAAGCCATTGGCACA
204	hsa_miR_3620_5p	-22.4	AGCCTAACTCAAGCCATTGGCACAC
62	hsa_miR_1307_5p	-15.7	AGCTGGAAGTTGAAATGGTGGA
739	hsa_miR_6754_3p	-14.3	AGCTGGAAGTTGAAATGGTGGAGT
438	hsa_miR_5572	-13.7	AGCTTACTACAGCCTCAAA
372	hsa_miR_4748	-14	AGCTTACTACAGCCTCAAACTTC
183	hsa_miR_3194_3p	-15.44	CTTGCCTCACCCAAGCTAGAGTG
286	hsa_miR_4472	-12.5	GAAAGGGTCTTGCCTCACC
76	hsa_miR_149_3p	-13.4	GAAAGGGTCTTGCCTCACCCA
478	hsa_miR_626	-17.2	AGGACATGAAGACAGTT
579	hsa_miR_6813_5p	-22.4	AGGACATGAAGACAGTTCCTG
242	hsa_miR_3937	-12.2	AGGACATGAAGACAGTTCCTGT
669	hsa_miR_873_5p	-15.2	AGGACATGAAGACAGTTCCTGT
652	hsa_miR_770_5p	-14.7	AGGCATTAGACAGAAAGCTGGA
71	hsa_miR_145_5p	-16.3	AGGCATTAGACAGAAAGCTGGAA
72	hsa_miR_1468_5p	-12.2	AGGCATTAGACAGAAAGCTGGAA
20	hsa_miR_1180_3p	-15.8	AGGCATTAGACAGAAAGCTGGAAG
529	hsa_miR_6745	-16	GAAAGGGTCTTGCCTCACCCA
96	hsa_miR_190b	-13	GACCACAACAAGCAAGCATGCA
198	hsa_miR_34c_5p	-19	GCAAGCATGCAGCTTACTGCTT
298	hsa_miR_449b_5p	-18.3	GCAAGCATGCAGCTTACTGCTT
346	hsa_miR_4690_5p	-18.9	GCAAGCATGCAGCTTACTGCTT
506	hsa_miR_663a	-13.3	GCAAGCATGCAGCTTACTGCTT
103	hsa_miR_196b_5p	-13.1	GCAAGCATGCAGCTTACTGCTTG
623	hsa_miR_6893_5p	-14.8	GCAAGCATGCAGCTTACTGCTTG
159	hsa_miR_3127_3p	-17.8	GCAGCTTACTGCTTGAAAGGGT
338	hsa_miR_4674	-14.3	AGGTATGGTGCTGGGTGCAG
24	hsa_miR_1199_5p	-16	AGGTATGGTGCTGGGTGCAGA
437	hsa_miR_557	-13.3	AGGTATGGTGCTGGGTGCAGAT
110	hsa_miR_200b_5p	-18.1	AGGTATGGTGCTGGGTGCAGATG
111	hsa_miR_200c_5p	-21.2	AGGTATGGTGCTGGGTGCAGATG
259	hsa_miR_4280	-15.4	AGTCGGCTTGACTACACTG
330	hsa_miR_4651	-16.6	AGTCGGCTTGACTACACTGTG
488	hsa_miR_6499_3p	-17.6	AGTCGGCTTGACTACACTGTG
378	hsa_miR_4761_5p	-15.4	AGTCGGCTTGACTACACTGTGT
321	hsa_miR_3937	-12.7	AGTCGGCTTGACTACACTGTGTGG
628	hsa_miR_7109_5p	-19.3	AGTGATCCTCAGCCTCCCAG
335	hsa_miR_4667_5p	-20.3	AGTGATCCTCAGCCTCCCAGT
589	hsa_miR_5572	-18	AGTGATCCTCAGCCTCCCAGT

42	hsa_miR_126_5p	-14.1	AGTGTAACCAATGATGTAATGATT
168	hsa_miR_3152_3p	-13.5	AGTGTGGCTCTGGATAGCACC
544	hsa_miR_6762_3p	-13	AGTGTGGCTCTGGATAGCACCT
142	hsa_miR_296_3p	-13.9	AGTGTGGCTCTGGATAGCACCTT
264	hsa_miR_431_3p	-16.5	AGTGTGGCTCTGGATAGCACCTT
744	hsa_miR_6756_5p	-16.4	AGTGTGGCTCTGGATAGCACCTT
92	hsa_miR_18b_5p	-21.4	AGTGTGGCTCTGGATAGCACCTTA
187	hsa_miR_328_5p	-12.5	AGTGTGGCTCTGGATAGCACCTTA
567	hsa_miR_6789_5p	-22.6	AGTGTGGCTCTGGATAGCACCTTAT
185	hsa_miR_3200_5p	-12.5	ATGCAGTGTGGCTCTGGATA
258	hsa_miR_4277	-13.4	ATGCAGTGTGGCTCTGGATAGC
536	hsa_miR_6753_3p	-17.4	ATGCAGTGTGGCTCTGGATAGCACCT
318	hsa_miR_454_5p	-14.1	GCAGCTTACTGCTTGAAAGGGT
614	hsa_miR_6877_5p	-22.1	GCAGCTTACTGCTTGAAAGGGTCTT
479	hsa_miR_630	-14.3	ATGGTGCTGGGTGCAGATGCA
559	hsa_miR_6781_3p	-14	ATGGTGCTGGGTGCAGATGCA
674	hsa_miR_891b	-12.5	ATGGTGCTGGGTGCAGATGCA
309	hsa_miR_452_3p	-15.1	ATGGTGCTGGGTGCAGATGCAG
169	hsa_miR_3152_5p	-15.4	ATGGTGCTGGGTGCAGATGCAGT
574	hsa_miR_6807_3p	-25.9	ATGGTGCTGGGTGCAGATGCAGTG
680	hsa_miR_92a_3p	-22.9	ATGGTGCTGGGTGCAGATGCAGTG
547	hsa_miR_6765_3p	-20.7	ATGGTGCTGGGTGCAGATGCAGTGTGG
436	hsa_miR_554	-14.4	ATTGGCACACAGGCATTAGA
640	hsa_miR_7155_5p	-13.5	ATTGGCACACAGGCATTAGA
489	hsa_miR_6499_5p	-14.3	GCATGCAGCTTACTGCTTGA
532	hsa_miR_6749_5p	-13.5	GCATGCAGCTTACTGCTTGA
568	hsa_miR_6792_5p	-14	GCATGCAGCTTACTGCTTGA
138	hsa_miR_27b_3p	-12.6	GCATGCAGCTTACTGCTTGAA
230	hsa_miR_378a_3p	-14.9	GCCTCACCCAAGCTAGA
232	hsa_miR_378e	-14.5	GCCTCACCCAAGCTAGA
248	hsa_miR_422a	-14.9	GCCTCACCCAAGCTAGA
638	hsa_miR_7152_3p	-17	GCCTCACCCAAGCTAGA
189	hsa_miR_330_5p	-12.6	GCCTCACCCAAGCTAGAGT
43	hsa_miR_1264	-13.7	GCTTACTGCTTGAAAGGGTCTTG
214	hsa_miR_365a_5p	-15.8	GCTTACTGCTTGAAAGGGTCTTG
217	hsa_miR_3667_5p	-18.1	GCTTACTGCTTGAAAGGGTCTTG
457	hsa_miR_598_5p	-17.4	GCTTACTGCTTGAAAGGGTCTTGC
152	hsa_miR_3116	-12	CAAGCCATTGGCACACAGGCA
366	hsa_miR_4440	-20.4	CAAGCCATTGGCACACAGGCA
44	hsa_miR_1231	-15.6	CAAGCCATTGGCACACAGGCAT
181	hsa_miR_297	-17.3	CAAGCCATTGGCACACAGGCAT
800	hsa_miR_6813_5p	-16.1	CAAGCCTAACTCAAGCCATTG
196	hsa_miR_34a_5p	-12.3	CAAGCCTAACTCAAGCCATTGGCA
293	hsa_miR_4492	-16.7	CAAGTGATCCTCAGCCTC
850	hsa_miR_6884_5p	-17.5	CAAGTGATCCTCAGCCTCC
250	hsa_miR_423_5p	-13.6	CAAGTGATCCTCAGCCTCCCA
262	hsa_miR_4300	-16.7	CAAGTGATCCTCAGCCTCCCA

563	hsa_miR_6785_5p	-19.8	CAAGTGATCCTCAGCCTCCCA
148	hsa_miR_30b_3p	-23.2	CAAGTGATCCTCAGCCTCCCAG
359	hsa_miR_4722_5p	-12.1	CAATGATGTAATGATTCTGCC
375	hsa_miR_4755_3p	-13.7	GCTTACTGCTTGAAAGGGTCTTGCC
323	hsa_miR_4634	-12.5	GCTTGAAAGGGTCTTGCCT
682	hsa_miR_92b_5p	-12.4	GCTTGAAAGGGTCTTGCCTC
687	hsa_miR_942_5p	-16.9	CACAGGCATTAGACAGAAAGC
175	hsa_miR_3160_5p	-16.3	CACAGGCATTAGACAGAAAGCT
560	hsa_miR_6782_3p	-12.9	CACAGGCATTAGACAGAAAGCTG
398	hsa_miR_5004_5p	-17	GCTTGAAAGGGTCTTGCCTCA
27	hsa_miR_1203	-22.8	CAGATGCAGTGTGGCTCTGGA
386	hsa_miR_4787_5p	-16.7	CAGATGCAGTGTGGCTCTGGA
514	hsa_miR_6727_5p	-13.1	CAGATGCAGTGTGGCTCTGGA
634	hsa_miR_7113_5p	-18.7	CAGATGCAGTGTGGCTCTGGA
25	hsa_miR_1200	-19.2	CAGATGCAGTGTGGCTCTGGAT
417	hsa_miR_518d_5p	-17	CAGATGCAGTGTGGCTCTGGAT
418	hsa_miR_518e_5p	-16.6	CAGATGCAGTGTGGCTCTGGAT
419	hsa_miR_518f_5p	-14.4	CAGATGCAGTGTGGCTCTGGAT
422	hsa_miR_519a_5p	-16.6	CAGATGCAGTGTGGCTCTGGAT
423	hsa_miR_519b_5p	-16.6	CAGATGCAGTGTGGCTCTGGAT
424	hsa_miR_519c_5p	-16.6	CAGATGCAGTGTGGCTCTGGAT
425	hsa_miR_520c_5p	-17	CAGATGCAGTGTGGCTCTGGAT
427	hsa_miR_522_5p	-16.6	CAGATGCAGTGTGGCTCTGGAT
428	hsa_miR_523_5p	-16.6	CAGATGCAGTGTGGCTCTGGAT
430	hsa_miR_526a	-17	CAGATGCAGTGTGGCTCTGGAT
167	hsa_miR_3151_5p	-20.9	GCTTGAAAGGGTCTTGCCTCACC
667	hsa_miR_8085	-17.8	GCTTGAAAGGGTCTTGCCTCACCCA
686	hsa_miR_939_5p	-19.4	GCTTGAAAGGGTCTTGCCTCACCCA
653	hsa_miR_626	-14.6	CAGGCATTAGACAGAAAGCT
114	hsa_miR_21_5p	-12.6	CAGGCATTAGACAGAAAGCTG
57	hsa_miR_1301_3p	-15.5	CAGGCATTAGACAGAAAGCTGGAA
362	hsa_miR_4729	-12.1	CAGTGTAACCAATGATGTAATGA
319	hsa_miR_455_3p	-16.6	CAGTGTGGCTCTGGATAGC
85	hsa_miR_182_3p	-13.6	CAGTGTGGCTCTGGATAGCACCT
531	hsa_miR_6747_5p	-16.7	CAGTGTGGCTCTGGATAGCACCTTA
310	hsa_miR_4520_5p	-15.8	CATGATTGAAGAAATGCAGG
528	hsa_miR_6744_5p	-12.4	GGGTCTTGCCTCACCCA
671	hsa_miR_887_5p	-15.3	GGGTCTTGCCTCACCCAAG
77	hsa_miR_149_5p	-14.79	GGGTCTTGCCTCACCCAAGCTAGA
265	hsa_miR_4315	-13.2	CATTAGACAGAAAGCTGG
238	hsa_miR_3925_3p	-13.6	CATTAGACAGAAAGCTGGAAGT
254	hsa_miR_33b_3p	-12.7	CATTGGCACACAGGCATTA
300	hsa_miR_4502	-14.3	CATTGGCACACAGGCATTAGA
390	hsa_miR_4799_5p	-14.8	CATTGGCACACAGGCATTAGAC
439	hsa_miR_5579_3p	-12	GGTCTTGCCTCACCCAAGCTAG
379	hsa_miR_4763_3p	-12.6	CCAATGATGTAATGATTCTGCCA
262	hsa_miR_34a_5p	-14.7	CCAATGATGTAATGATTCTGCCA

231	hsa_miR_378c	-13.8	GGTCTTGCCTCACCCAAGCTAGA
144	hsa_miR_301b_5p	-12.4	GGTCTTGCCTCACCCAAGCTAGAGT
522	hsa_miR_6740_5p	-12.2	GTATTATTTTTCATCTTCAAACA
402	hsa_miR_5008_5p	-15.8	CCATTGGCACACAGGCATTA
683	hsa_miR_934	-12.5	CCATTGGCACACAGGCATTAGACA
325	hsa_miR_4639_5p	-14.2	CCTAACTCAAGCCATTGGCAC
336	hsa_miR_4669	-14.2	CCTAACTCAAGCCATTGGCACA
575	hsa_miR_6807_5p	-22.1	CCTAACTCAAGCCATTGGCACAC
646	hsa_miR_760	-15	GTCTTGCCTCACCCAAGCTA
382	hsa_miR_4774_5p	-12.4	GTCTTGCCTCACCCAAGCTAGA
51	hsa_miR_1287_3p	-14.8	GTCTTGCCTCACCCAAGCTAGAG
415	hsa_miR_514a_5p	-12.6	GTCTTGCCTCACCCAAGCTAGAGTG
290	hsa_miR_4486	-19.4	CCTCAGCCTCCCAGT
240	hsa_miR_3929	-18.5	CCTTTGAAGCTTACTACAGCCTC
132	hsa_miR_2467_5p	-15.7	CCTTTGAAGCTTACTACAGCCTCA
21	hsa_miR_1181	-13.66	CGGCTTGACTACACTGTGTGG
337	hsa_miR_4672	-12.2	CGGCTTGACTACACTGTGTGG
356	hsa_miR_4717_3p	-16.7	CGGCTTGACTACACTGTGTGG
391	hsa_miR_4804_5p	-14.1	CGGCTTGACTACACTGTGTGG
902	hsa_miR_770_5p	-12.9	CGGCTTGACTACACTGTGTGGA
26	hsa_miR_1202	-17.5	CTAACTCAAGCCATTGGCAC
569	hsa_miR_6793_5p	-13.7	CTAACTCAAGCCATTGGCACACA
256	hsa_miR_4265	-14.1	CTCAAGCCATTGGCACACAG
401	hsa_miR_5008_3p	-16.4	CTCAAGCCATTGGCACACAGG
451	hsa_miR_5705	-14.3	CTCAAGCCATTGGCACACAGGCA
811	hsa_miR_6821_5p	-18.1	CTCAAGCCATTGGCACACAGGCAT
29	hsa_miR_1205	-13	CTCAAGTGATCCTCAGC
622	hsa_miR_604	-14.4	CTCAAGTGATCCTCAGCCT
494	hsa_miR_4748	-18.5	CTCAAGTGATCCTCAGCCTC
179	hsa_miR_3180	-14.5	CTCAAGTGATCCTCAGCCTCC
228	hsa_miR_3689d	-17.1	CTCAAGTGATCCTCAGCCTCCC
592	hsa_miR_6835_5p	-14.9	CTCAAGTGATCCTCAGCCTCCC
626	hsa_miR_7106_5p	-25.9	CTCAAGTGATCCTCAGCCTCCCA
248	hsa_miR_328_5p	-22	CTCAAGTGATCCTCAGCCTCCCA
707	hsa_miR_6731_5p	-18.1	CTCAAGTGATCCTCAGCCTCCCA
870	hsa_miR_7110_5p	-20	CTCAAGTGATCCTCAGCCTCCCA
515	hsa_miR_6728_5p	-18.2	CTCAAGTGATCCTCAGCCTCCCAG
520	hsa_miR_6737_5p	-13	CTCAAGTGATCCTCAGCCTCCCAG
182	hsa_miR_3192_5p	-21.5	CTCAAGTGATCCTCAGCCTCCCAGT
283	hsa_miR_4454	-13.7	TACTGCTTGAAAGGGTCT
621	hsa_miR_6892_5p	-13.4	TACTGCTTGAAAGGGTCTTGC
461	hsa_miR_6069	-15.6	TACTGCTTGAAAGGGTCTTGCCT
583	hsa_miR_6817_5p	-17.8	TCACCCAAGCTAGAGTGCAGT
218	hsa_miR_367_3p	-25.5	TCACCCAAGCTAGAGTGCAGTG
253	hsa_miR_4257	-12.7	CTCTGGATAGCACCTTATGG
649	hsa_miR_616_3p	-12.9	CTGCCAAATGAAATATAATGATA
676	hsa_miR_892b	-17	TCACCCAAGCTAGAGTGCAGTG

282	hsa_miR_4453	-13.7	TCTTGCCTCACCCAAGCTA
301	hsa_miR_4505	-14	TCTTGCCTCACCCAAGCTA
61	hsa_miR_1307_3p	-13.8	TCTTGCCTCACCCAAGCTAGAGT
324	hsa_miR_4638_5p	-16.9	TCTTGCCTCACCCAAGCTAGAGT
644	hsa_miR_744_5p	-13.9	TGAAAGGGTCTTGCCTCACC
209	hsa_miR_363_5p	-12.3	TGAAAGGGTCTTGCCTCACCCA
631	hsa_miR_7111_5p	-17.7	TGAAAGGGTCTTGCCTCACCCA
45	hsa_miR_1271_3p	-12.4	TGACCACAACAAGCAAGCATG
766	hsa_miR_6777_3p	-13.1	CTGGAAGTTGAAATGGTGGA
466	hsa_miR_6084	-18.9	CTGGAAGTTGAAATGGTGGAG
546	hsa_miR_6763_3p	-15.6	CTGGAAGTTGAAATGGTGGAG
247	hsa_miR_412_3p	-16.6	CTGGAAGTTGAAATGGTGGAGT
594	hsa_miR_6837_3p	-13.8	CTGGAAGTTGAAATGGTGGAGT
70	hsa_miR_144_5p	-12.5	CTGGAAGTTGAAATGGTGGAGTCC
635	hsa_miR_7114_3p	-15.6	CTGGAAGTTGAAATGGTGGAGTCC
581	hsa_miR_6815_3p	-12.7	CTGGAAGTTGAAATGGTGGAGTCCA
627	hsa_miR_6069	-13.5	CTGGGCTCAAGTGATCCTCAGCCT
33	hsa_miR_1228_3p	-18.7	CTGGGTGCAGATGCAGTGTGG
203	hsa_miR_3620_3p	-30.7	CTGGGTGCAGATGCAGTGTGG
578	hsa_miR_6812_3p	-18.4	CTGGGTGCAGATGCAGTGTGG
313	hsa_miR_4529_3p	-14.7	CTGGGTGCAGATGCAGTGTGGC
554	hsa_miR_6777_3p	-17.3	CTGGGTGCAGATGCAGTGTGGC
130	hsa_miR_24_2_5p	-16.3	CTGGGTGCAGATGCAGTGTGGCT
320	hsa_miR_4632_3p	-18.8	CTGGGTGCAGATGCAGTGTGGCT
102	hsa_miR_196a_3p	-13.1	CTGGGTGCAGATGCAGTGTGGCTC
201	hsa_miR_3619_3p	-17.9	CTGGGTGCAGATGCAGTGTGGCTC
527	hsa_miR_5001_5p	-15.5	CTGGGTGCAGATGCAGTGTGGCTCT
211	hsa_miR_3651	-12.6	CTGGGTGCAGATGCAGTGTGGCTCTG
463	hsa_miR_608	-15	CTGGGTGCAGATGCAGTGTGGCTCTG
448	hsa_miR_5696	-15.9	TGCAGCTTACTGCTTGAAAGGG
458	hsa_miR_601	-12	TGCCTCACCCAAGCTAGAGTG
388	hsa_miR_4793_3p	-20.1	TGCCTCACCCAAGCTAGAGTGCAGT
134	hsa_miR_25_5p	-14.4	TGCTTGAAAGGGTCTTGCCT
151	hsa_miR_31_5p	-21.2	TGCTTGAAAGGGTCTTGCCT
5	hsa_let_7e_5p	-15.3	TGCTTGAAAGGGTCTTGCCTCA
334	hsa_miR_4661_3p	-12.7	CTTCTGGGCTCAAGTGATCCTC
306	hsa_miR_450b_3p	-12.7	CTTCTGGGCTCAAGTGATCCTCAG
158	hsa_miR_3126_5p	-14.9	TGCTTGAAAGGGTCTTGCCTCA
35	hsa_miR_1238_5p	-14.8	TGCTTGAAAGGGTCTTGCCTCAC
235	hsa_miR_381_5p	-15.7	TGCTTGAAAGGGTCTTGCCTCACC
162	hsa_miR_3132	-16.5	TGCTTGAAAGGGTCTTGCCTCACCCA
660	hsa_miR_8068	-16.9	TTAATGACCACAACAAGCA
609	hsa_miR_6864_3p	-12.6	TTGAAAGGGTCTTGCCTCAC
432	hsa_miR_541_3p	-15.1	TTGAAAGGGTCTTGCCTCACCC
533	hsa_miR_675_5p	-18.5	TTGAAAGGGTCTTGCCTCACCC
537	hsa_miR_6754_3p	-14.1	CTTGGGTAAGGACATGAAGA
481	hsa_miR_636	-13.9	CTTGGGTAAGGACATGAAGACA

539	hsa_miR_6755_3p	-18	CTTGGGTAAGGACATGAAGACA
616	hsa_miR_6884_5p	-15.6	CTTTGAAGCTTACTACAGCCTCA
340	hsa_miR_4676_3p	-16	GAAAGCTGGAAGTTGAAATGGTG
277	hsa_miR_3622b_5	13.4	CTGCTTGAAAGGGTCTTGCCT
278	hsa_miR_3622b_5	15	GGTCTTGCCTCACCCAAGCTA
607	hsa_miR_5698	-16.3	AAAGGGTCTTGCCTCACCCA
893	hsa_miR_762	-20.8	AAAGGGTCTTGCCTCACCCA
718	hsa_miR_6741_5p	-22.9	AAAGGGTCTTGCCTCACCCAA
768	hsa_miR_6779_5p	-17.1	AAAGGGTCTTGCCTCACCCAA
735	hsa_miR_6751_5p	-14.6	GAAGATTAAAACAGTGTCTCCAA
273	hsa_miR_4419b	-14.2	GAAGCTTACTACAGCCTC
393	hsa_miR_485_5p	-14.6	GAAGCTTACTACAGCCTCA
473	hsa_miR_6134	-14.1	GAAGCTTACTACAGCCTCA
287	hsa_miR_4474_3p	-15.3	GAAGCTTACTACAGCCTCAA
409	hsa_miR_5087	-12	GAAGCTTACTACAGCCTCAAACTT
641	hsa_miR_610	-14.7	AACAAGCAAGCATGCAGCTTA
513	hsa_miR_4787_5p	-12.7	AACAAGCAAGCATGCAGCTTACTGC
924	hsa_miR_8081	-13.8	GACTCAGAATTCATGATTGAAG
284	hsa_miR_4466	-15.4	GAGAGTCGGCTTGACTA
186	hsa_miR_323a_5p	-13.4	GAGAGTCGGCTTGACTACACT
414	hsa_miR_512_5p	-13.12	GAGAGTCGGCTTGACTACACTGTGTG
661	hsa_miR_8069	-12.9	GAGCAAGCCTAACTCAAGCCATTG
98	hsa_miR_1913	-16.7	GAGGTATGGTGCTGGGTGCAGA
645	hsa_miR_758_3p	-13.9	GAGGTATGGTGCTGGGTGCAGA
125	hsa_miR_2277_5p	-12.9	GAGTCGGCTTGACTACACTGTGTG
197	hsa_miR_34b_5p	-19.3	GAGTCGGCTTGACTACACTGTGTG
594	hsa_miR_5580_3p	-16	GAGTCGGCTTGACTACACTGTGTG
174	hsa_miR_3158_5p	-12.6	GAGTCGGCTTGACTACACTGTGTGG
224	hsa_miR_3687	-14.5	GAGTCGGCTTGACTACACTGTGTGGA
373	hsa_miR_4749_5p	-15.9	GATCCTCAGCCTCCCAGTG
856	hsa_miR_6890_5p	-13.8	GATCCTCAGCCTCCCAGTG
473	hsa_miR_4717_3p	-22	GATCCTCAGCCTCCCAGTGG
19	hsa_miR_1178_5p	-14	GATGCAGTGTGGCTCTG
263	hsa_miR_4302	-12.6	GATGCAGTGTGGCTCTGG
487	hsa_miR_645	-14.1	GATGCAGTGTGGCTCTGGA
136	hsa_miR_26a_1_3	-16.6	GATGCAGTGTGGCTCTGGATAGC
155	hsa_miR_3120_5p	-17.2	GATGCAGTGTGGCTCTGGATAGC
493	hsa_miR_6501_5p	-18.7	GATGCAGTGTGGCTCTGGATAGCA
650	hsa_miR_767_5p	-12.2	GATTTTCAGTGTAACCAATGATGTA
389	hsa_miR_4498	-15.3	AAGCAAGCATGCAGCTTA
563	hsa_miR_5190	-12.2	AAGCAAGCATGCAGCTTACTGC
374	hsa_miR_4466	-15.1	AAGGGTCTTGCCTCACCC
436	hsa_miR_4656	-21.3	AAGGGTCTTGCCTCACCCA
843	hsa_miR_6870_5p	-12.4	AAGGGTCTTGCCTCACCCA
324	hsa_miR_3940_5p	-23.8	AAGGGTCTTGCCTCACCCAA
400	hsa_miR_4507	-20	AAGGGTCTTGCCTCACCCAA
626	hsa_miR_6069	-15.6	AAGGGTCTTGCCTCACCCAAGCTA

28	hsa_miR_1184	-13.2	AATGACCACAACAAGCAAGCATGCAGC
243	hsa_miR_3940_5p	-20.1	GCAAGCCTAACTCAAGCCAT
302	hsa_miR_4507	-18.7	GCAAGCCTAACTCAAGCCAT
431	hsa_miR_532_5p	-13.8	GCAAGCCTAACTCAAGCCATT
221	hsa_miR_3677_5p	-13.2	GCAAGCCTAACTCAAGCCATTG
657	hsa_miR_632	-14.3	GCACACAGGCATTAGACAG
147	hsa_miR_30a_3p	-15.9	GCACACAGGCATTAGACAGAAAG
149	hsa_miR_30d_3p	-17.3	GCACACAGGCATTAGACAGAAAG
150	hsa_miR_30e_3p	-12.7	GCACACAGGCATTAGACAGAAAG
280	hsa_miR_4446_3p	-16.6	GCAGATGCAGTGTGGCTCTG
408	hsa_miR_505_5p	-16.6	GCAGATGCAGTGTGGCTCTG
540	hsa_miR_6755_5p	-14.2	GCAGATGCAGTGTGGCTCTG
599	hsa_miR_6845_5p	-15.5	GCAGATGCAGTGTGGCTCTG
806	hsa_miR_6816_5p	-15.4	GCAGATGCAGTGTGGCTCTG
56	hsa_miR_1296_5p	-17	GCAGATGCAGTGTGGCTCTGG
202	hsa_miR_3619_5p	-13.1	GCAGATGCAGTGTGGCTCTGG
450	hsa_miR_5704	-14.6	GCAGATGCAGTGTGGCTCTGG
199	hsa_miR_3591_5p	-15.3	GCAGATGCAGTGTGGCTCTGGA
595	hsa_miR_6837_5p	-18.6	GCAGATGCAGTGTGGCTCTGGA
732	hsa_miR_6751_5p	-14.4	GCAGATGCAGTGTGGCTCTGGA
64	hsa_miR_1343_3p	-18.9	GCAGATGCAGTGTGGCTCTGGAT
7	hsa_let_7g_3p	-19.3	GCAGATGCAGTGTGGCTCTGGATAG
149	hsa_miR_221_3p	-13.6	ACAACAAGCAAGCATGCAGCT
685	hsa_miR_656_5p	-12.8	ACAACAAGCAAGCATGCAGCTT
114	hsa_miR_18b_5p	-12.2	ACAACAAGCAAGCATGCAGCTTA
953	hsa_miR_99b_3p	-14.9	ACAACAAGCAAGCATGCAGCTTA
444	hsa_miR_5586_5p	-15.5	GCAGTGTGGCTCTGGATA
668	hsa_miR_8086	-14	GCAGTGTGGCTCTGGATAGCA
193	hsa_miR_33b_5p	-17.6	GCAGTGTGGCTCTGGATAGCAC
394	hsa_miR_487a_5p	-13.4	GCAGTGTGGCTCTGGATAGCAC
349	hsa_miR_4694_5p	-23.9	GCAGTGTGGCTCTGGATAGCACCT
358	hsa_miR_4721	-21.1	GCAGTGTGGCTCTGGATAGCACCTTA
601	hsa_miR_6848_5p	-20.6	GCAGTGTGGCTCTGGATAGCACCTTA
323	hsa_miR_3940_5p	-15.3	ACAAGCAAGCATGCAGCTTAC
152	hsa_miR_222_3p	-13.1	ACCACAACAAGCAAGCATGCAGCT
934	hsa_miR_891a_3p	-17.7	ACCACAACAAGCAAGCATGCAGCT
613	hsa_miR_5787	-12.7	ACCACAACAAGCAAGCATGCAGCTT
188	hsa_miR_30a_3p	-14.1	GCATTAGACAGAAAGCTGGAAG
192	hsa_miR_30d_3p	-16.8	GCATTAGACAGAAAGCTGGAAG
195	hsa_miR_30e_3p	-14.4	GCATTAGACAGAAAGCTGGAAG
317	hsa_miR_454_3p	-12.8	GCCATTGGCACACAGGCATTA
80	hsa_miR_155_5p	-12.8	GCCATTGGCACACAGGCATTAG
15	hsa_miR_105_5p	-13.9	GCCATTGGCACACAGGCATTAGA
31	hsa_miR_1199_5p	-12	GCCATTGGCACACAGGCATTAGA
922	hsa_miR_8078	-12.9	GCCATTGGCACACAGGCATTAGACA
562	hsa_miR_6784_5p	-20.3	GCCTAACTCAAGCCATTGGC
928	hsa_miR_8086	-12.7	GCCTAACTCAAGCCATTGGCA

586	hsa_miR_6821_5p	-23.1	GCCTAACTCAAGCCATTGGCAC
885	hsa_miR_7160_3p	-21.4	ACTGCTTGAAAGGGTCTTG
492	hsa_miR_4746_3p	-12.1	ACTGCTTGAAAGGGTCTTGCC
601	hsa_miR_5589_5p	-17	ACTGCTTGAAAGGGTCTTGCC
505	hsa_miR_4763_3p	-19.5	ACTGCTTGAAAGGGTCTTGCCT
170	hsa_miR_2682_5p	-15.1	ACTGCTTGAAAGGGTCTTGCCTC
668	hsa_miR_6499_3p	-13.5	AGCAAGCATGCAGCTTACTGCT
462	hsa_miR_6073	-12.9	GCCTTTGAAGCTTACTACA
188	hsa_miR_329_5p	-12.7	GCCTTTGAAGCTTACTACAGCCTC
529	hsa_miR_5001_5p	-15	GCCTTTGAAGCTTACTACAGCCTC
729	hsa_miR_6747_5p	-14.8	GCCTTTGAAGCTTACTACAGCCTC
564	hsa_miR_6787_5p	-15.5	GCTCAAGTGATCCTCAGCCT
894	hsa_miR_762	-21.76	GCTCAAGTGATCCTCAGCCTC
468	hsa_miR_6087	-19.4	GCTCAAGTGATCCTCAGCCTCC
385	hsa_miR_4489	-12.2	GCTCAAGTGATCCTCAGCCTCC
522	hsa_miR_485_5p	-13	GCTCAAGTGATCCTCAGCCTCC
23	hsa_miR_1193	-14.7	GCTCAAGTGATCCTCAGCCTCCC
535	hsa_miR_6752_5p	-22.2	GCTCAAGTGATCCTCAGCCTCCC
70	hsa_miR_1293	-20	GCTCAAGTGATCCTCAGCCTCCCA
701	hsa_miR_6726_5p	-20.1	GCTCAAGTGATCCTCAGCCTCCCA
227	hsa_miR_3689c	-20.8	GCTCAAGTGATCCTCAGCCTCCCAG
351	hsa_miR_4700_5p	-20.1	GCTCAAGTGATCCTCAGCCTCCCAGT
482	hsa_miR_637	-19.9	GCTCAAGTGATCCTCAGCCTCCCAGT
459	hsa_miR_604	-12	GCTCTGGATAGCACCT
440	hsa_miR_558	-12.2	GCTCTGGATAGCACCTTA
588	hsa_miR_6824_5p	-17.6	GCTCTGGATAGCACCTTAT
270	hsa_miR_433_3p	-14	GCTCTGGATAGCACCTTATGGA
104	hsa_miR_197_3p	-17.7	GCTGGAAGTTGAAATGGTGGA
66	hsa_miR_136_5p	-14	GCTGGAAGTTGAAATGGTGGAGT
412	hsa_miR_5096	-13.8	GCTGGAAGTTGAAATGGTGGAGT
649	hsa_miR_766_3p	-17.7	GCTGGAAGTTGAAATGGTGGAGT
476	hsa_miR_617	-15.4	GCTGGAAGTTGAAATGGTGGAGTCC
410	hsa_miR_509_3_5	-13.2	GCTGGGTGCAGATGCAGTG
68	hsa_miR_139_5p	-23.5	GCTGGGTGCAGATGCAGTGTGGC
229	hsa_miR_370_3p	-18.4	GCTGGGTGCAGATGCAGTGTGGC
316	hsa_miR_4539	-19.1	GCTGGGTGCAGATGCAGTGTGGC
12	hsa_miR_103a_3p	-14.4	GCTGGGTGCAGATGCAGTGTGGCT
18	hsa_miR_107	-14.4	GCTGGGTGCAGATGCAGTGTGGCT
129	hsa_miR_24_1_5p	-19.6	GCTGGGTGCAGATGCAGTGTGGCT
610	hsa_miR_6869_3p	-29.4	GCTGGGTGCAGATGCAGTGTGGCT
598	hsa_miR_5589_5p	-13.6	GCTGGGTGCAGATGCAGTGTGGCT
442	hsa_miR_5582_5p	-13.2	GCTGGGTGCAGATGCAGTGTGGCTC
637	hsa_miR_7151_5p	-18.9	GCTGGGTGCAGATGCAGTGTGGCTC
331	hsa_miR_4656	-13.7	GCTGGGTGCAGATGCAGTGTGGCTCT
891	hsa_miR_762	-16.9	GCTGGGTGCAGATGCAGTGTGGCTCT
169	hsa_miR_2682_5p	-17.4	AGCAAGCATGCAGCTTACTGCTTG
353	hsa_miR_431_3p	-13.8	AGCAAGCATGCAGCTTACTGCTTG

760	hsa_miR_6772_5p	-16.2	AGCAAGCATGCAGCTTACTGCTTG
526	hsa_miR_498	-15.5	AGCATGCAGCTTACTGCTTGAAA
174	hsa_miR_27a_5p	-13.8	AGCTTACTGCTTGAAAGGGTCTT
468	hsa_miR_4710	-19	AGGGTCTTGCCTCACCC
557	hsa_miR_518d_5p	-12.9	AGGGTCTTGCCTCACCCAAGCTAGAG
561	hsa_miR_518f_5p	-12.9	AGGGTCTTGCCTCACCCAAGCTAGAG
573	hsa_miR_520c_5p	-12.9	AGGGTCTTGCCTCACCCAAGCTAGAG
581	hsa_miR_526a	-12.9	AGGGTCTTGCCTCACCCAAGCTAGAG
273	hsa_miR_3620_5p	-17	CAACAAGCAAGCATGCAGCTTAC
849	hsa_miR_6884_5p	-12.3	CAACAAGCAAGCATGCAGCTTACT
132	hsa_miR_199a_3p	-14.6	CAAGCAAGCATGCAGCTTACTGC
135	hsa_miR_199b_3p	-14.6	CAAGCAAGCATGCAGCTTACTGC
367	hsa_miR_4440	-17.6	CAAGCAAGCATGCAGCTTACTGCT
491	hsa_miR_4746_3p	-15.9	CAAGCATGCAGCTTACTGCT
862	hsa_miR_6895_3p	-13.3	GCTTGGGTAAGGACATGAAGACA
368	hsa_miR_4742_3p	-13.6	GCTTGGGTAAGGACATGAAGACAGT
227	hsa_miR_3160_5p	-14.5	GGAAGTTGAAATGGTGGAGTC
943	hsa_miR_92b_3p	-12.6	GGAAGTTGAAATGGTGGAGTC
153	hsa_miR_3118	-13.4	GGAAGTTGAAATGGTGGAGTCCA
684	hsa_miR_937_5p	-15.8	GGAGAGTCGGCTTGACTACAC
119	hsa_miR_216a_3p	-13	GGAGAGTCGGCTTGACTACACTGTGT
234	hsa_miR_381_3p	-14.9	GGAGAGTCGGCTTGACTACACTGTGTG
10	hsa_miR_101_5p	-14.6	GGCATTAGACAGAAAGCTG
421	hsa_miR_5195_3p	-16.3	GGCATTAGACAGAAAGCTGGAA
163	hsa_miR_3135b	-13	GGCCTTTGAAGCTTACTACAGCC
920	hsa_miR_8077	-17.6	GGCCTTTGAAGCTTACTACAGCC
587	hsa_miR_6822_3p	-12.5	GGCCTTTGAAGCTTACTACAGCCT
469	hsa_miR_6089	-14.4	GGCCTTTGAAGCTTACTACAGCCTCA
352	hsa_miR_4710	-15.2	GGCTCAAGTGATCCTCAGCC
453	hsa_miR_5787	-20.8	GGCTCAAGTGATCCTCAGCCT
277	hsa_miR_4437	-14.4	GGCTCAAGTGATCCTCAGCCTC
173	hsa_miR_27a_5p	-14.9	GGCTCAAGTGATCCTCAGCCTC
530	hsa_miR_5001_5p	-16.7	GGCTCAAGTGATCCTCAGCCTC
1	hsa_let_7b_5p	-12.6	GGCTCAAGTGATCCTCAGCCTCC
2	hsa_let_7c_5p	-12.6	GGCTCAAGTGATCCTCAGCCTCC
4	hsa_let_7d_5p	-13.2	GGCTCAAGTGATCCTCAGCCTCC
87	hsa_miR_185_3p	-15.3	GGCTCAAGTGATCCTCAGCCTCC
180	hsa_miR_3180_3p	-15.3	GGCTCAAGTGATCCTCAGCCTCC
241	hsa_miR_3197	-18.3	GGCTCAAGTGATCCTCAGCCTCC
465	hsa_miR_4697_5p	-17.8	GGCTCAAGTGATCCTCAGCCTCCC
361	hsa_miR_4728_5p	-20.1	GGCTCAAGTGATCCTCAGCCTCCCA
888	hsa_miR_744_5p	-16.3	GGCTCAAGTGATCCTCAGCCTCCCA
926	hsa_miR_8085	-19.9	GGCTCAAGTGATCCTCAGCCTCCCA
345	hsa_miR_4689	-13.6	GGCTCTGGATAGCACCTTAT
220	hsa_miR_3677_3p	-15.8	GGCTCTGGATAGCACCTTATGGA
374	hsa_miR_4750_5p	-18.1	GGCTTGACTACACTGTGTGGA
617	hsa_miR_6886_5p	-14.1	GGCTTGACTACACTGTGTGGA

906	hsa_miR_7974	-17.9	GGGCTCAAGTGATCCTCAGCCT
161	hsa_miR_2467_5p	-13.9	GGGCTCAAGTGATCCTCAGCCTCC
636	hsa_miR_6089	-22.84	GGGCTCAAGTGATCCTCAGCCTCC
916	hsa_miR_8069	-19.1	GGGCTCAAGTGATCCTCAGCCTCC
195	hsa_miR_345_5p	-13	GGGTAAGGACATGAAGACAGT
656	hsa_miR_7974	-14.94	GGGTAAGGACATGAAGACAGTTC
629	hsa_miR_6073	-15.2	CAAGCATGCAGCTTACTGCT
128	hsa_miR_1972	-12.5	CAAGCATGCAGCTTACTGCTTGA
460	hsa_miR_4692	-15.9	CAAGCATGCAGCTTACTGCTTGA
93	hsa_miR_1469	-14.4	CAAGCATGCAGCTTACTGCTTGAA
409	hsa_miR_4520_5p	-13.4	CACAACAAGCAAGCATGCAGC
212	hsa_miR_3135b	-15.1	CACAACAAGCAAGCATGCAGCT
907	hsa_miR_7974	-13.6	CACAACAAGCAAGCATGCAGCTT
803	hsa_miR_6814_3p	-12.3	CACCCAAGCTAGAGTGCAGT
162	hsa_miR_2467_5p	-16.1	CAGCTTACTGCTTGAAAGGGTCTTG
48	hsa_miR_1279	-12	GGGTGCAGATGCAGTGTGG
126	hsa_miR_23a_3p	-13.2	GGGTGCAGATGCAGTGTGGC
128	hsa_miR_23c	-16.7	GGGTGCAGATGCAGTGTGGC
223	hsa_miR_3683	-12.7	GGGTGCAGATGCAGTGTGGCT
252	hsa_miR_4252	-20	GGGTGCAGATGCAGTGTGGCT
435	hsa_miR_548v	-20.3	GGGTGCAGATGCAGTGTGGCT
477	hsa_miR_621	-13.8	GGGTGCAGATGCAGTGTGGCT
485	hsa_miR_642b_3p	-14.1	GGGTGCAGATGCAGTGTGGCT
659	hsa_miR_8064	-14.2	GGGTGCAGATGCAGTGTGGCT
285	hsa_miR_4467	-12.8	GGGTGCAGATGCAGTGTGGCTC
116	hsa_miR_2114_3p	-16	GGGTGCAGATGCAGTGTGGCTCT
139	hsa_miR_27b_5p	-14.7	GGGTGCAGATGCAGTGTGGCTCT
274	hsa_miR_4424	-12.4	GGGTGCAGATGCAGTGTGGCTCT
314	hsa_miR_4537	-16.9	GGGTGCAGATGCAGTGTGGCTCT
326	hsa_miR_4640_5p	-16	GGGTGCAGATGCAGTGTGGCTCT
541	hsa_miR_6756_5p	-17	GGGTGCAGATGCAGTGTGGCTCT
192	hsa_miR_33b_3p	-21.6	GGGTGCAGATGCAGTGTGGCTCTG
491	hsa_miR_6500_5p	-19.7	GGGTGCAGATGCAGTGTGGCTCTG
526	hsa_miR_6743_5p	-12.2	GGGTGCAGATGCAGTGTGGCTCTG
350	hsa_miR_431_3p	-16.8	GGGTGCAGATGCAGTGTGGCTCTG
55	hsa_miR_1295a	-14.7	GGGTGCAGATGCAGTGTGGCTCTGG
538	hsa_miR_6754_5p	-14.7	GGGTGCAGATGCAGTGTGGCTCTGG
648	hsa_miR_764	-13.1	GGGTGCAGATGCAGTGTGGCTCTGG
663	hsa_miR_8074	-19.9	GGGTGCAGATGCAGTGTGGCTCTGG
665	hsa_miR_8078	-16.2	GGGTGCAGATGCAGTGTGGCTCTGGAT[illegible]
117	hsa_miR_212_3p	-13.3	GGTAAGGACATGAAGACAGTTC
383	hsa_miR_4782_3p	-16.3	GGTAAGGACATGAAGACAGTTC
367	hsa_miR_4741	-13	GGTAAGGACATGAAGACAGTTCC
28	hsa_miR_1204	-12.4	GGTATGGAGAGTCGGCTTGA
329	hsa_miR_4650_5p	-15.5	GGTATGGAGAGTCGGCTTGA
200	hsa_miR_3617_3p	-16.9	GGTATGGTGCTGGGTGCAGATG
353	hsa_miR_4711_5p	-14.9	GGTATGGTGCTGGGTGCAGATGCA

573	hsa_miR_6805_3p	-21.7	GGTATGGTGCTGGGTGCAGATGCAG
637	hsa_miR_6089	-15.9	CAGCTTACTGCTTGAAAGGGTCTTG
750	hsa_miR_6762_5p	-16.3	CAGCTTACTGCTTGAAAGGGTCTTG
497	hsa_miR_4750_5p	-15.4	CATGCAGCTTACTGCTTGAA
189	hsa_miR_30a_3p	-12.1	CATGCAGCTTACTGCTTGAAAG
182	hsa_miR_297	-13.2	CCACAACAAGCAAGCATGCAG
600	hsa_miR_5589_5p	-18.6	CCACAACAAGCAAGCATGCAGCT
611	hsa_miR_574_5p	-15.9	CCACAACAAGCAAGCATGCAGCTTA
127	hsa_miR_23b_3p	-12.9	GGTGCAGATGCAGTGTGGC
673	hsa_miR_891a_3p	-12.9	GGTGCAGATGCAGTGTGGCT
297	hsa_miR_449b_3p	-18	GGTGCAGATGCAGTGTGGCTC
137	hsa_miR_27a_5p	-16.8	GGTGCAGATGCAGTGTGGCTCT
385	hsa_miR_4786_5p	-19.2	GGTGCAGATGCAGTGTGGCTCT
498	hsa_miR_6508_3p	-14.9	GGTGCAGATGCAGTGTGGCTCT
321	hsa_miR_4632_5p	-12.4	GGTGCAGATGCAGTGTGGCTCTG
377	hsa_miR_4761_3p	-15.3	GGTGCAGATGCAGTGTGGCTCTG
465	hsa_miR_6081	-19	GGTGCAGATGCAGTGTGGCTCTG
474	hsa_miR_615_5p	-14	GGTGCAGATGCAGTGTGGCTCTG
642	hsa_miR_7160_3p	-16.9	GGTGCAGATGCAGTGTGGCTCTG
181	hsa_miR_3187_5p	-14	GGTGCAGATGCAGTGTGGCTCTGG
492	hsa_miR_6501_3p	-20.7	GGTGCAGATGCAGTGTGGCTCTGG
666	hsa_miR_8081	-14.2	GGTGCAGATGCAGTGTGGCTCTGG
73	hsa_miR_1469	-16.9	GGTGCAGATGCAGTGTGGCTCTGGA
87	hsa_miR_139_5p	-12.7	GGTGCAGATGCAGTGTGGCTCTGGA
143	hsa_miR_297	-15	GGTGCTGGGTGCAGATGCAG
585	hsa_miR_6818_5p	-13.9	GGTGCTGGGTGCAGATGCAG
312	hsa_miR_4526	-12.6	GGTGCTGGGTGCAGATGCAGT
365	hsa_miR_4734	-24.3	GGTGCTGGGTGCAGATGCAGT
120	hsa_miR_217	-13.7	GGTGCTGGGTGCAGATGCAGTG
210	hsa_miR_3650	-13.1	GGTGCTGGGTGCAGATGCAGTG
216	hsa_miR_3664_5p	-12	GGTGCTGGGTGCAGATGCAGTG
677	hsa_miR_892c_3p	-12.6	GGTGCTGGGTGCAGATGCAGTG
681	hsa_miR_92b_3p	-20.8	GGTGCTGGGTGCAGATGCAGTG
449	hsa_miR_4676_3p	-12.2	GGTGCTGGGTGCAGATGCAGTG
99	hsa_miR_1915_5p	-19.4	GGTGCTGGGTGCAGATGCAGTGT
348	hsa_miR_4693_5p	-12.2	GGTGCTGGGTGCAGATGCAGTGT
486	hsa_miR_643	-12.1	GGTGCTGGGTGCAGATGCAGTGT
339	hsa_miR_4675	-17.7	GGTGCTGGGTGCAGATGCAGTGTG
510	hsa_miR_6720_3p	-15.5	GGTGCTGGGTGCAGATGCAGTGTG
101	hsa_miR_195_3p	-17.3	GGTGCTGGGTGCAGATGCAGTGTGG
122	hsa_miR_221_3p	-20.7	GGTGCTGGGTGCAGATGCAGTGTGGCT
582	hsa_miR_6816_5p	-13.8	GTAAGGACATGAAGACAGTTCCT
251	hsa_miR_424_5p	-12.2	GTAAGGACATGAAGACAGTTCCTG
658	hsa_miR_8059	-13	GTAAGGACATGAAGACAGTTCCTG
215	hsa_miR_3139	-13	GTAAGGACATGAAGACAGTTCCTG
30	hsa_miR_1207_3p	-14.8	GTATGGAGAGTCGGCTTGA
389	hsa_miR_4793_5p	-16.1	GTATGGTGCTGGGTGCAGATGC

624	hsa_miR_6895_3p	-15.7	GTATGGTGCTGGGTGCAGATGCA
102	hsa_miR_1587	-19.8	CCTCACCCAAGCTAGAGTGCAG
387	hsa_miR_4792	-13.2	GTCGGCTTGACTACACTG
37	hsa_miR_1250_5p	-13	GTCGGCTTGACTACACTGT
346	hsa_miR_4302	-14.8	GTCGGCTTGACTACACTGT
135	hsa_miR_2682_5p	-14.4	GTCGGCTTGACTACACTGTGTG
550	hsa_miR_6768_5p	-16.6	GTCGGCTTGACTACACTGTGTG
912	hsa_miR_8064	-19.2	GTCGGCTTGACTACACTGTGTG
347	hsa_miR_4692	-17.92	GTCGGCTTGACTACACTGTGTGG
900	hsa_miR_7703	-20.16	CCTCACCCAAGCTAGAGTGCAG
150	hsa_miR_221_3p	-13.5	CTCACCCAAGCTAGAGTGCAGTG
793	hsa_miR_6807_3p	-25.8	CTCACCCAAGCTAGAGTGCAGTG
867	hsa_miR_7108_5p	-16.3	CTGCTTGAAAGGGTCTTGCCTCAC
440	hsa_miR_4661_3p	-15.9	CTTACTGCTTGAAAGGGTCTTG
866	hsa_miR_7108_5p	-15.1	CTTACTGCTTGAAAGGGTCTTGC
289	hsa_miR_4482_5p	-13.4	GTGAGGTATGGTGCTGGGTG
552	hsa_miR_6773_5p	-14.3	GTGAGGTATGGTGCTGGGTGCAG
384	hsa_miR_4784	-14.3	GTGATCCTCAGCCTCCCA
570	hsa_miR_6799_5p	-16.7	GTGATCCTCAGCCTCCCA
612	hsa_miR_6870_5p	-18.8	GTGATCCTCAGCCTCCCA
261	hsa_miR_4298	-20.4	GTGATCCTCAGCCTCCCAG
555	hsa_miR_6779_5p	-23.4	GTGATCCTCAGCCTCCCAG
113	hsa_miR_204_3p	-21.2	GTGATCCTCAGCCTCCCAGT
115	hsa_miR_211_3p	-12.3	GTGATCCTCAGCCTCCCAGT
84	hsa_miR_181d_3p	-18.9	GTGATCCTCAGCCTCCCAGTGG
123	hsa_miR_222_3p	-14.1	GTGCAGATGCAGTGTGGCT
246	hsa_miR_409_5p	-14.3	GTGCAGATGCAGTGTGGCTC
370	hsa_miR_4743_5p	-13.1	GTGCAGATGCAGTGTGGCTC
527	hsa_miR_6744_3p	-17.8	GTGCAGATGCAGTGTGGCTC
291	hsa_miR_4487	-14.9	GTGCAGATGCAGTGTGGCTCT
295	hsa_miR_4498	-14.1	GTGCAGATGCAGTGTGGCTCT
470	hsa_miR_610	-14.4	GTGCAGATGCAGTGTGGCTCT
307	hsa_miR_4512	-26.2	GTGCAGATGCAGTGTGGCTCTG
403	hsa_miR_503_5p	-13.5	GTGCAGATGCAGTGTGGCTCTG
490	hsa_miR_650	-12	GTGCAGATGCAGTGTGGCTCTG
545	hsa_miR_6762_5p	-22.9	GTGCAGATGCAGTGTGGCTCTG
14	hsa_miR_105_3p	-12.3	GTGCAGATGCAGTGTGGCTCTGG
426	hsa_miR_520g_5p	-19.7	GTGCAGATGCAGTGTGGCTCTGGA
685	hsa_miR_939_3p	-16.1	GTGCAGATGCAGTGTGGCTCTGGA
304	hsa_miR_450a_1_	-14	GTGCAGATGCAGTGTGGCTCTGGAT
561	hsa_miR_6783_3p	-17	GTGCAGATGCAGTGTGGCTCTGGAT
34	hsa_miR_1231	-15.7	GTGCTGGGTGCAGATGC
269	hsa_miR_4325	-14.6	GTGCTGGGTGCAGATGCAG
161	hsa_miR_3130_3p	-14.3	GTGCTGGGTGCAGATGCAGT
580	hsa_miR_6814_3p	-15.4	GTGCTGGGTGCAGATGCAGT
63	hsa_miR_130a_3p	-12.4	GTGCTGGGTGCAGATGCAGTG
272	hsa_miR_4418	-22.3	GTGCTGGGTGCAGATGCAGTG

619	hsa_miR_6890_3p	-19.9	GTGCTGGGTGCAGATGCAGTGT
52	hsa_miR_1288_3p	-13.3	GTGCTGGGTGCAGATGCAGTGTG
441	hsa_miR_5580_3p	-17.2	GTGCTGGGTGCAGATGCAGTGTG
571	hsa_miR_6804_3p	-22.9	GTGCTGGGTGCAGATGCAGTGTG
16	hsa_miR_106b_3p	-22	GTGCTGGGTGCAGATGCAGTGTGG
294	hsa_miR_4494	-20.8	GTGCTGGGTGCAGATGCAGTGTGG
549	hsa_miR_6767_3p	-17.1	GTGCTGGGTGCAGATGCAGTGTGG
622	hsa_miR_6893_3p	-15.3	GTGCTGGGTGCAGATGCAGTGTGG
499	hsa_miR_6509_3p	-13.4	GTGCTGGGTGCAGATGCAGTGTGGC
90	hsa_miR_187_5p	-18.9	GTGCTGGGTGCAGATGCAGTGTGGCT
509	hsa_miR_668_3p	-18.6	GTGCTGGGTGCAGATGCAGTGTGGCT
530	hsa_miR_6746_3p	-18.8	GTGCTGGGTGCAGATGCAGTGTGGCTC
404	hsa_miR_504_3p	-13.9	GTGGCTCTGGATAGCACCTT
65	hsa_miR_1343_5p	-12.6	GTGGCTCTGGATAGCACCTTA
194	hsa_miR_342_5p	-19.2	GTGGCTCTGGATAGCACCTTA
366	hsa_miR_4735_3p	-12.8	GTGGCTCTGGATAGCACCTTA
332	hsa_miR_4658	-14.6	GTGGCTCTGGATAGCACCTTAT
170	hsa_miR_3154	-13.4	GTGGCTCTGGATAGCACCTTATG
620	hsa_miR_6890_5p	-14.7	GTGGCTCTGGATAGCACCTTATG
343	hsa_miR_4687_3p	-15.1	GTGTGGCTCTGGATAGCACCT
647	hsa_miR_762	-24.9	GTGTGGCTCTGGATAGCACCT
165	hsa_miR_3139	-15.9	GTGTGGCTCTGGATAGCACCTT
342	hsa_miR_4678	-15.3	GTGTGGCTCTGGATAGCACCTT
551	hsa_miR_6772_5p	-19.7	GTGTGGCTCTGGATAGCACCTT
91	hsa_miR_18a_5p	-21.7	GTGTGGCTCTGGATAGCACCTTA
598	hsa_miR_6842_5p	-16.7	GTGTGGCTCTGGATAGCACCTTA
600	hsa_miR_6846_5p	-15.4	GTGTGGCTCTGGATAGCACCTTA
79	hsa_miR_154_5p	-15.3	GTTTTTATCCATGGATGACTTG
145	hsa_miR_302d_3p	-12.7	GTTTTTATCCATGGATGACTTG
75	hsa_miR_148a_5p	-12.1	TAAAACAGTGTCTCCAATTTT
536	hsa_miR_5007_5p	-12.1	TAACCAATGATGTAATGATTCTG
107	hsa_miR_199a_3p	-17.2	TAACCAATGATGTAATGATTCTGC
109	hsa_miR_199b_3p	-17.2	TAACCAATGATGTAATGATTCTGC
74	hsa_miR_147b	-13.1	TAACTCAAGCCATTGGCACAC
627	hsa_miR_7108_5p	-16.2	TAACTCAAGCCATTGGCACACAG
505	hsa_miR_661	-20.8	TAACTCAAGCCATTGGCACACAGGCA
235	hsa_miR_3180	-13.6	CTTGAAAGGGTCTTGCCTCA
379	hsa_miR_4474_3p	-16.5	CTTGCCTCACCCAAGCTAGAG
69	hsa_miR_1293	-13.5	GAAAGGGTCTTGCCTCACCCA
761	hsa_miR_6772_5p	-21.8	GAAAGGGTCTTGCCTCACCCA
157	hsa_miR_3126_3p	-19.3	TATGGTGCTGGGTGCAGATG
632	hsa_miR_7112_3p	-17.3	TATGGTGCTGGGTGCAGATGCA
653	hsa_miR_7703	-15.7	TATGGTGCTGGGTGCAGATGCAG
380	hsa_miR_4769_3p	-12.1	TATGGTGCTGGGTGCAGATGCAGT
381	hsa_miR_4772_3p	-13.2	TATGGTGCTGGGTGCAGATGCAGT
519	hsa_miR_6737_3p	-16.4	TATGGTGCTGGGTGCAGATGCAGT
606	hsa_miR_6861_5p	-13	TATGGTGCTGGGTGCAGATGCAGT

507	hsa_miR_664a_3p	-15.7	TATGGTGCTGGGTGCAGATGCAGTG
553	hsa_miR_6776_5p	-14.1	TCAAGCCATTGGCACACAGG
566	hsa_miR_6788_5p	-15.8	TCAAGTGATCCTCAGCCTCCCAG
734	hsa_miR_6751_5p	-16.5	TCAAGTGATCCTCAGCCTCCCAG
636	hsa_miR_7114_5p	-13.9	TCAAGTGATCCTCAGCCTCCCAGT
254	hsa_miR_4259	-16	TCAAGTGATCCTCAGCCTCCCAGTG
710	hsa_miR_6736_5p	-17.9	GAAAGGGTCTTGCCTCACCCAA
812	hsa_miR_6821_5p	-13.8	GACCACAACAAGCAAGCATGCAG
515	hsa_miR_4792	-18.4	GCAAGCATGCAGCTTACTG
391	hsa_miR_449a	-16.3	GCAAGCATGCAGCTTACTGCTT
270	hsa_miR_3619_5p	-20.3	GCAAGCATGCAGCTTACTGCTTGA
193	hsa_miR_30d_3p	-16.4	GCAGCTTACTGCTTGAAAG
655	hsa_miR_630	-14.7	GCCTCACCCAAGCTAGAGTGCA
256	hsa_miR_33b_5p	-13.4	TCAGTGTAACCAATGATGTAA
663	hsa_miR_639	-12	TCAGTGTAACCAATGATGTAATGAT
118	hsa_miR_215_3p	-14.2	TCATGATTGAAGAAATGCAGG
460	hsa_miR_6068	-14.6	TCATGATTGAAGAAATGCAGG
371	hsa_miR_4746_3p	-18.3	TCGGCTTGACTACACTGTG
19	hsa_miR_106b_3p	-19.2	TCGGCTTGACTACACTGTGTGG
435	hsa_miR_4656	-20.7	TCTGGGCTCAAGTGATCCTCAGCCTC
455	hsa_miR_4687_3p	-13	GCTTACTGCTTGAAAGGGTCT
909	hsa_miR_8052	-17.9	GCTTACTGCTTGAAAGGGTCTTG
860	hsa_miR_6893_5p	-14.02	GCTTGAAAGGGTCTTGCCTC
305	hsa_miR_3689d	-14.5	GCTTGAAAGGGTCTTGCCTCACC
247	hsa_miR_328_5p	-22.8	GCTTGAAAGGGTCTTGCCTCACCC
559	hsa_miR_518e_5p	-13.3	GGGTCTTGCCTCACCCAAGCTAGAG
567	hsa_miR_519a_5p	-13.3	GGGTCTTGCCTCACCCAAGCTAGAG
569	hsa_miR_519b_5p	-13.3	GGGTCTTGCCTCACCCAAGCTAGAG
571	hsa_miR_519c_5p	-13.3	GGGTCTTGCCTCACCCAAGCTAGAG
275	hsa_miR_4430	-19.4	TGAAGCTTACTACAGCCT
503	hsa_miR_656_5p	-12.5	TGAAGCTTACTACAGCCT
9	hsa_let_7i_5p	-17.2	TGAAGCTTACTACAGCCTCA
452	hsa_miR_574_5p	-13	TGAAGCTTACTACAGCCTCA
572	hsa_miR_6804_5p	-14.1	TGAAGCTTACTACAGCCTCA
678	hsa_miR_920	-16.9	TGAAGCTTACTACAGCCTCA
81	hsa_miR_1587	-13.4	TGAAGCTTACTACAGCCTCAA
523	hsa_miR_6741_5p	-14.2	TGAAGCTTACTACAGCCTCAA
603	hsa_miR_6857_5p	-12.4	TGAAGCTTACTACAGCCTCAA
60	hsa_miR_1304_5p	-18.5	TGAAGCTTACTACAGCCTCAAA
576	hsa_miR_522_5p	-13.3	GGGTCTTGCCTCACCCAAGCTAGAG
578	hsa_miR_523_5p	-13.3	GGGTCTTGCCTCACCCAAGCTAGAG
447	hsa_miR_5691	-14.3	TGAGGTATGGTGCTGGGTGCAG
164	hsa_miR_3137	-15	TGAGGTATGGTGCTGGGTGCAGA
213	hsa_miR_3656	-14.3	TGATCCTCAGCCTCCC
231	hsa_miR_3178	-14.8	TGATCCTCAGCCTCCC
360	hsa_miR_4727_3p	-17.2	TGATCCTCAGCCTCCCAGTGG
112	hsa_miR_202_3p	-12.3	TGCAGATGCAGTGTGGCTCT

219	hsa_miR_3670	-17	TGCAGATGCAGTGTGGCTCT
83	hsa_miR_15a_5p	-12.1	TGCAGATGCAGTGTGGCTCTG
322	hsa_miR_4633_3p	-18.2	TGCAGATGCAGTGTGGCTCTG
400	hsa_miR_5007_5p	-12.5	TGCAGATGCAGTGTGGCTCTG
411	hsa_miR_5090	-14.7	TGCAGATGCAGTGTGGCTCTGG
396	hsa_miR_498	-16	TGCAGATGCAGTGTGGCTCTGGAT
590	hsa_miR_5572	-14.9	GGTCTTGCCTCACCCAAGC
303	hsa_miR_4508	-14.8	TGCAGTGTGGCTCTGG
191	hsa_miR_33a_5p	-20.3	TGCAGTGTGGCTCTGGATAGCAC
397	hsa_miR_5001_5p	-19.2	TGCAGTGTGGCTCTGGATAGCACCT
424	hsa_miR_4633_3p	-21.8	TGCAGTGTGGCTCTGGATAGCACCT
485	hsa_miR_4741	-21.52	TGCAGTGTGGCTCTGGATAGCACCT
344	hsa_miR_4688	-14.7	TGCAGTGTGGCTCTGGATAGCACCTTA
716	hsa_miR_6740_5p	-16.5	GGTCTTGCCTCACCCAAGCT
565	hsa_miR_5195_3p	-12.1	GGTCTTGCCTCACCCAAGCTAGAG
697	hsa_miR_6720_5p	-14	GGTCTTGCCTCACCCAAGCTAGAG
233	hsa_miR_378j	-17.4	TGCTGGGTGCAGATGCAGT
58	hsa_miR_1301_5p	-19.7	TGCTGGGTGCAGATGCAGTG
154	hsa_miR_3120_3p	-14.1	TGCTGGGTGCAGATGCAGTG
267	hsa_miR_4318	-14	TGCTGGGTGCAGATGCAGTG
357	hsa_miR_4718	-17.7	TGCTGGGTGCAGATGCAGTG
597	hsa_miR_6841_3p	-24.1	TGCTGGGTGCAGATGCAGTGT
177	hsa_miR_3177_3p	-13.1	TGCTGGGTGCAGATGCAGTGTG
3	hsa_let_7d_3p	-14	TGCTGGGTGCAGATGCAGTGTGG
654	hsa_miR_7848_3p	-16.4	TGCTGGGTGCAGATGCAGTGTGG
184	hsa_miR_3197	-15.5	TGCTGGGTGCAGATGCAGTGTGGCTCT
614	hsa_miR_5787	-17.6	GTCTTGCCTCACCCAAGCTA
498	hsa_miR_4750_5p	-14	GTCTTGCCTCACCCAAGCTAGAG
689	hsa_miR_663a	-18.5	TACTGCTTGAAAGGGTCTTGCCT
356	hsa_miR_4325	-12.6	TCACCCAAGCTAGAGTGCAG
209	hsa_miR_3130_3p	-20.1	TCACCCAAGCTAGAGTGCAGT
260	hsa_miR_345_5p	-15.2	TCACCCAAGCTAGAGTGCAGT
166	hsa_miR_25_3p	-16.9	TCACCCAAGCTAGAGTGCAGTG
401	hsa_miR_4507	-20.9	TCTTGCCTCACCCAAGCTAG
753	hsa_miR_6765_3p	-12.1	TCTTGCCTCACCCAAGCTAGAGTGC
257	hsa_miR_33b_5p	-15.7	TGACCACAACAAGCAAGCAT
639	hsa_miR_7152_5p	-12.9	TGCTTGGGTAAGGACATGAAG
257	hsa_miR_4268	-13.6	TGGAAGTTGAAATGGTGGAGTC
54	hsa_miR_1293	-12.7	TGGAGAGTCGGCTTGACTACACTG
146	hsa_miR_3064_5p	-18	TGGCACACAGGCATTAGACAGA
495	hsa_miR_6504_5p	-12.8	TGGCACACAGGCATTAGACAGA
200	hsa_miR_3120_5p	-21.3	TGGCACACAGGCATTAGACAGA
283	hsa_miR_3650	-17.2	TGGCTCTGGATAGCACCT
178	hsa_miR_3178	-14.2	TGGCTCTGGATAGCACCTT
590	hsa_miR_6829_5p	-14.9	TGGCTCTGGATAGCACCTT
722	hsa_miR_6743_5p	-12.4	TGGCTCTGGATAGCACCTT
23	hsa_miR_1178_5p	-16.3	TGGGCTCAAGTGATCCTC

62	hsa_miR_1286	-12.3	TGGGCTCAAGTGATCCTCA
364	hsa_miR_4731_5p	-23.8	TGGGCTCAAGTGATCCTCAGCC
445	hsa_miR_5589_5p	-18.3	TGGGCTCAAGTGATCCTCAGCC
516	hsa_miR_6729_5p	-21	TGGGCTCAAGTGATCCTCAGCCTC
447	hsa_miR_4675	-16.2	TGGGCTCAAGTGATCCTCAGCCTC
341	hsa_miR_4677_5p	-12.8	TGGGTAAGGACATGAAGACAG
489	hsa_miR_4746_3p	-15.5	TGGGTGCAGATGCAGTGTG
59	hsa_miR_1304_3p	-16.1	TGGGTGCAGATGCAGTGTGG
206	hsa_miR_3622b_3	-13.5	TGGGTGCAGATGCAGTGTGG
392	hsa_miR_485_3p	-14	TGGGTGCAGATGCAGTGTGGC
407	hsa_miR_505_3p	-12.9	TGGGTGCAGATGCAGTGTGGCT
525	hsa_miR_6743_3p	-19.3	TGGGTGCAGATGCAGTGTGGCT
624	hsa_miR_6069	-19.3	TGGGTGCAGATGCAGTGTGGCTC
483	hsa_miR_638	-17.7	TGGGTGCAGATGCAGTGTGGCTCT
53	hsa_miR_1289	-20	TGGGTGCAGATGCAGTGTGGCTCTG
82	hsa_miR_15a_3p	-28.1	TGGGTGCAGATGCAGTGTGGCTCTG
173	hsa_miR_3158_3p	-15.6	TGGGTGCAGATGCAGTGTGGCTCTG
350	hsa_miR_4697_5p	-14.2	TGGGTGCAGATGCAGTGTGGCTCTG
443	hsa_miR_5586_3p	-13.6	TGGGTGCAGATGCAGTGTGGCTCTG
106	hsa_miR_1972	-17.5	TGGGTGCAGATGCAGTGTGGCTCTGG
432	hsa_miR_4650_5p	-12.5	TGGGTGCAGATGCAGTGTGGCTCTGG
413	hsa_miR_5100	-16.4	TGGGTGCAGATGCAGTGTGGCTCTGGA
480	hsa_miR_632	-14.8	TGGTGCTGGGTGCAGATGC
133	hsa_miR_25_3p	-17.9	TGGTGCTGGGTGCAGATGCAGTG
208	hsa_miR_363_3p	-14.4	TGGTGCTGGGTGCAGATGCAGTG
675	hsa_miR_892a	-13.3	TGGTGCTGGGTGCAGATGCAGTG
171	hsa_miR_3156_5p	-13.7	TGTCTCCAATTTTAATAAATTTTT
607	hsa_miR_6862_3p	-17	TGTGAGGTATGGTGCTGGGTGCAGA
467	hsa_miR_6085	-17.3	TGTGGCTCTGGATAGCACCTT
17	hsa_miR_106b_5p	-13.4	TGTGGCTCTGGATAGCACCTTA
694	hsa_miR_668_3p	-13.3	TGCCTCACCCAAGCTAGAGTGCA
7	hsa_let_7f_5p	-12.2	TGCTTGAAAGGGTCTTGCCTCA
233	hsa_miR_3180_3p	-15.9	TGCTTGAAAGGGTCTTGCCTCA
69	hsa_miR_141_5p	-13.8	TTAGACAGAAAGCTGGAAGTTG
643	hsa_miR_7160_5p	-15.8	TTCTGGGCTCAAGTGATCCTCAGCC
242	hsa_miR_3197	-21.9	TGCTTGAAAGGGTCTTGCCTCA
539	hsa_miR_5008_5p	-18.1	TTACTGCTTGAAAGGGTCTTG
699	hsa_miR_6721_5p	-16.5	TTACTGCTTGAAAGGGTCTTGCCTC
327	hsa_miR_3975	-13.3	TTGAAAGGGTCTTGCCTCA
705	hsa_miR_6729_5p	-15.6	TTGAAAGGGTCTTGCCTCACCCA
708	hsa_miR_6731_5p	-13.6	TTGAAAGGGTCTTGCCTCACCCA
817	hsa_miR_6825_5p	-17.1	TTGAAAGGGTCTTGCCTCACCCA
245	hsa_miR_3975	-13.53	TTGAAGCTTACTACAGCCTCA
186	hsa_miR_3064_5p	-18.6	TTGCCTCACCCAAGCTAGA
521	hsa_miR_6740_3p	-17.9	TTGCTTGGGTAAGGACATGAAGACA
591	hsa_miR_6830_3p	-13.1	TTGCTTGGGTAAGGACATGAAGACA
464	hsa_miR_6080	-13.5	TTGGCACACAGGCATTAGA

688	hsa_miR_943	-13.1	TTGGCACACAGGCATTAGACAG
89	hsa_miR_187_3p	-14.9	TTGGCACACAGGCATTAGACAGA
618	hsa_miR_6888_3p	-12.5	TTGGCACACAGGCATTAGACAGAA
369	hsa_miR_4743_3p	-18.3	TTGGCACACAGGCATTAGACAGAAA
584	hsa_miR_6818_3p	-12.3	TTGGGTAAGGACATGAAGACAG
327	hsa_miR_4646_3p	-14.8	TTGGGTAAGGACATGAAGACAGT
712	hsa_miR_6737_3p	-18.9	TTGGGTAAGGACATGAAGACAGT
613	hsa_miR_6876_3p	-18.5	TTGGGTAAGGACATGAAGACAGTT
308	hsa_miR_4513	-12.1	TTGGGTAAGGACATGAAGACAGTTC
689	hsa_miR_99a_3p	-13.8	TTTATCCATGGATGACTTG
690	hsa_miR_99b_3p	-13.3	TTTATCCATGGATGACTTG
657	hsa_miR_8052	-15.4	TTTGAAGCTTACTACAGCCTC
446	hsa_miR_4675	-13.4	TTTGAAGCTTACTACAGCCTC
6	hsa_let_7f_5p	-13	TTTGAAGCTTACTACAGCCTCA
8	hsa_let_7g_5p	-16.2	TTTGAAGCTTACTACAGCCTCA
292	hsa_miR_4489	-14.4	TTTGAAGCTTACTACAGCCTCA
630	hsa_miR_7110_5p	-16	TTTGAAGCTTACTACAGCCTCA
352	hsa_miR_431_3p	-13.9	TTTTATCCATGGATGACTTG
124	hsa_miR_195_3p	-12.3	TTTTCAGTGTAACCAATGATGT
215	hsa_miR_3663_5p	-12.4	TTTTTATCCATGGATGACTTGC

miRNA sequence	Number of	Length of h	P value
CTGGGGAGATCCTCGAGGTTGG	17	23	0.35
AGCTTCTTTACAGTGCTGCCTTG	18	24	0.275
CGGGGCCGTAGCACTGTCTGAGA	14	23	0.275
GCTATTTCACGACACCAGGGTT	14	21	0.203
CGGGGCCGTAGCACTGTCTGAGA	14	22	0.0648
TGGCAGTTACTTTTGCACCAG	13	20	0.111
ACGGGTTAGGCTCTTGGGAGCT	13	21	0.332
CAGGCACGGGAGCTCAGGTGAG	15	22	0.0344
CTCCTCTGTTTTCTTTCCTAG	18	21	0.203
CTGGGAGGTGTGATATCGTGGT	16	25	0.111
TGAGGCAGTAGATTGAAT	12	16	0.0344
ATTGGGGACATTTTGCATTCAT	12	22	0.0866
GGGTGGGGATTTGTTGCATTAC	13	22	0.143
GGTGAGGCTAGCTGGTG	12	18	0.0344
TGGGGAAGGCTTGGCAGGGAAGA	13	24	0.143
CATGGTTCTGTCAAGCACCGCG	16	21	0.332
GGTGGTTGAGGCTGCAGTAAGT	16	22	0.143
AGGCATGGGAGGTCAGGTGA	16	18	0.143
TTGGGAGGGAAGACAGCTGGAGA	18	25	0.143
CTGGGAGGTGTGATATCGTGGT	16	25	0.143
CAGGCAGAAGTGGGGCTGACAGG	17	25	0.332
CTGGGAGGTGTGATATTGTGGT	18	25	0.143
CTGGGAGGTCAAGGCTGCAGT	15	20	0.143
TTGGAGGCGTGGGTTTT	14	16	0.0866
GGTGGGGGGTGTTGTTTT	15	18	0.0866
TTGGAATAGGGGATATCTCAGC	15	22	0.0866
GGCTGAGTGGGGTTCTGACTCC	13	23	0.203
TCGTATCAGAGATTCCAGACAC	14	20	0.203
TGGGATGAGGGATTGAAGTGGA	12	20	0.0344
AAAATGGTGCCCTAGTGACTACA	12	23	0.0104
AAGTTGGCTGCAGTTAAGGTGG	15	21	0.0344
CCAGTGGGGCTGCTGTTATCTG	15	22	0.0344
CGCAGGGGCCGGGTGCTCACCG	14	23	0.0344
AGAAGTAATTGCGGTTTTGCCA	17	23	0.0344
AAGTAGTTGGTTTGTATGAGATGGTT	20	24	0.0344
TTCGGGCTGGCCTGCTGCTCCGG	12	24	0.332
CGGGTAGAGAGGGCAGTGGGAGG	16	25	0.0344
ACGGGCAGGGCAGTGCACCCTG	14	26	0.0344
GAGGCAGAAGCAGGATGACA	14	21	0.0344
GCAGGACAGGCAGAAGTGGAT	14	23	0.111
GGGGGGATGTGCATGCTGGTT	17	24	0.111
CGGCGGGGACGGCGATTGGTC	17	25	0.111
TCGTACCGTGAGTAATAATGCG	16	21	0.284

CGGGCATGCTGGGAGAGACTTT	18	25	0.284
TGAGTGCCGGTGCCTGCCCTG	13	21	0.203
TGGGGAGGTGTGGAGTCAGCAT	14	24	0.0866
GTTGGGACAAGAGGACGGTCTT	15	23	0.0866
TGGGCGTATCTGTATGCTA	16	21	0.0344
TGGCAGTGTCTTAGCTGGTTGT	18	22	0.0344
CAGCAGCACACTGTGGTTTGT	14	19	0.0344
CGGGAGCTGGGGTCTGCAGGT	16	23	0.0344
TTGCAGCTGCGGTTGTAAGGT	18	22	0.0344
CTGGGTGAGGGCATCTGTGGT	14	22	0.0344
TGCAGGACCAAGATGAGCCCT	12	21	0.0344
GTGAGTAGTGGCGCGCGGCGGC	18	25	0.0344
TAGTGAGTTAGAGATGCAGAGCC	16	20	0.0344
AGCAGAGGCAGAGAGGCTCAGG	13	22	0.0344
AGGCAGCGGGGTGTAGTGGATA	14	23	0.0344
AGCCTGTGCTTGTCCCTGCAG	13	20	0.203
TAGAGTCTGGCTGATATGGTTT	17	23	0.203
GGATGGTTGGGGGCGGTCGGCGT	15	23	0.203
CGGTGGACTGGAGTGGGTGG	16	20	0.203
CCAGTGACTGAGCTGGAGCCA	16	21	0.203
ATCAGGGCTTGTGGAATGGGAAG	15	22	0.203
AGTCATTGGAGGGTTTGAGCAG	15	23	0.203
TGAGCCGAGCTGAGCTTAGCTG	19	25	0.203
CCCAGTGTTCAGACTACCTGTTC	16	23	0.203
TTCGCCACTTCCCTCCCTGCAG	16	23	0.203
GCTGCACCGGAGACTGGGTAA	15	25	0.203
GGGGCCTGGCGGTGGGCGG	14	17	0.0648
TGGGGCTCAGCGAGTTT	14	18	0.0648
TGAGGTAGTAGTTTCTT	14	18	0.0648
TAAGGGGTGTATGGCAGATGCA	14	24	0.0344
CAGGGAGGCGCTCACTCTCTGCT	17	24	0.0344
TGGGCAGGGGCTTATTGTAGGAG	14	24	0.0344
AGACCCTGGTCTGCACTCTATC	12	22	0.0344
TGAGACCTCTGGGTTCTGAGCT	18	24	0.0344
CTTGGGGCATGGAGTCCCA	13	20	0.0344
GGTGGGGCAATGGGATCAGGT	14	19	0.143
TGGGGGAGTGCAGTGATTGTGG	14	20	0.143
GTTGGGACAAGAGGACGGTCTT	14	22	0.143
TCTGAGAGAGCTCGATGGCAG	14	22	0.143
ACTGGGTAGGTGGGGCTCCAGG	16	22	0.143
TGAGGCTCTGTTAGCCTTGGCTC	14	21	0.203
AGCTCGGTCTGAGGCCCCTCAGT	12	22	0.0344
CTGGCGGAGCCCATTCCATGCCA	15	22	0.111
GAGCTTGGATGAGCTGGGCTGA	17	23	0.0344
TCTGGATTGAAGAGACGACCCA	16	25	0.0344
TGGAGAGAAAGGCAGTTCCTGA	18	23	0.0104
TTGGGGGTGAGGTTGGTGTCTGG	18	24	0.0104

GCGGAAGGCGGAGCGGCGGA	13	19	0.0344
CAGGGAGAAGGTGGAAGTGCAGA	15	22	0.0344
ATTAGGTAGTGGCAGTGGAAC	15	24	0.0344
TCCCTGTCCTCCAGGAGCTCACG	16	25	0.0344
TTCAGCCAGGCTAGTGCAGTCT	16	26	0.203
TCATAGCCCTGTACAATGCTGCT	16	24	0.203
TTCCAGCCCTTCTAATGGTAGG	17	25	0.203
TTCCAGCCCTGGTAGGCGCCGCG	16	25	0.203
GGCGGAGGGAAGTAGGTCCGTTGGT	18	23	0.111
TGGCAGTGTATTGTTAGCTGGT	18	24	0.111
TGGGAGAGCAGGGTATTGTGGA	16	24	0.111
TCAGTGCATGACAGAACTTGG	14	19	0.0344
TGTCTCTGCTGGGGTTTCT	14	21	0.0344
ACTGGGCAGGGCTGTGGTGAGT	16	23	0.0344
TGCAGCTCTGGTGGAAAATGGAG	14	20	0.0344
CTGAACTAGAGATTGGGCCCA	13	21	0.0344
AGTCATTGGAGGGTTTGAGCAG	16	22	0.284
TTGTGTCAATATGCGATGATGT	16	19	0.203
CTGTGGGCTCAGCGCGTGGGG	16	21	0.203
CGCTTTGCTCAGCCAGTGTAG	17	23	0.0344
GTGTGACCACCGTTCCTGCAG	14	19	0.0344
CGCGCGGCCGTGCTCGGAGCAG	16	20	0.0344
CGCAGGGCCCTGGCGCAGGCAT	16	20	0.0344
TGGCAGGGAGGCTGGGAGGGG	14	21	0.0344
CAGGTAGATATTTGATAGGCAT	17	22	0.0344
TGGGGTAAGGATAGGAGGGTCA	14	23	0.0344
CATTACAGCACAGCCATTCT	16	21	0.203
TCCATGTTTCCTTCCCCCTTCT	20	26	0.203
CCTGCAGCGACTTGATGGCTTCC	15	26	0.35
AGGTGTGTCTGTAGAGTCC	14	21	0.332
TACAGATGCAGATTCTCTGACTTC	16	23	0.332
ACACAGTGCTTCATCCACTACT	18	24	0.332
ATCGCTGCGGTTGCGAGCGCTGT	19	27	0.332
ACAGGGCCGCAGATGGAGACT	15	19	0.0778
GTTAGGGCCAACATCTCTTGG	12	20	0.0778
CTCCAGAGGGATGCACTTTCT	16	21	0.0778
TGTCGTGGGGCTTGCTGGCTTG	16	22	0.0778
TATGTAGTAGTCAAAGGCATTT	17	22	0.0778
GTGGGTACGGCCCAGTGGGGGG	18	24	0.0344
GTGAGGAGGGGCTGGCAGGGAC	14	24	0.0344
GGGACCCAGGGAGAGACGTAAG	15	21	0.0344
CAGGATGTGGTCAAGTGTTGTT	13	22	0.0344
CAGGGCCAGGCACAGAGTAAG	17	22	0.0344
GCGAGGACCCCTCGGGGTCTGAC	14	23	0.0344
GGTGGGCTTCCCGGAGGG	13	22	0.0344
AGTGGGGTGGGACCCAGCTGTT	12	22	0.0344
TGGGCGAGGGGTGGGCTCTCAGAG	19	24	0.0344

CGGGTGGGAGCAGATCTTATTGAG	19	24	0.0344
ACTCTAGCTGCCAAAGGCGCT	14	22	0.0344
GCTTGTCGCTGCGGTGTTGCT	17	18	0.203
AATGGCTGTCCGTAGTATGGTC	15	21	0.203
TTGCCAGGGCAGGAGGTGGAA	14	23	0.203
GTGCGGAACGCTGGCCGGGGCG	13	23	0.203
TGTGTGGATCCTGGAGGAGGCA	17	24	0.203
CGTCCCGGGGCTGCGCGAGGCA	14	24	0.203
TGGGCTAAGGGAGATGATTGGGTA	18	24	0.203
GTGGGCTGGGCTGGGCTGGGCC	18	25	0.203
TCGACCGGACCTCGACCGGCT	13	22	0.203
TCTTCACCTGCCTCTGCCTGCA	14	24	0.203
GTTGGGGTGCAGGGGTCTGCT	13	19	0.0648
GAGGTTTGGGGAGGATTTGCT	14	23	0.0648
AGCTCTGCTGCTCACTGGCAGT	13	23	0.0344
GGTGGGGGGTGTTGTTTT	15	19	0.0344
AGGGAGGGACGGGGGCTGTGC	13	21	0.0344
AGCTGTCTGAAAATGTCTT	15	17	0.275
CAGGGGCTGGGGTTTCAGGTTCT	19	21	0.275
ACAGGCGGCTGTAGCAATGGGGG	13	22	0.275
GCAGGAACTTGTGAGTCTCCT	16	22	0.275
TCCAGTACCACGTGTCAGGGCCA	13	22	0.203
GTCCAGTTTTCCCAGGAATCCCT	16	23	0.203
CTCCGTTTGCCTGTTTCGCTG	16	23	0.203
TTTCCGGCTCGCGTGGGTGTGT	17	24	0.203
TGGGTGGAAGAAGGTCTGGTT	14	21	0.0344
TGATATGTTTGATATTGGGTT	17	22	0.0344
AGGCAGTGTAGTTAGCTGATTGC	20	22	0.0344
AGGCAGTGTATTGTTAGCTGGC	17	22	0.0344
GAGCAGGCGAGGCTGGGCTGAA	15	22	0.0344
AGGCGGGGCGCCGCGGGACCGC	14	22	0.0344
TAGGTAGTTTCCTGTTGTTGGG	16	23	0.0344
CAGGCAGGTGTAGGGTGGAGC	15	23	0.0344
TCCCCTTCTGCAGGCCTGCTGG	14	22	0.0344
CTGGGCTCGGGACGCGCGGCT	13	20	0.0778
CCTGAGCCCGGGCCGCGCAG	13	21	0.0778
GTTTGCACGGGTGGCCTTGTCT	12	22	0.0778
CATCTTACTGGGCAGCATTGGA	16	23	0.0778
CGTCTTACCCAGCAGTGTTTGG	17	23	0.0778
GAGTGTAGTTCTGAGCAGAGC	15	19	0.332
CGGGGTGGGTGAGGTCGGGC	16	21	0.332
AGCAGTGTTTGTTTTGCCCACA	15	21	0.332
ACAAGGTGTGCATGCCTGACC	17	22	0.332
ACAGGCGGCTGTAGCAATGGGGG	17	24	0.332
CTGGGGGGAGGAGACCCTGCT	14	20	0.143
ACTGGGGAGCAGAAGGAGAACC	15	21	0.143
GTTGGGGTGCAGGGGTCTGCT	17	21	0.143

CATTATTACTTTTGGTACGCG	17	24	0.111
TGTGTTAGAATAGGGGCAATAA	13	21	0.0778
TGGCTGCTTCCCTTGGTCTCCAG	13	22	0.0778
GAGGGTTGGGTGGAGGCTCTCC	16	23	0.0778
CAGGTCGTCTTGCAGGGCTTCT	16	23	0.0778
AGGGTGGGGCTGGAGGTGGGGCT	15	23	0.0778
TAAGGTGCATCTAGTGCAGTTAG	16	24	0.0778
GGGGGGGCAGGAGGGGCTCAGGG	12	24	0.0778
GTAGGGGCGTCCCGGGCGCGCGGG	20	25	0.0778
AATCTGAGAAGGCGCACAAGGT	13	20	0.0778
GCAGTTCTGAGCACAGTACAC	16	22	0.0778
TGGTCTGTCTCTGCCCTGGCAC	16	26	0.0778
ACCCTATCAATATTGTCTCTGC	15	22	0.0344
AGGGCCGAAGGGTGGAAGCTGC	17	25	0.0344
AGTATTCTGTACCAGGGAAGGT	16	21	0.0778
TGCCTCTTTTCCACGGCCTCAG	15	21	0.0778
TGCAACTTACCTGAGTCATTGA	17	21	0.0778
CTCATCTGCAAAGAAGTAAGTG	14	22	0.0778
ATTGCCTCTGTTCTAACACAAG	17	23	0.0778
CACTGCATTCCTGCTTGGCCCAG	18	24	0.0778
TATTGCACTTGTCCCGGCCTGT	21	24	0.0778
TCACCTGGCTGGCCCGCCCAG	19	27	0.0778
GCTAGTCCTGACTCAGCCAGT	15	20	0.203
TCTGGGGTCTTGGGCCATC	15	20	0.203
TCGGGCGCAAGAGCACTGCAGT	17	20	0.0344
TCGGGCCTGGGGTTGGGGGAGC	15	20	0.0344
GTAAGCAGGGGCTCTGGGTGA	16	20	0.0344
TTCACAGTGGCTAAGTTCTGC	17	21	0.0344
ACTGGACTTGGAGTCAGAAGGC	16	17	0.0344
ACTGGACTTGGAGTCAGGA	14	17	0.0344
ACTGGACTTAGGGTCAGAAGGC	16	17	0.0344
TCTGGTCCTGGACAGGAGGC	14	17	0.0344
TCTCTGGGCCTGTGTCTTAGGC	15	19	0.0344
CAAGTCTTATTTGAGCACCTGTT	12	23	0.0344
AGGGACTTTTGGGGGCAGATGTG	15	23	0.0344
AAAGACCCATTGAGGAGAAGGT	16	23	0.0344
GCGGTGATCCCGATGGTGTGAGC	18	24	0.0344
TGCCTGGAACATAGTAGGGACT	12	21	0.203
TGTCGTGGGGCTTGCTGGCTTG	17	21	0.203
GTGTCTGGGCGGACAGCTGC	14	22	0.203
ATGTATGTGTGCATGTGCATG	18	22	0.203
CAGGGGCTGGGGTTTCAGGTTCT	17	21	0.203
TGGCAGTGTCTTAGCTGGTTGT	16	24	0.203
GGGGCTGGGCGCGCGCC	12	18	0.143
AGAGGCTGAGAAGGTGATGTTG	17	19	0.143
TGAGGGGCAGAGAGCGAGACTTT	16	21	0.143
TGGGAGCTGGACTACTTC	16	21	0.143

TGGGAGGGCGTGGATGATGGTG	16	21	0.143
CTGGGAGGTGGATGTTTACTTC	18	22	0.143
GGCAGGAGGGCTGTGCCAGGTTG	14	21	0.111
AGCCAGGCTCTGAAGGGAAAGT	18	25	0.0344
CGGCGCGACCGGCCCGGGG	12	19	0.0344
AGGGACGGGACGCGGTGCAGTG	13	20	0.0344
TCTTCTCTGTTTTGGCCATGTG	17	21	0.203
GGCTTTCTAGTCTCAGCTCTCC	15	22	0.203
CACCTTTGTGTCCCCATCCTGCA	14	23	0.203
TGAGGACAGGGCAAATTCACGA	15	21	0.0344
CCCGGAGCCAGGATGCAGCTC	16	21	0.0778
GCGGGGGTGGCGGCGGCATCCC	15	21	0.0778
CTCGGGGCAGGCGGCTGGGAGCG	14	21	0.0778
TCCAGGGAGACAGTGTGTGAG	15	21	0.0778
CTCCTGAGCCATTCTGAGCCTC	15	22	0.0778
CTCTAGAGGGAAGCACTTTCTG	16	22	0.0778
CTCTAGAGGGAAGCGCTTTCTG	16	22	0.0778
CTCTAGAGGGAAGCACTTTCTC	15	22	0.0778
CTCTAGAGGGAAGCGCTTTCTG	16	22	0.0778
CTCTAGAGGGAAGCGCTTTCTG	16	22	0.0778
CTCTAGAGGGAAGCGCTTTCTG	16	22	0.0778
CTCTAGAGGGAAGCACTTTCTG	16	22	0.0778
CTCTAGAGGGAAGCGCTTTCTG	16	22	0.0778
CTCTAGAGGGAAGCGCTTTCTG	16	22	0.0778
CTCTAGAGGGAAGCACTTTCTG	16	22	0.0778
GGTGGGGCAATGGGATCAGGT	16	23	0.0344
TGGGAGAGAGGACTGTGAGGC	18	25	0.0344
TGGGGAGCTGAGGCTCTGGGGGTG	15	25	0.0344
AGCTGTCTGAAAATGTCTT	15	20	0.203
TAGCTTATCAGACTGATGTTGA	17	21	0.203
TTGCAGCTGCCTGGGAGTGACTTC	17	24	0.203
TCATTTATCTGTTGGGAAGCTA	17	23	0.111
GCAGTCCATGGGCATATACAC	16	19	0.0778
TGGTTCTAGACTTGCCAACTA	14	23	0.0778
AGGGGTGTGGAAAGAGGCAGAACA	14	25	0.0778
CCTGCGTGTTTTCTGTCCAA	15	20	0.35
TGGATGACAGTGGAGGCCT	14	17	0.0344
CTTGGGAGCCCTGTTAGACTC	15	19	0.0344
TCTGGCTCCGTGTCTTCACTCCC	13	24	0.0344
CCGCTTTCTGAGCTGGAC	14	18	0.203
ACTCCAGTTTTAGTTCTCTTG	16	22	0.203
CAGTGCCTCGGCAGTGCAGCCC	12	19	0.203
GCTGATGATGATGGTGCTGAAG	13	21	0.203
ATCTAAATGCAGCATGCCAGTC	15	22	0.203
TTAGCTTAAGGAGTACCAGATC	15	22	0.0344
AGGCAGGGGCTGGTGCTGGGCGGG	12	23	0.111
TGGCAGTGTCTTAGCTGGTTGT	16	23	0.111

ACTGGACTTGGAGTCAGAAGAGTGG	16	23	0.0344
GCTCTGACGAGGTTGCACTACT	16	25	0.0344
AGTTTGGGATGGAGAGAGGAGA	15	23	0.0104
TGAGGCCCTTGGGGCACAGTGG	16	20	0.203
TGTCTACTACTGGAGACACTGG	14	24	0.203
TTGCTAAGTAGGCTGAGATTGA	13	21	0.203
TGTGTCCGGGAAGTGGAGGAGG	14	22	0.203
GTGAGCCAGTGGAATGGAGAGG	17	23	0.203
CGGCTCTGGGTCTGTGGGGA	13	20	0.0344
TCTGGTATGTAGTAGGTAATAA	16	22	0.0344
CTCTAGCCACAGATGCAGTGAT	16	23	0.0344
TACTCTGGAGAGTGACAATCATG	13	25	0.0344
GCTGGGCGAGGCTGGCA	13	15	0.143
GAGGCTGATGTGAGTAGACCACT	13	23	0.0648
TGAGGCTCTGTTAGCCTTGGCTC	14	24	0.0648
CCGTCGCCGCCACCCGAGCCG	13	21	0.332
TTACACAGCTGGACAGAGGCA	14	21	0.332
ACACATGGGTGGCTGTGGCCT	16	21	0.332
TTGGACGGTAAGGTTAAGCAA	16	21	0.332
TCCAGTACCACGTGTCAGGGCCA	16	22	0.332
GTGCCAGCTGCAGTGGGGGAG	15	20	0.203
TGTGGGTTCTGGGTTGGGGTGA	15	23	0.203
CTGTGGGCTCAGCTCTGGG	15	20	0.203
CCTGTGCTCCCAGGGCCTCGC	13	21	0.203
TGTTTCGGGGCTCATGGCCTGTG	17	23	0.203
GTGCGTGGTGGCTCGAGGCGGGG	17	24	0.203
TCTGCAGGGTTTGCTTTGAG	15	17	0.143
AGGCTGCGGAATTCAGGAC	14	19	0.143
GAGGTTTGGGGAGGATTTGCT	16	20	0.143
TGGGGCGGAGCTTCCGGAG	13	21	0.143
GGGAGGTGTGATCTCACACTCG	14	22	0.143
AGGGGGTAGAAAGTGGCTGAAG	15	22	0.143
TGGGAGGAGGGGATCTTGGG	19	23	0.143
GGGGGGGCAGGAGGGGCTCAGGG	18	23	0.143
TGGGAGAGCAGGGTATTGTGGA	16	23	0.143
TGGGGGTGTGGGGAGAGAGAG	16	23	0.143
TTGGGATGGTAGGACCAGAGGGG	18	24	0.143
TTGGGGTGGTCGGCCCTGGAG	17	24	0.143
TCTGGGAGGTTGTAGCAGTGGAA	16	25	0.143
GGATCCGAGTCACGGCACCA	13	18	0.0344
GTAAGGGACCGGAGAGTAGGA	14	21	0.0344
GGGCTAGGGCCTGCTGCCCCC	14	23	0.0344
TCTGCCATAGGAAGCTTGGAGTGG	17	21	0.0344
AATTGCACTTTAGCAATGGTGA	18	22	0.0344
CCAGAGGTGGGGACTGAG	14	20	0.0778
AGTCATTGGAGGGTTTGAGCAG	16	23	0.111
CACTGGCTCCTTTCTGGGTAGA	16	22	0.0344

GAGCTTGGTCTGTAGCGGTT	13	19	0.0344
AGGCTGGGCTGGGACGGA	13	19	0.0344
ACTCGGCGTGGCGTCGGTCGTG	15	23	0.0344
ACTCGGCTGCGGTGGACAAGT	18	23	0.0344
TGCGGGGCTAGGGCTAACAGCA	14	20	0.0344
CGGGTGGATCACGATGCAATTT	12	22	0.0344
TGGGGGAGGAAGGACAGGCCAT	13	22	0.0344
AGTGCCTGCTATGTGCCAGGCA	15	21	0.0344
TCCACTCTCCTGGCCCCCAG	13	20	0.203
TTCCGCCAGTCGGTGGCCGG	15	21	0.203
CTCCCCGGCCTCTGCCCCCAG	15	21	0.203
ACTTCACCTGGTCCACTAGCCGT	16	22	0.203
CCTTCACTGTGACTCTGCTGCAG	17	22	0.203
GGATATCATCATATACTGTAAG	16	24	0.203
TGACCCACCCCTCTCCACCAG	14	24	0.203
TGGCTTCTCTTGCACACCCAG	16	25	0.203
GGGCTAGGGCCTGCTGCCCCC	14	24	0.143
TCACACCTGCCTCGCCCCCC	16	21	0.0778
TCACCCTGCATCCCGCACCCAG	19	21	0.0778
CCGCTCTTCCCCTGACCCCAG	15	21	0.0778
ATTGGACTGCTGATGGCCCGT	15	22	0.0778
TCCACTCTCCTGGCCCCCAG	14	22	0.0778
TGCCTACTGAGCTGAAACACAG	15	23	0.0778
TGCCGCCCTCTCGCTGCTCTAG	17	23	0.0778
CGGCAACAAGAAACTGCCTGAG	14	24	0.0778
GGGACCATCCTGCCTGCTGTGG	18	24	0.0778
AGGGCTGGACTCAGCGGCGGAGCT	15	25	0.0778
CATAGCCCGGTCGCTGGTACATGA	15	26	0.0778
AGGGGTGGTGTTGGGACAGCTCCGT	17	26	0.0778
CTCATTTAAGTAGTCTGATGCC	17	22	0.0344
TGGTCTAGGATTGTTGGAGGAG	13	21	0.0344
TCTGCACTGTGAGTTGGCTGGCT	16	25	0.0344
AGGCGGAGACTTGGGCAATTG	14	20	0.0344
AGGCAAGATGCTGGCATAGCT	16	20	0.0344
TGAGGTAGGAGGTTGTATAGTT	15	22	0.0344
CAGGATCCACAGAGCTAGTCCA	12	22	0.143
TTGGGATCATTTTGCATCCATA	14	24	0.143
TGAGGGACAGATGCCAGAAGCA	16	22	0.0344
GTGAGTGGGAGCCCCAGTGTGTG	12	23	0.0344
AGCGAGGTTGCCCTTTGTATAT	13	24	0.0344
TGGGTAGAGAAGGAGCTCAGAGGA	18	26	0.0344
TGTTTGTTGTAAGGATCGTTGT	18	19	0.0344
GTGAGACTTCTCTCCCTTCAG	14	20	0.0344
TGGTGGGCACAGAATCTGGACT	13	22	0.0344
TGGTGCGGAGAGGGCCCACAGTG	13	22	0.0344
TCTTCACCTGCCTCTGCCTGCA	16	20	0.275
TGTGCTTGCTCGTCCCGCCCGCA	16	22	0.275

TGTTGTCATGTTTTTTCCCTAG	17	22	0.275
AGAGGCTGAGAAGGTGATGTTG	13	23	0.0648
CACTGTTTCACCACTGGCTCTT	15	23	0.203
AGGCATGGGAGGTCAGGTGA	14	21	0.0344
AGGCATGGGAGGTCAGGTGA	14	21	0.0344
TGGGGGAGTGCAGTGATTGTGG	16	20	0.0344
GGGGCTGGGGCCGGGGCCGAGC	15	20	0.0344
GTGGGTGCTGGTGGGAGCCGTG	13	21	0.0344
CTGGGAGGGGCTGGGTTTGGC	13	21	0.0344
TTGGGGGTGAGGTTGGTGTCTGG	14	23	0.0866
GAGGCTGAAGGAAGATGG	12	18	0.0648
AGAGGCTGGCCGTGATGAATTC	14	19	0.0648
TGAGGTGGTAGGATGTAGA	12	19	0.0648
TTGTGGCTGGTCATGAGGCTAA	13	20	0.0648
GGGTTTGTAGCTTTGCTGGCATG	14	24	0.0648
TGAGCTAAATGTGTGCTGGGA	14	21	0.0344
GCGGGGGTGGCGGCGGCATCCC	15	25	0.0344
CTTGAGTCGTGCCTTTCTGAATG	15	22	0.35
GGGTGCGGGCCGGCGGGG	12	17	0.332
AGGTGGTCCGTGGCGCGTTCGC	17	21	0.332
CACTCAGCCTTGAGGGCACTTTC	16	26	0.332
GGATGGTTGGGGGCGGTCGGCGT	13	24	0.203
TCTGCCCCCTCCGCTGCTGCCA	12	22	0.0778
TTTGTGACCTGGTCCACTAACC	13	22	0.0778
AGCGCGGGCTGAGCGCTGCCAGTC	16	24	0.332
TAGGCAGTGTCATTAGCTGATTG	17	24	0.332
CACATATGAAGTGAGCCAGCAC	18	24	0.332
CCTGCAGAGAGGAAGCCCTTC	18	25	0.332
CCCGGACAGGCGTTCGTGCGACGT	17	26	0.332
TGCGGGGACAGGCCAGGGCATC	15	19	0.143
CATGGGGTAGGGCAGAGTAGG	13	19	0.143
ACACATGGGTGGCTGTGGCCT	14	20	0.143
CAGGGTCAGCTGAGCATG	15	17	0.0778
CCAGTGTGGCTCAGCGAG	12	18	0.0778
TCTAGGCTGGTACTGCTGA	13	19	0.0778
CCTATTCTTGGTTACTTGCACG	17	23	0.0778
CCTGTCTGTGCCTGCTGTACA	17	23	0.0778
AGTTGCCAGGGCTGCCTTTGGT	14	24	0.0778
TGCACCATGGTTGTCTGAGCATG	16	25	0.111
TGGGCTGGCAGGGCAAGTGCTG	15	18	0.0344
CCAGTGACTGAGCTGGAGCCA	14	22	0.0344
GGGTGCGGGCCGGCGGGG	12	18	0.0344
TGGGCTGAGGGCAGGAGGCCTGT	17	19	0.0344
TGGGGGAGATGGGGGTTGA	13	19	0.0344
GTGGGTTGGGGCGGGCTCTG	17	20	0.0344
CTGGGTTGGGCTGGGCTGGG	14	20	0.0344
GGGCTAGGGCCTGCTGCCCCC	13	24	0.0344

CCTGCAGCGACTTGATGGCTTCC	14	27	0.0344
GTGGGTTGGGGCGGGCTCTG	14	20	0.203
CTGGGTTGGGCTGGGCTGGG	14	20	0.203
CATGCCTTGAGTGTAGGACCGT	14	21	0.203
CAGTGGCCAGAGCCCTGCAGTG	12	22	0.203
GTGTCTGCTTCCTGTGGGA	12	19	0.203
CTTTCAGTCGGATGTTTGCAGC	18	23	0.203
CTTTCAGTCAGATGTTTGCTGC	19	23	0.203
CTTTCAGTCGGATGTTTACAGC	17	23	0.203
CAGGGCTGGCAGTGACATGGGT	17	20	0.0778
GGGAGCCAGGAAGTATTGATGT	16	20	0.0778
TAGGGTAGACACTGACAACGTT	14	20	0.0778
CGGGGCCAGAGCAGAGAGC	14	20	0.0778
TGGGGCGGGGCAGGTCCCTGC	14	20	0.0778
TTAGGGCCCTGGCTCCATCTCC	16	21	0.0778
TCAGCAGGCAGGCTGGTGCAGC	12	21	0.0778
TTAGGCCATCATCCCATTATGC	18	21	0.0778
TTTAGTGTGATAATGGCGTTTGA	17	22	0.0778
ACCAGGGCCAGCAGGGAATGT	15	22	0.0778
TTGGGGGTGAGGTTGGTGTCTGG	16	22	0.0778
CTCCTGGGGCCCGCACTCTCGC	19	23	0.0778
CTGTACAGGCCACTGCCTTGC	19	25	0.0778
AGCTACATTGTCTGCTGGGTTTC	15	21	0.0344
AGGTTGCCTGTGAGGTGTTCA	14	22	0.0344
TAAGGTGCATCTAGTGCAGTTAG	16	23	0.0344
CAAGCTCGTGTCTGTGGGTCCG	14	23	0.0344
TATCCAGCTTGTTACTATATGC	15	18	0.0778
TGCTAGTCTGGACTGATATGGT	16	21	0.0778
GTGCATTGCTGTTGCATTGC	15	22	0.0778
GTGGTTATCCCTGCTGTGTTCG	15	22	0.0778
AGGTGTTATCCTATCCATTTGC	19	24	0.0778
TGAGGGCTCCAGGTGACGGTGG	19	26	0.0778
TGGGGGCTGGGATGGGCCATGGT	18	26	0.0778
GTGGGTTGGGGCGGGCTCTG	14	21	0.0344
AGCTACATCTGGCTACTGGGT	17	24	0.0344
AGTGGCACATGTTTGTTGTGAG	18	24	0.0344
GGGCTGGGGCGCGGGGAGGT	15	25	0.0344
CTTTCAGTCGGATGTTTGCAGC	16	22	0.203
CTTTCAGTCAGATGTTTGCTGC	17	22	0.203
CTTTCAGTCGGATGTTTACAGC	16	22	0.203
TAGTGCAATATTGCTTATAGGGT	13	21	0.203
TTAATGCTAATCGTGATAGGGGT	14	22	0.203
TCAAATGCTCAGACTCCTGTGGT	15	23	0.203
CCTGAGCCCGGGCCGCGCAG	12	23	0.203
GGTCTAGGCCCGGTGAGAGACTC	13	25	0.203
GCCGGGGCTTTGGGTGAGGG	17	20	0.203
TGCTAGTCTGGACTGATATGGT	15	21	0.203

GTGCGTGGTGGCTCGAGGCGGGG	18	22	0.203
CAGGGCCCTGGCTTTAGCAGA	15	19	0.0344
AGCGGTGCTCCTGCGGGCCGA	13	21	0.0344
GGCTGGGTGCTCTTGTGCAGT	17	21	0.0344
AGGCAGGGGCTGGTGCTGGGCGGG	17	22	0.0344
CAGGCAGTGACTGTTCAGACGTC	15	23	0.0344
AGCAGTGTTTGTTTTGCCCACA	14	22	0.0344
GGTAGTGAGTTATCAGCTAC	14	19	0.0648
GAGGTTTTCTGGGTTTCTGTTTC	14	24	0.0648
AGGGCTGGACTCAGCGGCGGAGCT	13	24	0.0648
AGGGGTGTGGAAAGAGGCAGAACA	15	24	0.0648
TGGCGGGGGTAGAGCTGGCTGC	14	20	0.143
GGGGCTGGGGCCGGGGCCGAGC	14	21	0.143
TGAGGCGGGGGGGCGAGC	16	22	0.143
TGGGGCTAGTGATGCAGGACG	15	22	0.143
AGAGGCTGGCCGTGATGAATTC	12	22	0.143
GGGATGGTAGACCGGTGACGTGC	16	23	0.143
GGGGGGTGTGGAGCCAGGGGGC	16	23	0.143
TGGGTGGTCTGGAGATTTGTGC	17	24	0.143
CGGGAGCTGGGGTCTGCAGGT	16	24	0.143
CTGGGAGGTGTGATATTGTGGT	18	25	0.143
TCTGGGGATGAGGACAGTGTGT	18	26	0.143
ACTGGGGGCTTTCGGGCTCTGCGT	19	26	0.143
AGGCTGCGGAATTCAGGAC	13	16	0.0778
TGAGCTGCTGTACCAAAAT	13	18	0.0778
GTAGGGGAGGTTGGGCCAGGGA	16	19	0.0778
ATCATGATGGGCTCCTCGGTGT	17	22	0.0778
TTCACCACCTTCTCCACCCAGC	16	21	0.203
ACTCCATTTGTTTTGATGATGGA	15	23	0.203
GTTTCACCATGTTGGTCAGGC	16	23	0.203
ACTCCAGCCCCACAGCCTCAGC	17	23	0.203
AGACTTCCCATTTGAAGGTGGC	18	25	0.203
TACTGCAGACGTGGCAATCATG	13	19	0.0778
TCTACAGTGCACGTGTCTCCAGT	17	23	0.0778
GCCTGCTGGGGTGGAACCTGGT	15	23	0.0778
GCTGAACTGGGCTGAGCTGGGC	18	23	0.0778
AGCAGCATTGTACAGGGCTATGA	16	24	0.0778
AGCAGCATTGTACAGGGCTATCA	16	24	0.0778
TGCCTACTGAGCTGATATCAGT	16	24	0.0778
CGCCGCGCGCATCGGCTCAGC	19	24	0.0778
GGCTGGGTGCTCTTGTGCAGT	16	24	0.0778
TAGGCACACTTAAAGTTATAGC	14	25	0.0778
GATCCATCTCTGCCTGTATTGGC	18	25	0.0778
TGGGCTGAGGGCAGGAGGCCTGT	16	26	0.0778
GGGGCTGGGGCCGGGGCCGAGC	15	26	0.0778
CAGGCAGTGACTGTTCAGACGTC	14	24	0.0344
CAGGTCGTCTTGCAGGGCTTCT	18	24	0.0344

TGGGTGTAGGCTGGAGCTGAGG	16	24	0.0344
TTTCAAGCCAGGGGGCGTTTTTC	15	23	0.0344
AGGGCTTAGCTGCTTGTGAGCA	16	23	0.0344
GGGTGAGGGCAGGTGGTT	12	17	0.0344
CTCTAGAGGGAAGCACTTTCTG	12	26	0.0344
CTCTAGAGGGAAGCACTTTCTC	12	26	0.0344
CTCTAGAGGGAAGCACTTTCTG	12	26	0.0344
CTCTAGAGGGAAGCACTTTCTG	12	26	0.0344
GTGGGCTGGGCTGGGCTGGGCC	14	23	0.0344
AGAGGCTGAGAAGGTGATGTTG	16	24	0.0344
ACAGTAGTCTGCACATTGGTTA	16	23	0.0344
ACAGTAGTCTGCACATTGGTTA	16	23	0.0344
TGTCGTGGGGCTTGCTGGCTTG	18	24	0.0344
AGCGGTGCTCCTGCGGGCCGA	14	20	0.0344
TGTCTCTCGCCCTTGGCCTTAG	15	23	0.275
TCTGTATTCTCCTTTGCCTGCAG	15	25	0.275
GGCTTTCTAGTCTCAGCTCTCC	18	21	0.203
TATTGCACTCGTCCCGGCCTCC	16	21	0.203
TGTGACTGCATTATGAAAATTCT	18	23	0.203
GTGAGTCAGGGTGGGGCTGG	15	21	0.332
TCACAGTGGTCTCTGGGATTAT	14	26	0.332
TATACAAGGGCAAGCTCTCTGT	18	27	0.332
CAGTTATCACAGTGCTGATGCT	17	19	0.203
ATCCAGTTCTCTGAGGGGGCT	14	22	0.203
GGCTGGAGCGAGTGCAGTGGTG	13	23	0.0648
GGCTGAGTGGGGTTCTGACTCC	14	23	0.0648
AGGCTCTAACTGGCTTTCCCTGCA	13	24	0.0648
GGAGGCCGGGGTGGGGCGGGGCGG	15	26	0.0648
GGGTGAGGGCAGGTGGTT	14	20	0.143
GGGCTGGGGCGCGGGGAGGT	15	21	0.143
TGGGCTCAGGGTACAAAGGTT	15	22	0.143
AGGGCTTAGCTGCTTGTGAGCA	13	22	0.143
AGGGCTGGACTCAGCGGCGGAGCT	13	22	0.143
TGAGGTAGTAGGTTGTGTGGTT	14	23	0.143
TGAGGTAGTAGGTTGTATGGTT	14	23	0.143
AGAGGTAGTAGGTTGCATAGTT	13	23	0.143
AGGGGCTGGCTTTCCTCTGGTC	15	23	0.143
TGGGGCGGAGCTTCCGGAGGCC	14	23	0.143
GGAGGCGCAGGCTCGGAAAGGCG	15	23	0.143
AGGGGGCGCAGTCACTGACGTG	16	24	0.143
TGGGAGGGGAGAGGCAGCAAGCA	16	25	0.143
TGCGGGGCTAGGGCTAACAGCA	16	25	0.143
TGGGAGAGAGGACTGTGAGGC	16	25	0.143
TTGAGGAGACATGGTGGGGGCC	15	20	0.0778
CTCGTGGGCTCTGGCCACGGCC	17	23	0.0778
CTCGGGCGGAGGTGGTTGAGTG	15	21	0.332
CCCGCAGGTGAGATGAGGGCT	16	21	0.332

AGGCTGTGATGCTCTCCTGAGCCC	17	22	0.143
TGAGGCTCTGTTAGCCTTGGCTC	14	24	0.143
GGAGGCCGGGGTGGGGCGGGGCGG	14	24	0.143
GGATGGTTGGGGGCGGTCGGCGT	18	24	0.143
GCTGACTCCTAGTCCAGGGCTC	15	21	0.275
AGGCTGTGATGCTCTCCTGAGCCC	14	23	0.275
GGTAGTGAGTTATCAGCTAC	16	20	0.0344
TCAGGCCAGGCACAGTGGCTCA	15	23	0.0344
TCAGGCAGTGTGGGTATCAGAT	14	23	0.0344
CTCGGCGCGGGGCGCGGGCTCC	17	24	0.0344
CCTGCGTGTTTTCTGTCCAA	14	21	0.0344
GGCTGGAGCGAGTGCAGTGGTG	16	22	0.0344
AGGCTGTGATGCTCTCCTGAGCCC	12	23	0.0344
ACTCGCATCCTTCCCTTGGCAG	13	20	0.0344
TGAGGCTCTGTTAGCCTTGGCTC	15	25	0.0344
TCATATTGCTTCTTTCT	15	19	0.0778
ATCACATTGCCAGGGATTTCC	13	20	0.0778
ATCACATTGCCAGTGATTACCC	16	20	0.0778
TGCGACATTGGAAGTAGTATCA	14	21	0.0778
GGCCACTGAGTCAGCACCA	14	21	0.0778
AGCTACAGTTACTTTTGCACCA	16	21	0.0778
GGCTAGCAACAGCGCTTACCT	17	21	0.0778
AGACACATTTGGAGAGGGACCC	15	21	0.0778
AGCACACTGAGCGAGCGGAC	14	21	0.0778
TGGCGGCGGTAGTTATGGGCTT	15	22	0.0778
CGAGCCTCAAGCAAGGGACTT	15	23	0.0778
AGAGCTTAGCTGATTGGTGAAC	17	23	0.0778
AGAGTTAACTCAAAATGGACTA	17	23	0.0778
TGAGCCGAGCTGAGCTTAGCTG	15	23	0.0778
TGGGCCAGGGAGCAGCTGGTGGG	13	23	0.0778
AGGGTGGGGCTGGAGGTGGGGCT	16	23	0.0778
CAGTGCCTCGGCAGTGCAGCCC	17	24	0.0778
AGGAGCTATCCACTCCAGGTGTCC	17	24	0.0778
AAGGGGCAGGGACGGGTGGCCC	15	24	0.0778
CAGGTCGTCTTGCAGGGCTTCT	17	24	0.0778
TTAGGCCGCAGATCTGGGTGA	17	25	0.0778
CCAGGGAGGCTGGTTTGGAGGA	16	25	0.0778
GCAGGTGCTCACTTGTCCTCCT	18	25	0.0778
CTATGGCGAGACTGGCATGTACTC	20	25	0.0778
GGTCTAGGCCCGGTGAGAGACTC	18	28	0.0778
TAACAGTCTCCAGTCACGGCC	15	22	0.275
TGATTGTCTTCATATCTAGAAC	15	22	0.275
CGGGCTGTCCGGAGGGGTCGGCT	14	23	0.275
TCGTGGCCTGGTCTCCATTAT	12	20	0.332
TCAGGCCTCTTTCTACCTT	12	20	0.332
CATCAGCACCCTATGTCCTTTCT	14	22	0.0778
TGCATCAGGCCAGAAGACATGAG	14	24	0.0778

TTGCTCTGCTCCCCCGCCCCCAG	16	25	0.0778
GGAGGCCGGGGTGGGGCGGGGCGG	16	25	0.0344
CGGGGCCATGGAGCAGCCTGTGT	14	25	0.0344
CTCGGGCGGAGGTGGTTGAGTG	16	20	0.0344
CTTTCAGTCGGATGTTTGCAGC	16	22	0.0344
ATGTATGTGTGCATGTGCATG	13	21	0.0344
GGCTGGGTGCTCTTGTGCAGT	15	23	0.0344
TGAGTGTGTGTGTGTGAGTGTGT	18	25	0.0344
ATCACATTGCCAGGGATTACC	13	19	0.0778
AGTGGCACATGTTTGTTGTGAG	14	20	0.0778
CAGCCACAACTACCCTGCCACT	14	21	0.0778
AGGGCTTAGCTGCTTGTGAGCA	16	22	0.0778
TGAGACCAGGACTGGATGCACC	16	22	0.0778
TGGGCCATGCATTTCTAGAACT	15	22	0.0778
GAGGGCAGCGTGGGTGTGGCGGA	16	23	0.0778
GAGGGCATGCGCACTTTGTCC	19	23	0.0778
AGGAGCAGTGCCGGCCAAGGCGCC	14	23	0.0778
GGGGGTCCCCGGTGCTCGGATC	17	23	0.0778
CAGGGCCCTGGCTTTAGCAGA	15	23	0.0778
CCTGGGCAGCGTGTGGCTGAAGG	15	24	0.0778
CCAGAGCAGCCTGCGGTAACAGT	14	24	0.0778
CTTGAGTCGTGCCTTTCTGAATG	13	24	0.0778
CTCGGCGCGGGGCGCGGGCTCC	16	25	0.0778
TCTACAGTGCACGTGTCTCCAGT	17	25	0.0778
ATGTATGTGTGCATGTGCATG	12	20	0.0778
TTGTGTGAGTACAGAGAGCATC	16	20	0.0778
GCTGACAGCAGGGCTGGCCGCT	15	21	0.0778
GCTGCGGGCTGCGGTCAGGGCG	17	21	0.0778
TACTGCATCAGGAACTGATTGGA	13	22	0.0778
AGGTGTGTCTGTAGAGTCC	16	22	0.0778
AACTCTGTCTTCACTCATGAGT	14	22	0.0778
CACTGTTTCCTTTCTGAGTGGA	13	22	0.0778
TATTGCACTCGTCCCGGCCTCC	19	22	0.0778
CACTGTTTCACCACTGGCTCTT	13	22	0.0778
ACCTTGCCTTGCTGCCCGGGCC	19	23	0.0778
ATACTGTGAATTTCACTGTCACA	18	23	0.0778
ACTTGTATGCTAGCTCAGGTAG	16	23	0.0778
GGGGCTGTGATTGACCAGCAGG	16	24	0.0778
CGCGCCTGCAGGAACTGGTAGA	14	24	0.0778
CCAATATTGGCTGTGCTGCTCC	17	25	0.0778
AGCTACATTGTCTGCTGGGTTTC	21	27	0.0778
TGGGGCGGGGCAGGTCCCTGC	15	23	0.275
CAGCAGCAATTCATGTTTTGAA	16	24	0.275
GGGGAACTGTAGATGAAAAGGC	14	24	0.275
TAGGAGCTCAACAGATGCCTGTT	14	24	0.275
TCAGCTGGCCCTCATTTC	12	19	0.332
ACATCCTGCTCCACAGGGCAGAGG	14	22	0.0778

TGTCTCTCGCCCTTGGCCTTAG	14	23	0.0778
TTGGGCTGGGCTGGGTTGGG	17	22	0.0344
CGGTGAGCGCTCGCTGGC	14	18	0.332
ACGGTGCTGGATGTGGCCTTT	13	19	0.332
CCAGTGTGGCTCAGCGAG	15	19	0.332
CAGGCAGTGACTGTTCAGACGTC	16	22	0.332
CACACAGGAAAAGCGGGGCCCTG	14	22	0.332
AGCACACTGAGCGAGCGGAC	17	22	0.332
TCAGGCAGTGTGGGTATCAGAT	15	23	0.332
TTGCACTCTGGCCTTCTCCCAGG	15	22	0.0344
AGCTACATTGTCTGCTGGGTTTC	16	23	0.0344
CACTGCATTCCTGCTTGGCCCAG	16	23	0.0344
GTGTGGCCGGCAGGCGGGTGG	14	24	0.0344
CAGGATCCACAGAGCTAGTCCA	15	22	0.0344
GTGTGGCCGGCAGGCGGGTGG	16	23	0.0344
AACCCAGTGGGCTATGGAAATG	12	20	0.0778
TTGGGCCCAGGAGTAAACAGGAT	12	23	0.0778
TGAGGAGATGCTGGGACTGA	13	18	0.143
GGGGAGGTGTGCAGGGCTGG	13	18	0.143
TGGGGGAGATGGGGGTTGA	15	18	0.143
CTGGGACAGGAGGAGGAGGCAG	15	19	0.143
CTGGGAGGGGCTGGGTTTGGC	16	19	0.143
GCTGGGAAGGCAAAGGGACGT	16	20	0.143
GCAGGGACAGCAAAGGGGTGC	14	20	0.143
CCACCGGGGGATGAATGTCAC	16	22	0.143
AGCTACATCTGGCTACTGGGT	14	19	0.0778
AGGTTACCCGAGCAACTTTGCAT	17	20	0.0778
TGGCCGGATGGGACAGGAGGCAT	14	20	0.0778
GGGCCTCTCTTGTCATCCTGCAG	17	20	0.0778
AGAGCTGGCTGAAGGGCAG	14	21	0.0778
TGGGCTGGCAGGGCAAGTGCTG	14	21	0.0778
TGAGCTAAATGTGTGCTGGGA	15	21	0.0778
CAGGGCCTCACTGTATCGCCCA	16	22	0.0778
TAGCAGCGGGAACAGTTCTGCAG	14	22	0.0778
AGGAGGCAGCGCTCTCAGGAC	16	22	0.0778
CGGGGCCATGGAGCAGCCTGTGT	17	22	0.0778
ACGGATGTTTGAGCATGTGCTA	14	23	0.0778
TCTAGAGGAAGCACTTTCTGTTT	17	24	0.0778
CCCTGGGCCTCTGCTCCCCAG	13	24	0.0778
ATTGGGAACATTTTGCATGTAT	19	25	0.0778
TTCCTGGGCTTCTCCTCTGTAG	17	25	0.0778
GTGTCTGGGCGGACAGCTGC	15	17	0.0778
TTGCACTTGTCTCAGTGA	15	19	0.0778
GCTGCACCGGAGACTGGGTAA	15	20	0.0778
ACTCGCATCCTTCCCTTGGCAG	15	20	0.0778
CAGTGCAATGTTAAAAGGGCAT	14	21	0.0778
CACTGCAGGACTCAGCAG	16	21	0.0778

CCACTGCCTATGCCCCACAG	15	22	0.0778
TGGACTGCCCTGATCTGGAGA	13	23	0.0778
CACATATGAAGTGAGCCAGCAC	17	23	0.0778
CGCACCTGCCTCTCACCCACAG	19	23	0.0778
CCGCACTGTGGGTACTTGCTGC	20	24	0.0778
CCAGACTGTGGCTGACCAGAGG	15	24	0.0778
CCACGTGCTTCTCTTTCCGCAG	17	24	0.0778
CCCTGCTGCCTTCACCTGCCAG	15	24	0.0778
TTCCACTGCCACTACCTAATTT	14	25	0.0778
GGCTACAACACAGGACCCGGGC	19	26	0.0778
TGTCACTCGGCTCGGCCCACTAC	18	26	0.0778
CAGCCGCCGCCTGTCTCCACAG	18	27	0.0778
GGGAGTGCAGGGCAGGGTTTC	15	20	0.0778
TGGGGAGCGGCCCCCGGGTGGG	14	21	0.0778
AGGGGTGCTATCTGTGATTGA	12	21	0.0778
AAAGGTGCTCAAATTAGACAT	16	21	0.0778
GTGAGTGTGGATCCTGGAGGAAT	18	22	0.0778
CAGAAGGGGAGTTGGGAGCAGA	15	23	0.0778
CATGGGGTAGGGCAGAGTAGG	15	23	0.0778
TGGCTGTTGGAGGGGGCAGGC	15	21	0.0778
GGGGCTGGGGCCGGGGCCGAGC	18	21	0.0778
TAGGAGCTCAACAGATGCCTGTT	13	22	0.0778
AAGGTATTGTTCAGACTTATGA	18	22	0.0778
TGGGTGTAGGCTGGAGCTGAGG	12	22	0.0778
TAAGGTGCATCTAGTGCAGATAG	19	23	0.0778
TGGGGGTGGTCTCTAGCCAAGG	16	23	0.0778
TGGGGGCTGGATGGGGTAGAGT	15	23	0.0778
TAGGTTATCCGTGTTGCCTTCG	13	22	0.284
TAAGTGCTTCCATGTTTGAGTGT	16	22	0.284
AAAGTTCTGAGACACTCCGACT	14	21	0.0866
TAGAGTCTGGCTGATATGGTTT	14	23	0.111
ACAGTAGTCTGCACATTGGTTA	20	24	0.111
ACAGTAGTCTGCACATTGGTTA	20	24	0.111
GTGTGCGGAAATGCTTCTGCTA	12	21	0.203
GTGTGGCCGGCAGGCGGGTGG	16	23	0.203
TGCCTGGGTCTCTGGCCTGCGCGT	15	26	0.203
TGGGGCGGAGCTTCCGGAG	15	20	0.0344
TTGTGGCTGGTCATGAGGCTAA	15	21	0.0344
TGGGTGGTCTGGAGATTTGTGC	14	21	0.0344
TGGGTGTAGGCTGGAGCTGAGG	15	21	0.0344
CATCTGGCATCCGTCACACAGA	17	20	0.0778
TGCATCACAGCCTTTGGCCCTAG	15	22	0.0778
TTGCACTCTGGCCTTCTCCCAGG	16	23	0.0778
TCTGCCATCCTCCCTCCCCTAC	13	24	0.0778
CCTGCAACTTTGCCTGATCAGA	17	24	0.0778
TCTGTGCTTCACCCCTACCCAG	16	24	0.0778
ACTGGGTAGGTGGGGCTCCAGG	15	24	0.0778

TATTCATTTATCCCCAGCCTACA	18	25	0.0778
TCTGGGTGCAGTGGGGGTT	13	20	0.203
CTGGGAGAAGAGTGGTGAAGA	16	23	0.143
TTGGGGGTGAGGTTGGTGTCTGG	17	23	0.143
TCTGTGGAGTGGGGTGCCTGT	16	24	0.143
CAGTTGGGTCTAGGGGTCAGGA	17	25	0.143
CTGGGTGAGGGCATCTGTGGT	14	22	0.0344
GTGCGTGGTGGCTCGAGGCGGGG	14	23	0.0344
CGGTGAGCGCTCGCTGGC	15	19	0.0344
TGGCAGTGTATTGTTAGCTGGT	16	22	0.0344
TCAGCAGGCAGGCTGGTGCAGC	19	24	0.0344
CTTTCAGTCAGATGTTTGCTGC	17	19	0.0344
AGTATTCTGTACCAGGGAAGGT	14	22	0.0344
GTGCATTGCTGTTGCATTGC	16	21	0.111
ATCGCTGCGGTTGCGAGCGCTGT	18	25	0.111
TCTGTCATTTCTTTAGGCCAATA	17	21	0.35
CCTGCGAGTCTCCGGCGGTGG	16	21	0.35
AGCGGTGCTCCTGCGGGCCGA	14	19	0.332
CCGCACTGTGGGTACTTGCTGC	17	22	0.332
TGGGCTGAGGGCAGGAGGCCTGT	15	26	0.143
TGGCTGTTGGAGGGGGCAGGC	15	21	0.0344
CGGGACTGTAGAGGGCATGAGC	18	23	0.0344
CAGGCAGGTGTAGGGTGGAGC	12	20	0.0344
GGGAGGTGTGATCTCACACTCG	13	23	0.0344
GGGGGGGCAGGAGGGGCTCAGGG	17	24	0.0344
CTCTAGAGGGAAGCGCTTTCTG	15	25	0.0344
CTCTAGAGGGAAGCGCTTTCTG	15	25	0.0344
CTCTAGAGGGAAGCGCTTTCTG	15	25	0.0344
CTCTAGAGGGAAGCGCTTTCTG	15	25	0.0344
AGGCTGGAGTGAGCGGAG	13	18	0.0648
AGGTTGCCTGTGAGGTGTTCA	15	18	0.0648
TGAGGTAGTAGTTTGTGCTGTT	14	20	0.0648
TGAGTGTGTGTGTGTGAGTGTGT	16	20	0.0648
TGAGGGTGTCAGCAGGTGACG	16	20	0.0648
GGGGAGCTGTGGAAGCAGTA	14	20	0.0648
TTGGGCTGGGCTGGGTTGGG	14	21	0.0648
GTGGGTGCTGGTGGGAGCCGTG	15	21	0.0648
TTGGGGATTGGGTCAGGCCAGT	13	21	0.0648
TTTGAGGCTACAGTGAGATGTG	15	22	0.0648
CTCTAGAGGGAAGCGCTTTCTG	15	25	0.0344
CTCTAGAGGGAAGCGCTTTCTG	15	25	0.0344
TTGCTCTGAGCTCCGAGAAAGC	12	22	0.0778
TCTGTAGCCTGGGAGCAATGGGGT	12	23	0.0778
GGCGGGTGCGGGGGTGG	13	16	0.143
GGGGCGCGGCCGGATCG	12	16	0.143
ATAGTGGGAAGCTGGCAGATTC	16	21	0.143
AGAGGTATAGGGCATGGGAA	13	20	0.0778

AGAGCTCACAGCTGTCCTTCTCTA	16	20	0.0778
TAGCAGCACATAATGGTTTGTG	18	21	0.0778
AGGAGCTAGCCAGGCATATGCA	17	21	0.0778
TAGAGTCTGGCTGATATGGTTT	14	21	0.0778
CCGGGGCAGATTGGTGTAGGGTG	15	22	0.0778
TTTCAAGCCAGGGGGCGTTTTTC	16	24	0.0778
GTTGGGGTGCAGGGGTCTGCT	15	19	0.0344
GCGGGGCTGGGCGCGCG	13	16	0.0778
GTGCATTGTAGTTGCATTGCA	17	23	0.0778
AGGGCTGGACTCAGCGGCGGAGCT	16	25	0.0778
AGGAGCTAGCCAGGCATATGCA	17	25	0.0778
CGGGCTGTCCGGAGGGGTCGGCT	15	25	0.0778
TAGGGGCAGCAGAGGACCTGGG	18	27	0.0778
AGTTTGGGATGGAGAGAGGAGA	15	20	0.0344
ATCCAGTTCTCTGAGGGGGCT	16	24	0.0344
TTCCAGCCCTGGTAGGCGCCGCG	16	24	0.0344
ACTGGATTTGGAGCCAGAA	14	19	0.0778
CGCTCTAGGCACCGCAGCA	16	20	0.0778
CACAGCAAGTGTAGACAGGCA	15	20	0.0778
CACTGTGGGTACATGCT	13	20	0.0778
AGCTGTACCTGAAACCAAGCA	16	20	0.0778
ACCTTGCATCTGCATCCCCAG	16	21	0.0778
TGCACGGCACTGGGGACACGT	13	22	0.0778
CTATACGACCTGCTGCCTTTCT	14	23	0.0778
CTACCCTCGGTCTGCTTACCACA	17	23	0.0778
GGAGGCGCAGGCTCGGAAAGGCG	17	27	0.0778
GGGCTGGGGCGCGGGGAGGT	14	20	0.0344
CTCGGGCGGAGGTGGTTGAGTG	14	23	0.0344
AGGCGGGGCGCCGCGGGACCGC	15	23	0.0344
TTGCACTTGTCTCAGTGA	14	20	0.0344
GCTGCACCGGAGACTGGGTAA	14	21	0.0344
GCTGACTCCTAGTCCAGGGCTC	15	21	0.0344
CATTGCACTTGTCTCGGTCTGA	17	22	0.0344
CTGGGTTGGGCTGGGCTGGG	16	20	0.0344
TCACCTGGCTGGCCCGCCCAG	13	25	0.0344
GTGCATTGCTGTTGCATTGC	13	20	0.0344
TTTCCTGTCCTCCAACCAGACC	13	21	0.275
GGCTCCTCCTCTCAGGATGTG	12	22	0.203
TGGGTGGTCTGGAGATTTGTGC	15	24	0.332
TCTGGCTGTTGTGGTGTGCAA	16	22	0.203
TCTGGCTGTGCTGTAATGCAG	14	22	0.203
CCTGTCTGTGCCTGCTGTACA	18	22	0.203
AGGTGTGTCTGTAGAGTCC	16	18	0.0778
GGGGCGCGGCCGGATCG	14	19	0.0778
TGGGCTGCTGAGAAGGGGCA	14	19	0.0778
AAGGGGCAGGGACGGGTGGCCC	14	19	0.0778
CAGGGTCAGCTGAGCATG	12	18	0.143

TGCAGGACCAAGATGAGCCCT	13	19	0.143
TGCTGGGGGCCACATGAGTGTG	15	22	0.143
GGCTGGGTGCTCTTGTGCAGT	14	22	0.143
TGGGCGAGGGCGGCTGAGCGGC	16	24	0.143
GGGGCTGTGATTGACCAGCAGG	15	24	0.143
TTGTTCTTTGGTCTTTCAGCCA	19	21	0.275
AGCGGTGCTCCTGCGGGCCGA	14	19	0.0778
TCTCACTGTAGCCTCGAACCCC	15	20	0.0778
TCACCTGAGCTCCCGTGCCTG	13	20	0.0778
GTCATACACGGCTCTCCTCTCT	16	21	0.0778
CGTCAACACTTGCTGGTTTCCT	15	22	0.0778
AGCCGCTCTTCTCCCTGCCCACA	17	22	0.0778
GGGCTAGGGCCTGCTGCCCCC	15	23	0.0778
AGGGATCGCGGGCGGGTGGCGGCCT	17	24	0.0778
TGGAGTCCAGGAATCTGCATTTT	19	25	0.0778
CAGGCCATATTGTGCTGCCTCA	22	25	0.0778
AAGGGCTTCCTCTCTGCAGGAC	16	25	0.0778
AGGGGGCGCAGTCACTGACGTG	17	25	0.0778
CAGAGTGACAAGCTGGTTAAAG	13	25	0.0778
TCAGGCCAGGCACAGTGGCTCA	19	26	0.0778
TCAGGCCTCTTTCTACCTT	16	26	0.0778
TTCAGATCCCAGCGGTGCCTCT	19	27	0.0778
GTGTCTGCTTCCTGTGGGA	13	19	0.0778
CATTGCACTTGTCTCGGTCTGA	17	23	0.0778
AATTGCACGGTATCCATCTGTA	13	23	0.0778
CACTGTGTCCTTTCTGCGTAG	15	23	0.0778
AAAGATCTGGAAGTGGGAGACA	18	24	0.0866
CCTCACCCAGCTCTCTGGCCCTCT	12	25	0.0778
AAGGGGCTGGGGGAGCACA	16	21	0.0778
TAAAGTGCTGACAGTGCAGAT	14	22	0.0778
TGTCACTCGGCTCGGCCCACTAC	13	23	0.0344
TGAGGTAGTAGATTGTATAGTT	16	22	0.0344
TGGGGCGGAGCTTCCGGAGGCC	15	22	0.0344
CATCTTCCAGTACAGTGTTGGA	14	22	0.203
TGCTGAGGTCCGGGCTGTGCC	14	25	0.143
GGAGGCGCAGGCTCGGAAAGGCG	17	22	0.0344
TGAGGCCCTTGGGGCACAGTGG	18	21	0.0344
TGGGCAGGGGCTTATTGTAGGAG	15	25	0.0344
TGAGGCTAATGCACTACTTCAC	14	19	0.0344
TGGGCGAGGGCGGCTGAGCGGC	12	23	0.0344
TGGGAGAGCAGGGTATTGTGGA	12	23	0.0344
TGGGGAGGTGTGGAGTCAGCAT	17	23	0.0344
TGAGGCTAATGCACTACTTCAC	12	21	0.0648
TCTGGCTGTTGTGGTGTGCAA	17	19	0.0344
TGTCTTCTCTCCTCCCAAACAG	15	25	0.275
TGTCTTTCTTCTCTCCCTTGCAG	14	25	0.275
TCTAGTGCGGGCGTTCCCG	13	19	0.203

CTGACTGTTGCCGTCCTCCAG	16	22	0.203
TCGTGTCTTGTGTTGCAGCCGG	17	23	0.203
ATCTGTCTCGATTGTTTCCAG	15	24	0.203
TTTCTGTCTTTTCTGGTCCAG	19	25	0.203
TTGTCTCTTGTTCCTCACACAG	17	22	0.275
ATTGTCCCTCTCCCTTCCCAG	16	23	0.275
TCTGTGCTTCACCCCTACCCAG	17	23	0.275
AGCTGTCTGTGTTTTCCTTCTCAG	17	24	0.275
AGACTGACGGCTGGAGGCCCAT	14	25	0.275
CAAGCTCGCTTCTATGGGTCTG	16	19	0.284
CAAGCTCGTGTCTGTGGGTCCG	16	19	0.284
CGGGACTGTAGAGGGCATGAGC	12	21	0.0648
GGGGCTGTGATTGACCAGCAGG	17	21	0.0648
TGAGGTAGTAGATTGTATAGTT	12	22	0.0648
TGAGGTAGTAGTTTGTACAGTT	13	22	0.0648
TGGGGCTAGTGATGCAGGACG	14	22	0.0648
TGGGGGTGTGGGGAGAGAGAG	15	22	0.0648
CAGGTCGTCTTGCAGGGCTTCT	13	20	0.284
CCAATATTGGCTGTGCTGCTCC	13	22	0.111
GCTGGTCTGCGTGGTGCTCGG	12	22	0.284
