# Supplementary material for: Dual biomarkers long non-coding RNA GAS5 and microRNA-34a co-expression signature in common solid tumors
Source: PLoS One. 2018 Oct 5;13(10):e0198231. doi: 10.1371/journal.pone.0198231 (PMC6173395; doi:10.1371/journal.pone.0198231)
Supplement: S4 Table — (PDF) [file pone.0198231.s004.pdf]

S4 Table. Complementarity between GAS5 transcripts and miR-34a-5p.

	GAS5 transcript	Trabscript ID	Length (bp)	lncRNA binding sequence
1	ENST00000412059	**GAS5-201**	979	CAAGCCTAACTCAAGCCATTGGCA
2	ENST00000412059	**GAS5-201**	979	CCAATGATGTAATGATTCTGCCA
3	ENST00000414075	**GAS5-202**	413	ACAACAAGCAAGCATGCAGCTT
4	ENST00000416952	**GAS5-203**	799	CAAGCCTAACTCAAGCCATTGGCA
5	ENST00000421068	**GAS5-204**	1060	CAAGCCTAACTCAAGCCATTGGCA
6	ENST00000422008	**GAS5-205**	497	CAAGCCTAACTCAAGCCATTGGCA
7	ENST00000422207	**GAS5-207**	643	ACAACAAGCAAGCATGCAGCTT
8	ENST00000430245	**GAS5-228**	723	ACAACAAGCAAGCATGCAGCTT
9	ENST00000430245	**GAS5-209**	723	CAAGCCTAACTCAAGCCATTGGCA
10	ENST00000431268	**GAS5-210**	1698	CAAGCCTAACTCAAGCCATTGGCA
11	ENST00000431268	**GAS5-210**	1698	CCAATGATGTAATGATTCTGCCA
12	ENST00000432536	**GAS5-211**	959	CAAGCCTAACTCAAGCCATTGGCA
13	ENST00000434796	**GAS5-212**	575	ACAACAAGCAAGCATGCAGCTT
14	ENST00000436285	**GAS5-213**	772	ACAACAAGCAAGCATGCAGCTT
15	ENST00000436656	**GAS5-214**	822	ACAACAAGCAAGCATGCAGCTT
16	ENST00000436656	**GAS5-214**	822	CAAGCCTAACTCAAGCCATTGGCA
17	ENST00000443799	**GAS5-216**	897	ACAACAAGCAAGCATGCAGCTT
18	ENST00000444470	**GAS5-217**	424	ACAACAAGCAAGCATGCAGCTT
19	ENST00000449289	**GAS5-219**	542	ACAACAAGCAAGCATGCAGCTT
20	ENST00000449289	**GAS5-219**	542	CAAGCCTAACTCAAGCCATTGGCA
21	ENST00000449589	**GAS5-220**	712	ACAACAAGCAAGCATGCAGCTT
22	ENST00000449589	**GAS5-220**	712	CAAGCCTAACTCAAGCCATTGGCA
23	ENST00000450589	**GAS5-226**	632	ACAACAAGCAAGCATGCAGCTT
24	ENST00000450589	**GAS5-226**	632	CAAGCCTAACTCAAGCCATTGGCA
25	ENST00000451607	**GAS5-222**	1007	ACAACAAGCAAGCATGCAGCTT
26	ENST00000451607	**GAS5-222**	1007	CAAGCCTAACTCAAGCCATTGGCA
27	ENST00000452197	**GAS5-223**	483	CAAGCCTAACTCAAGCCATTGGCA
28	ENST00000454068	**GAS5-224**	688	ACAACAAGCAAGCATGCAGCTT
29	ENST00000454068	**GAS5-224**	688	CAAGCCTAACTCAAGCCATTGGCA
30	ENST00000455838	**GAS5-221**	632	CAAGCCTAACTCAAGCCATTGGCA
31	ENST00000458220	**GAS5-229**	469	CAAGCCTAACTCAAGCCATTGGCA
32	ENST00000454813	**GAS5-225**	621	CCAATGATGTAATGATTCTGCCA

**Predicted target site**	**miR-34a**	**Predicted miRNA site**
129-153	hsa-miR-34a-5p	TGGCAGTGTCTTAGCTGGTTGT
618-641	hsa-miR-34a-5p	TGGCAGTGTCTTAGCTGGTTGT
205-227	hsa-miR-34a-5p	TGGCAGTGTCTTAGCTGGTTGT
322-346	hsa-miR-34a-5p	TGGCAGTGTCTTAGCTGGTTGT
322-346	hsa-miR-34a-5p	TGGCAGTGTCTTAGCTGGTTGT
88-112	hsa-miR-34a-5p	TGGCAGTGTCTTAGCTGGTTGT
438-460	hsa-miR-34a-5p	TGGCAGTGTCTTAGCTGGTTGT
517-539	hsa-miR-34a-5p	TGGCAGTGTCTTAGCTGGTTGT
260-284	hsa-miR-34a-5p	TGGCAGTGTCTTAGCTGGTTGT
324-348	hsa-miR-34a-5p	TGGCAGTGTCTTAGCTGGTTGT
1337-1350	hsa-miR-34a-5p	TGGCAGTGTCTTAGCTGGTTGT
748-772	hsa-miR-34a-5p	TGGCAGTGTCTTAGCTGGTTGT
264-286	hsa-miR-34a-5p	TGGCAGTGTCTTAGCTGGTTGT
564-286	hsa-miR-34a-5p	TGGCAGTGTCTTAGCTGGTTGT
613-635	hsa-miR-34a-5p	TGGCAGTGTCTTAGCTGGTTGT
131-155	hsa-miR-34a-5p	TGGCAGTGTCTTAGCTGGTTGT
324-348	hsa-miR-34a-5p	TGGCAGTGTCTTAGCTGGTTGT
268-290	hsa-miR-34a-5p	TGGCAGTGTCTTAGCTGGTTGT
333-355	hsa-miR-34a-5p	TGGCAGTGTCTTAGCTGGTTGT
131-155	hsa-miR-34a-5p	TGGCAGTGTCTTAGCTGGTTGT
618-640	hsa-miR-34a-5p	TGGCAGTGTCTTAGCTGGTTGT
324-348	hsa-miR-34a-5p	TGGCAGTGTCTTAGCTGGTTGT
424-446	hsa-miR-34a-5p	TGGCAGTGTCTTAGCTGGTTGT
129-153	hsa-miR-34a-5p	TGGCAGTGTCTTAGCTGGTTGT
932-954	hsa-miR-34a-5p	TGGCAGTGTCTTAGCTGGTTGT
677-701	hsa-miR-34a-5p	TGGCAGTGTCTTAGCTGGTTGT
125-149	hsa-miR-34a-5p	TGGCAGTGTCTTAGCTGGTTGT
490-512	hsa-miR-34a-5p	TGGCAGTGTCTTAGCTGGTTGT
196-220	hsa-miR-34a-5p	TGGCAGTGTCTTAGCTGGTTGT
131-155	hsa-miR-34a-5p	TGGCAGTGTCTTAGCTGGTTGT
129-153	hsa-miR-34a-5p	TGGCAGTGTCTTAGCTGGTTGT
216-239	hsa-miR-34a-5p	TGGCAGTGTCTTAGCTGGTTGT
