# Supplementary material for: Dual biomarkers long non-coding RNA GAS5 and microRNA-34a co-expression signature in common solid tumors
Source: PLoS One. 2018 Oct 5;13(10):e0198231. doi: 10.1371/journal.pone.0198231 (PMC6173395; doi:10.1371/journal.pone.0198231)
Supplement: S5 Table — (PDF) [file pone.0198231.s005.pdf]

S5 Table. hsa-miR-34a pathways.

#	KEGG pathway	KEGG ID	#gene	p-value
**2**	Viral carcinogenesis	hsa05203	27	1.34E-12
**1**	Cell cycle	hsa04110	22	1.00E-16
**5**	Pathways in cancer	hsa05200	20	3.99E-07
**4**	Transcriptional misregulation in cancer	hsa05202	19	3.32E-07
**6**	Pyrimidine metabolism	hsa00240	12	8.19E-07
**7**	Colorectal cancer	hsa05210	11	2.70E-06
**8**	Small cell lung cancer	hsa05222	10	3.31E-06
**11**	Chronic myeloid leukemia	hsa05220	9	6.67E-05
**12**	Pancreatic cancer	hsa05212	9	0.000107253
**14**	Prostate cancer	hsa05215	9	0.000751872
**3**	DNA replication	hsa03030	8	4.03E-08
**15**	p53 signaling pathway	hsa04115	8	0.000786701
**21**	Wnt signaling pathway	hsa04310	8	0.02368349
**9**	Endometrial cancer	hsa05213	7	1.45E-05
**10**	Bladder cancer	hsa05219	7	4.04E-05
**13**	Non-small cell lung cancer	hsa05223	7	0.000270115
**18**	Glioma	hsa05214	7	0.001558798
**19**	Melanoma	hsa05218	6	0.007661737
**16**	Thyroid cancer	hsa05216	5	0.001012695
**20**	Notch signaling pathway	hsa04330	5	0.01841269
**22**	Acute myeloid leukemia	hsa05221	5	0.02492931
