# Supplementary figures and images for: Dual biomarkers long non-coding RNA GAS5 and microRNA-34a co-expression signature in common solid tumors
Source: PLoS One. 2018 Oct 5;13(10):e0198231. doi: 10.1371/journal.pone.0198231 (PMC6173395; doi:10.1371/journal.pone.0198231)

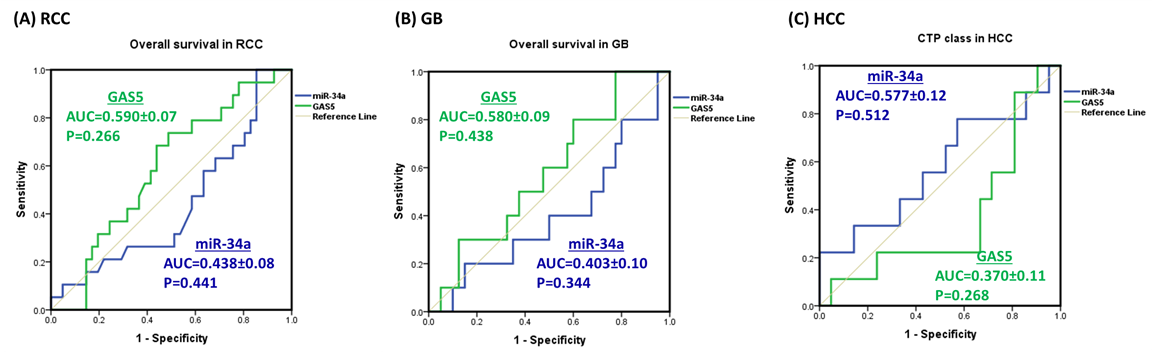

Supplement: S1 Fig — RCC renal cell carcinoma, GB glioblastoma, HCC hepatocellular carcinoma, AUC area under curve, CTP Child-Turcotte-Pugh classification for liver cell failure. (TIF) [file pone.0198231.s006.tif]
